# Inhibition of mitochondrial OMA1 ameliorates osteosarcoma tumorigenesis

**DOI:** 10.1038/s41419-024-07127-1

**Published:** 2024-11-01

**Authors:** Lingyan Chen, Dejian Chen, Yiming Pan, Yimei Mo, Biyu Lai, Huiguang Chen, Da-wei Zhang, Xiao-dan Xia

**Affiliations:** 1 Department of Orthopedics, Affiliated Qingyuan Hospital, Guangzhou Medical University, Qingyuan People’s Hospital, Qingyuan, Guangdong China; 2 Department of Hematology, Dongzhimen Hospital, Beijing University of Chinese Medicine, Beijing, China; 3 Department of Radiology, Affiliated Qingyuan Hospital, Guangzhou Medical University, Qingyuan People’s Hospital, Qingyuan, Guangdong China; 4 Department of Pediatrics, Group on the Molecular and Cell Biology of Lipids, Faculty of Medicine and Dentistry, University of Alberta, Edmonton, AB Canada

**Keywords:** Apoptosis, Bone cancer

## Abstract

OMA1 is an ATP-independent zinc metalloprotease essential for maintaining mitochondrial homeostasis and plays a vital role in tumorigenesis. Depending on the type of cancer, a decrease in OMA1 expression has been linked to a varying prognosis for patients. The role of OMA1 in human osteosarcoma (OS), one of the most prevalent malignant bone tumors, remains elusive. Here, we observed elevated OMA1 expression in OS tumor tissues from four patients with advanced OS. Knockout of OMA1 in OS cells significantly reduces OS tumor weight and size, and lung metastatic nodules in BALB/c nude mice. Immunohistochemistry analysis showed a significant decrease in Ki67 and an increase in Cleaved-caspase 3 in OMA1 knockout tumor samples. Mechanistically, we found that OMA1 deficiency increases the levels of PINK1 and Parkin and consequently induces excessive mitophagy, leading to increased apoptosis and reduced cell proliferation and invasion in OS cells. Specifically, OMA1 deficiency reduces the amount of cytosolic p53 and p53-associated cytosolic Parkin but increases mitochondrial p53, which may lead to enhanced apoptosis. Regarding the effect on cell proliferation and invasion, loss of OMA1 reduces mitochondrial ROS levels and increases cytosolic glycogen synthase kinase 3β (GSK3β) levels, thereby increasing interaction between GSK3β and β-catenin and then reducing cytosolic and nuclear β-catenin. This contributes to reduced cell proliferation and migration in *OMA1-deficient* cells. Moreover, we found that ciclopirox (CPX), an antifungal drug, induces OMA1 self-cleavage and L-OMA1 degradation in cultured OS cells. CPX also reduces tumor development of control OS cells but not *OMA1-deficient* OS cells in mice. These findings strongly support the important role of OMA1 in OS tumorigenesis and suggest that OMA1 may be a valuable prognostic marker and a promising therapeutic target for OS.

## Introduction

Osteosarcoma (OS) is one of the most prevalent malignant bone tumors, predominantly occurring in adolescents [1]. Approximately 15 to 20% of patients develop local invasion and distant lung metastasis [2, 3]. Although the National Cancer Institute’s (NCI) Surveillance, Epidemiology, and End Results (SEER) program shows an overall 5-year survival rate of 60.6–68.1% for OS patients [4], the overall 5-year survival rate for OS patients with distant metastasis is only 20%, and the event-free survival outcomes remain poor at less than 30% [1, 3]. Surveillance screening for OS is ineffective due to the lack of reliable markers and the rarity of OS [1]. Furthermore, OS has significantly high malignant behavior and poor prognosis and is often not amenable to standard therapy strategies and resistant to chemotherapy. Therefore, there is an urgent need to explore the molecular mechanisms of OS tumorigenesis and discover novel and effective therapies to improve the prognosis and outcomes of OS patients.

OMA1, an ATP-independent zinc metalloprotease, is predominantly expressed on the mitochondrial inner membrane and cleaves the mitochondrial fusion protein OPA1 to generate S-OPA1 upon mitochondrial stress [5, 6]. The activation of OMA1 depends on its cleavage to form S-OMA1 (~35 kDa), which, rather than intact OMA1 (~40 kDa), cleaves OPA1 to generate S-OPA1 [7]. OPA1 cleavage upon OMA1 activation limits inner mitochondrial membrane fusion and mitochondrial fragmentation [8]. Recent studies showed that DELE1 is also cleaved by OMA1, which transmits mitochondrial stress to the cytosol [9, 10]. Aberrant expression of OMA1 leads to an imbalance in mitochondrial membrane potential (ΔΨm) and subsequent mitochondrial import arrest of PINK1 [11]. OMA1 is upregulated in several hematologic and solid malignancies, promoting more aggressive and invasive malignant development [12–15]. Consistently, reduced OMA1 expression is linked to better outcomes in patients with certain cancers, such as squamous cell carcinoma [16]. Furthermore, OMA1-mediated integrated stress response prevents ferroptosis in mitochondrial cardiomyopathy [17], suggesting that OMA1 functions differently in a cell type-dependent manner. It has been reported that knockdown of long noncoding RNAs prostate-specific transcript 1 (PCGEM1) inhibits OS cell proliferation and migration likely by reducing OMA1 expression [18], indicating a potential role of OMA1 in OS initiation. However, the exact role and underlying mechanism of OMA1 in OS tumorigenesis and metastasis remain unclear.

Here, we observed increased OMA1 expression in OS tumor tissues collected from four patients with advanced OS. We then investigated the potential role and mechanism of OMA1 in regulating OS tumorigenesis. We found that loss of OMA1 reduced OS volume and size and resulted in smaller lung metastatic nodules in vivo. Mechanistically, we found that knockout of OMA1 promoted apoptosis. OMA1 silencing also inhibited cell proliferation and migration via increasing GSK3β levels and reducing nuclear β-catenin levels.

## Results

### The regulation role of OMA1 in OS tumorigenesis and metastasis in vivo

**Fig. 1:**
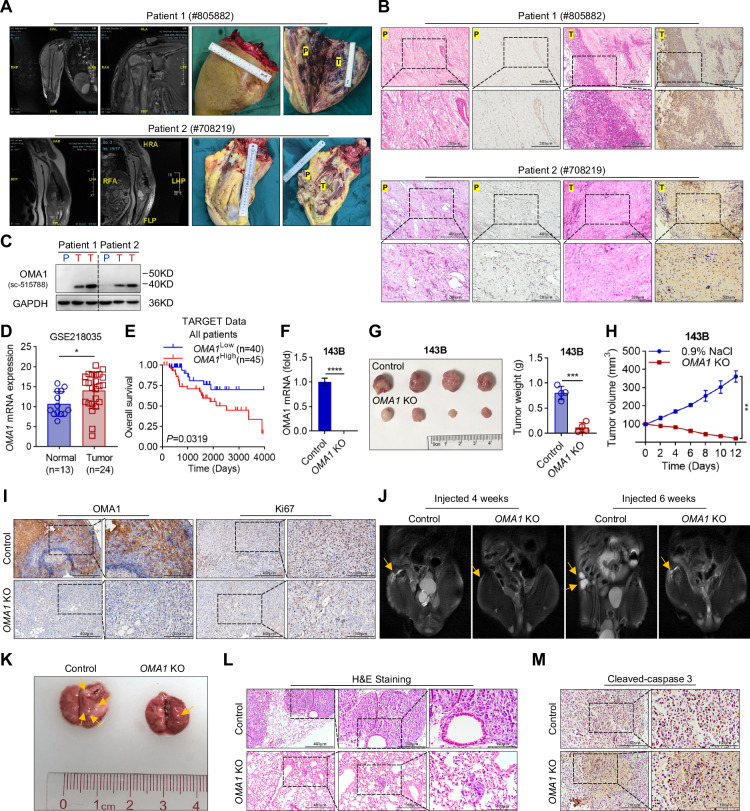
OMA1 is upregulated in human OS tissues and *OMA1* knockout inhibits OS tumorigenesis and lung metastasis. **A** The patients with OS underwent surgical resection of OS tissues based on diagnostic results such as MRI. **B** Histological analysis (H&E) of OS tumors (T) and para-cancerous (P) tissues from patients with OS. The OMA1 levels in OS tissues from tumors and adjacent tissues were analyzed by IHC staining analysis (Scale bars, 400 µm; 200 µm). **C** The samples of tumors and para-cancerous tissues from patients with OS were analyzed by Western blotting with antibodies against OMA1. **D** Relative levels of *OMA1* mRNA in OS tissues compared with adjacent normal tissues were shown (using the GEO dataset GSE218035). Data were shown as mean ± SD and statistical significance was determined by two-tailed unpaired Student’s *t*-test (*P < 0.05). **E** Kaplan–Meier plotter was constructed to analyze and compare patients with high and low levels of *OMA1* in OS samples from the TARGET data. Statistical analysis was performed using log-rank tests. **F** The effectiveness of endogenous *OMA1* stable knockout was measured using RT-qPCR in 143B cells. Data were shown as mean ± SD and statistical significance was determined by two-tailed unpaired Student’s *t*-test (n = 3, ****P < 0.0001). **G** Representative dissected tumors and tumor weight at the end of the experiment derived from control and *OMA1-knockout* 143B cells were shown. Data were shown as mean ± SD and statistical significance was determined by two-tailed unpaired Student’s *t*-test (*n* = 4, ***P < 0.001). **H** Growth of tumor volumes derived from control and *OMA1-deficient* cells was measured once in 2 days. Data were shown as mean ± SD and statistical significance was determined by two-tailed unpaired Student’s *t*-test (*n* = 4, **P < 0.01). **I** Images of IHC staining of Ki67 and OMA1 in tumors of control and *OMA1-deficient* cell xenograft model (Scale bars, 400 µm; 200 µm). **J** Representative MRI images of nude mice with right proximal tibia injected control or *OMA1-deficient* 143B cells for 4 or 6 weeks (*n* = 5). The arrowhead indicates the location of bone lesions and tumors. **K** The bright-field image of metastatic lungs from nude mice injected with control or *OMA1-deficient* 143B cells for 6 weeks, nude mice were sacrificed at 6 weeks (*n* = 5). The arrowhead indicates tumor nodules. **L** H&E staining of the lungs from nude mice injected with control or *OMA1-deficient* 143B cells for 6 weeks (*n* = 5) (Scale bars, 400 µm; 200 µm; 100 µm). **M** IHC analysis of lung sections for Cleaved-caspase 3 expression from nude mice injected with control or *OMA1-deficient* 143B cells for 6 weeks (*n* = 5) (Scale bars, 200 µm; 100 µm).

Given that mitochondrial stress-sensitive peptidase OMA1 controls mitochondrial dynamic balance to maintain bioenergetic homeostasis and mitochondrial dysfunction contributes to tumorigenesis [12, 14, 15], we evaluated OMA1 expression in four OS tumor tissues collected from patients with advanced OS. 3D magnetic resonance imaging (MRI) and surgical resection of tissues revealed the presence of advanced OS in the patients (Fig. 1A). Immunohistochemistry (IHC) analysis showed a marked elevated OMA1 level in tumor tissues (T) compared with para-carcinoma tissues (P) from the same patient (Figs. 1B and S1a, b). Consistent results were observed in Western blotting analysis; the protein level of OMA1 was markedly increased in tumor samples (Fig. 1C). To verify this finding, we performed bioinformatics analysis of human OS samples in a public database GEO Omnibus: GSE218035. We observed that the mRNA levels of *OMA1* were significantly increased in OS samples compared with normal samples (Fig. 1D). Furthermore, Kaplan–Meier survival analysis from the TARGET database revealed that patients with higher levels of OMA1 were correlated with worsened overall survival (*P* = 0.0319) (Fig. 1E). These findings indicate that OMA1 may be a critical regulator of OS tumorigenesis.

To confirm the role of OMA1 in OS tumorigenesis and metastasis, we established *OMA1* stable knockout 143B cells using CRISPR/Cas9, and *OMA1* knockout was confirmed by RT-qPCR (Fig. 1F). Control and *OMA1* knockout cells were injected subcutaneously into BALB/c nude mice. OMA1 deficiency significantly reduced OS tumor weight and size (Fig. 1G, H). IHC analysis showed that OMA1 was essentially undetectable, and Ki67 decreased markedly in *OMA1* knockout tumor samples (Fig. 1I), indicating that lack of OMA1 reduces OS cell proliferation and tumorigenesis in vivo. To further confirm these findings, we generated a control- and *OMA1* deletion-situ xenograft nude mice model. MRI was conducted on the right proximal tibia of nude mice at 4 and 6 weeks after injection of *OMA1-deficient* or control 143B cells. Images showed that the OS volume was smaller in the *OMA1-deficient* group than in the control group (Fig. 1J, arrowed). Given that the activity of OMA1 is dependent on its self-cleavage [7], we stably expressed mouse *OMA1*-Flag or its catalytically dead mutant E324Q in *OMA1-deficient* 143B cells. *OMA1*-Flag but not *OMA1*-E324Q-Flag produced a cleaved S-OMA1 band, indicating that OMA1 undergoes autocleavage in 143B cells (Fig. S1c). Interestingly, expression of wild-type OMA1 restored tumor growth of *OMA1-knockout* 143B cells, whereas expression of *OMA1*-E324Q-Flag had no effect (Fig. S1d, arrowed), indicating the requirement of the catalytical activity of OMA1 for promoting OS growth. Knockout of OMA1 also resulted in smaller lung metastatic nodules (Fig. 1K). Consistently, lung tissues from the control group showed a marked increase in metastatic nodules compared with that from the *OMA1-deficient* group (Fig. 1L). On the other hand, loss of OMA1 markedly increased apoptosis in the metastatic lung tissues, as evidenced by increased Cleaved-caspase3 staining (Fig. 1M), indicating that OMA1 regulates apoptosis, playing a critical role in OS tumorigenesis and lung metastasis.

### Effect of OMA1 deficiency on apoptosis of OS cells

**Fig. 2:**
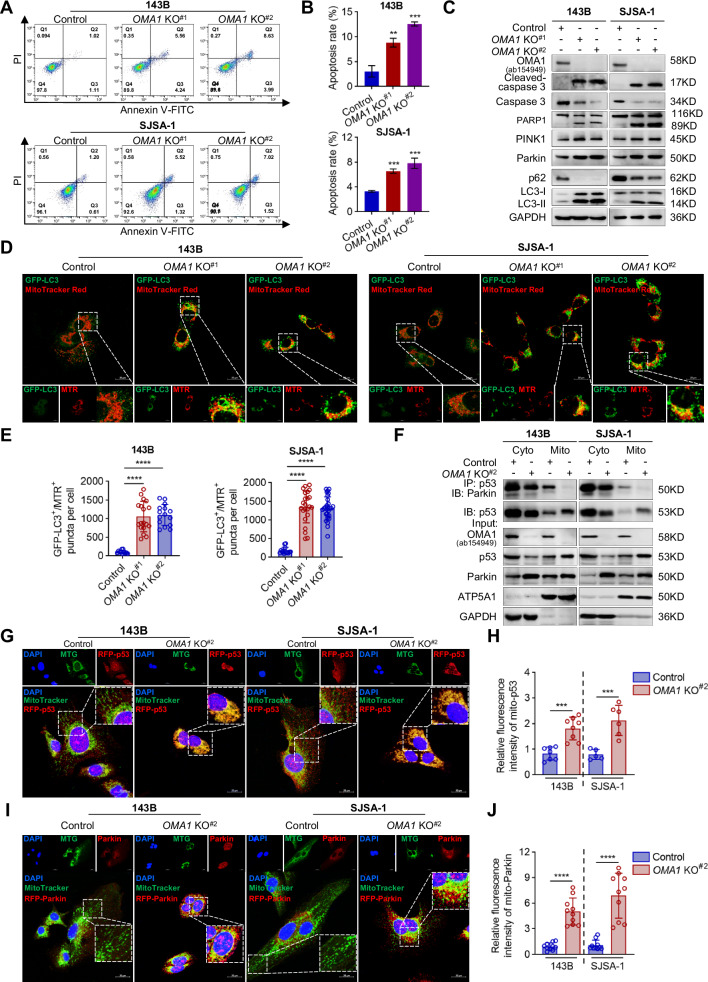
Loss of OMA1 triggers apoptosis of OS cells. **A**, **B** Control and *OMA1-deficient* 143B and SJSA-1 stained with annexin V-FITC/PI were determined using flow cytometry (**A**). The apoptosis rates were plotted followed by statistical analysis (**B**). Data were shown as mean ± SD, and One-way ANOVA assessed statistical significance with Bonferroni post-test and two-tailed unpaired Student’s *t*-test (*n* = 3, **P < 0.01, ***P < 0.001). **C** Western blotting analysis of apoptosis- and mitophagy-related proteins in control and *OMA1-deficient* OS cells. **D**, **E** Representative confocal microscopy images of GFP-LC3 expressing control or *OMA1-deficient* cells before the immunostaining of mitochondria with MitoTracker Red (MTR) (Scale bar, 20 µm) (**D**). The GFP-LC3^+^/MTR^+^ puncta per cell was quantified using ImageJ Plus software (**E**). Data were shown as mean ± SD, and One-way ANOVA assessed statistical significance with Bonferroni post-test and two-tailed unpaired Student’s *t*-test (*n* > 3, ****P < 0.0001). **F** Endogenous p53 interaction with Parkin in the cytoplasm and mitochondria was immunoprecipitation with p53 antibody. Representative confocal microscopy images of overexpression of RFP-p53 (**G**) or RFP-Parkin (**I**) before staining with MitoTracker Green (MTG) and DAPI. The relative fluorescence intensity of mitochondrial p53 (**H**) or Parkin (**J**) was quantified using ImageJ Plus software (Scale bar, 20 µm). Data were shown as mean ± SD and statistical significance was assessed by two-tailed unpaired Student’s *t*-test (*n* > 3, ***P < 0.001, ****P < 0.0001).

We then used two cultured OS cell lines, 143B and SJSA-1 to evaluate whether and how OMA1 deficiency affected apoptosis. To minimize potential off-target effects, we selected two *OMA1* stable knockout clones per cell line for the Annexin-FITC/PI apoptosis assay and flow cytometry. OMA1 deletion significantly promoted apoptosis in 143B and SJSA-1 cells, as evidenced by the increased number of Annexin V-FITC-stained cells (Fig. 2A, B). Consistently, transient knockdown of OMA1 in 143B and SJSA-1 cells using siRNA also significantly increased apoptosis (Fig. S2a–c). Cleaved PARP1 (~89 kDa) and Cleaved-caspase3 levels were also increased in *OMA1* knockout OS cells (Fig. 2C). Therefore, consistent with findings in tumor samples (Fig. 1M), OMA1 deficiency enhances apoptosis in OS cells.

Next, we investigated the underlying mechanism. It has been reported that OMA1 cooperates with mitochondrial inner protein Tim23 to stimulate PINK1 activation and accumulation at the outer mitochondrial membrane (OMM) [11], and mitophagy can drive apoptosis [19]. Therefore, we examined the levels of mitophagy-related proteins to evaluate whether *OMA1* deficiency-induced apoptosis was stimulated by mitophagy. As shown in Fig. 2C, silencing of OMA1 dramatically increased PINK1 and Parkin levels and the ratio of LC3-II/LC3-I, but decreased the level of p62, a well-known autophagic substrate. We then labeled OS cells with GFP-tagged LC3, MitoTracker, and LysoTracker. We found that colocalization of GFP-LC3^+^ with MitoTracker Red^+^ and MitoTracker Green^+^ with LysoTracker Red^+^ was more abundant in *OMA1-deficient* 143B and SJSA-1 cells compared with control cells (Figs. 2D, E and S2d, e), implying increased formation of mitochondrial autophagosomes and autolysosomes upon silencing of OMA1.

How does OMA1 regulate PINK1 and Parkin levels? Cytosolic p53 has been reported to bind to Parkin and inhibit its translocation to mitochondria, thereby weakening mitophagy [20, 21]. Additionally, p53 can be rapidly transported to mitochondria under stress conditions, leading to the release of pro-apoptotic factors from the mitochondrial intermembrane space [20, 21]. We found that cytosolic and mitochondrial p53 coimmunoprecipitated with Parkin in the control group (Fig. 2F). In contrast, the interaction of p53 with Parkin in the cytosolic and mitochondrial fractions was drastically decreased when OMA1 was knocked out, even though OMA1 deficiency increased the levels of cytosolic and mitochondrial Parkin and mitochondrial p53 (Fig. 2F). Consistently, we observed increased accumulation of RFP^+^-tagged p53 in the mitochondria of *OMA1-deficient* cells compared with that of control cells (Fig. 2G, H). This may lead to a decrease in cytosolic p53 in *OMA1-deficient* cells (Fig. 2F), weakening the inhibitory effect of cytosolic p53 on Parkin. Parkin can then be translocated to mitochondria to enhance mitophagy. Indeed, we observed increased mitochondrial Parkin in *OMA1-deficient* cells (Fig. 2F, I, J).

To investigate whether prolonged mitophagy activation leads to apoptosis in *OMA1-deficient* OS cells, we treated cells with autophagy inhibitors, Bafilomycin A1 (BafA1), Chloroquine (CQ), and 3-methyladenine (3-MA). We found that these autophagy inhibitors significantly reduced apoptosis triggered by silencing OMA1 in 143B and SJSA-1 cells (Fig. S3a–f). Moreover, 3-MA efficiently inhibited Caspase 3 cleavage induced by lack of OMA1 (Fig. S3g). To further confirm these findings, we silenced the expression of Parkin in *OMA1* knockout cells using siRNA and observed efficient knockdown of Parkin (Fig. S3h). Consistently, the knockdown of Parkin significantly reduced the increase in apoptosis induced by *OMA1* knockout (Fig. S3i, j). Collectively, these data indicate that OMA1 deficiency causes p53 mitochondrial translocation and subsequently reduces cytosolic p53 levels and the interaction between cytosolic p53 and Parkin, which may reduce the inhibitory effect of cytosolic p53 on Parkin and consequently leads to an increase in mitochondrial Parkin levels. This ultimately exacerbates mitophagy, which contributes, at least in part, to increased apoptosis.

### Effect of OMA1 deficiency on OS cell proliferation

**Fig. 3:**
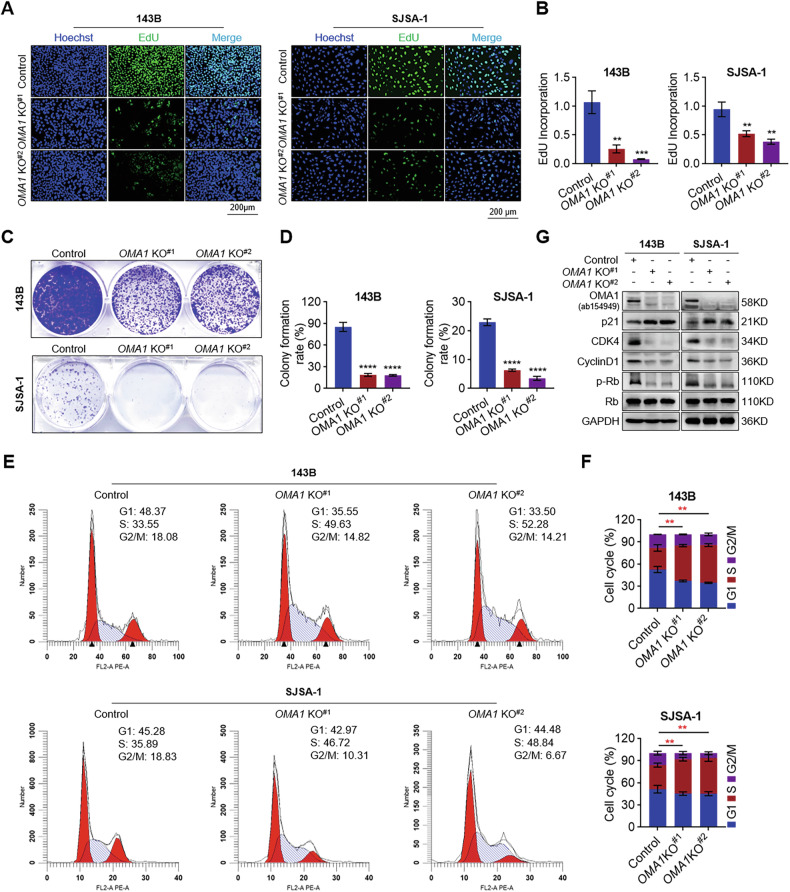
OMA1 is critical for OS cell proliferation. Cell proliferation of control and *OMA1* stable knockout 143B and SJSA-1 cells was measured using an EdU cell proliferation kit with Alexa Fluor 488 (**A**) (Scale bar, 200 µm). EdU incorporation was quantified using ImageJ Plus software (**B**). Data were shown as mean ± SD, and One-way ANOVA assessed statistical significance with Bonferroni post-test and two-tailed unpaired Student’s *t*-test (*n* = 3, **P < 0.01, ***P < 0.001). Representative images (**C**) and quantification (**D**) of colony formation assay of control or *OMA1-deficient* cells. Data were shown as mean ± SD, and One-way ANOVA assessed statistical significance with Bonferroni post-test and two-tailed unpaired Student’s *t*-test (*n* = 3, ****P < 0.0001). Representative cell cycle curves (**E**) and the percentage of cell cycle distribution (**F**) were analyzed using flow cytometry and ModiFit LT 5.0. Data were shown as mean ± SD, and One-way ANOVA assessed statistical significance with Bonferroni post-test and two-tailed unpaired Student’s *t*-test (*n* = 3, **P < 0.01). **G** The levels of cell cycle-related proteins were examined using Western blotting analysis.

Tumorigenesis is a complex process. Findings in Fig. 1I showed that Ki67 levels were markedly reduced in *OMA1* knockout tumor samples, indicating the potential impact on OS cell proliferation. To gain a more comprehensive understanding of the role of OMA1 in OS tumorigenesis, we first evaluated the effect of OMA1 deficiency on OS proliferation. EdU incorporation assay revealed that knockout of OMA1 significantly inhibited the proliferation of 143B and SJSA-1 cells (Fig. 3A, B). Colony formation was also significantly reduced in *OMA1-deficient* cells compared with control cells (Fig. 3C, D). Furthermore, we assessed the cell cycle distribution of OS cells using flow cytometry and found that loss of OMA1 triggered S phase arrest (Fig. 3E, F). Consistently, the protein levels of CDK4, CyclinD1, and p-Rb/Rb were downregulated, while p21 was significantly increased in *OMA1-deficient* cells (Fig. 3G). Therefore, OMA1 deficiency suppresses OS cell proliferation, probably by arresting the cell cycle.

### Effects of OMA1 deficiency on GSK3β and β-catenin

**Fig. 4:**
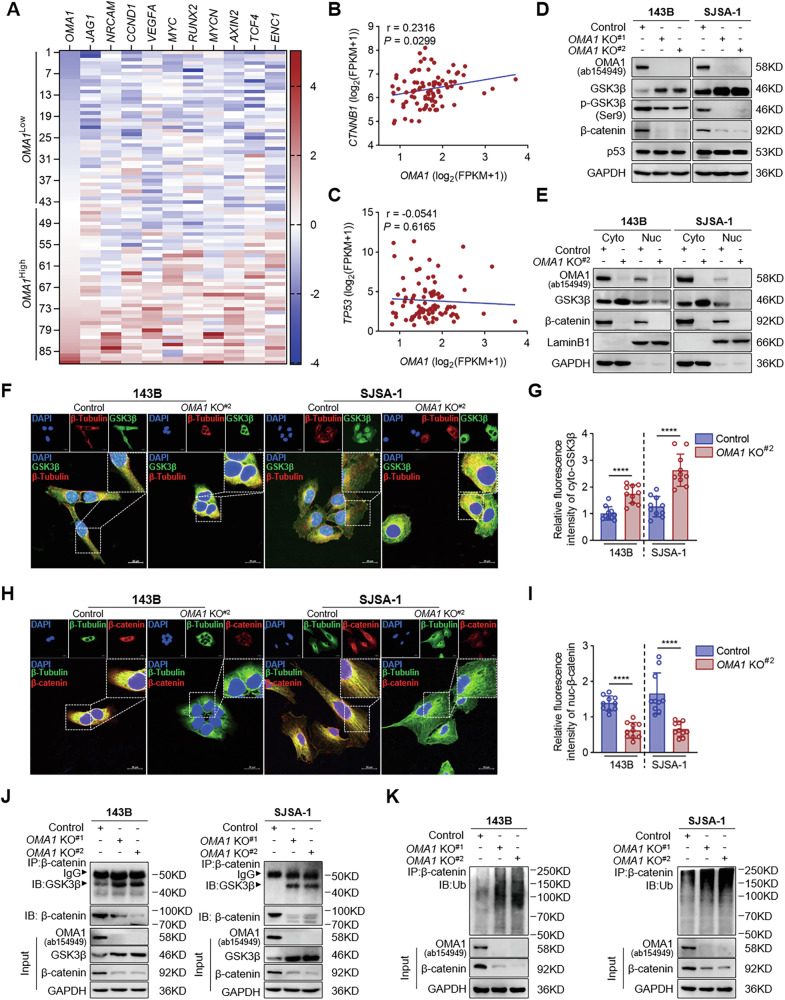
Loss of OMA1 reduces cytosolic GSK3β-mediated β-catenin nuclear translocation to promote its ubiquitin-dependent degradation within the cytoplasm. **A** The correlation between *OMA1* and *CTNNB1* target genes was analyzed by R language pheatmap (*n* = 88). log_2_ (FPKM + 1) normalized TARGET values and calculated the Z-score. Graphs for correlation analysis indicate the correlation between *OMA1* and *CTNNB1* (**B**) or *TP53* (**C**) (*n* = 88). log_2_ (FPKM + 1) normalized TARGET values. **D** The whole cell lysates of *OMA1-deficient* cells were determined by Western blotting with antibodies against GSK3β, p-GSK3β, β-catenin, or p53. **E** The protein levels of GSK3β and β-catenin in the cytoplasm (Cyto) and nucleus (Nuc) of *OMA1-deficient* cells were analyzed by nuclear/cytosolic fractionation and Western blotting. Representative confocal microscopy images of the co-localization of β-tubulin and cytosolic GSK3β (**F**) or nuclear β-catenin (**H**) in *OMA1-deficient* cells. The relative fluorescence intensity of cytosolic GSK3β (**G**) or nuclear β-catenin (**I**) was quantified using ImageJ Plus software (Scale bar, 20 µm). Data were shown as mean ± SD and statistical significance was assessed by two-tailed unpaired Student’s *t*-test (*n* > 3, ****P < 0.0001). **J** Endogenous GSK3β interaction with β-catenin in the cytoplasm was immunoprecipitation with β-catenin antibody. **K** These samples were subjected to immunoprecipitation with β-catenin antibody to evaluate its ubiquitination levels in *OMA1-deficient* cells.

Next, we dissected the detailed molecular mechanisms by which OMA1 regulates cell proliferation. We observed that knockout OMA1 markedly reduced cytosolic p53 (Fig. 2F), which might increase cell proliferation given the tumor suppressor activity of p53 [22, 23]. However, we observed reduced cell proliferation in *OMA1-deficient* OS cells, indicating that mechanisms other than p53 may be responsible for this effect. Therefore, we comprehensively analyzed the transcriptomes of *OMA1*^Low^ and *OMA1*^High^ OS patient samples from the TARGET database. As shown in Fig. 4A, a series of β-catenin target genes showed a downward trend in *OMA1*^Low^ OS tumors, but their expression was upregulated in *OMA1*^High^ OS tumors. Moreover, *CTNNB1* expression, but not *TP53*, was positively correlated with *OMA1* expression in OS (*r* = 0.2316, *P* = 0.0299) (Fig. 4B, C), suggesting that OMA1 might regulate the β-catenin signaling pathway in OS cell proliferation. As GSK3β synergizes with its downstream β-catenin to regulate tumor cell proliferation [24, 25], we investigated the effect of OMA1 depletion and overexpression on GSK3β and β-catenin expression in OS cells. We found that knockout of OMA1 markedly increased the levels of total GSK3β but decreased the levels of the inactive form of GSK3β phosphorylated at Ser 9 (Fig. 4D). β-catenin levels were also reduced, but p53 levels were unchanged in *OMA1-deficient* cells (Fig. 4D). On the other hand, overexpression of OMA1 consistently reduced total GSK3β and increased phosphorylated GSK3β and β-catenin (Fig. S4a). OMA1 undergoes autocleavage, a process critical for OMA1 activation and degradation [7, 26]. Therefore, we also expressed catalytically dead mutant *OMA1*-E324Q-Flag in *OMA1* deficiency OS cells. We did observe a cleaved S-OMA1 band at 35 kDa in control cells that expressed endogenous OMA1 (Fig. S4b, lanes 1 and 3). Expression of wild-type *OMA1*-Flag but not *OMA1*-E324Q-Flag in *OMA1* knockout cells produces a cleaved band with a molecular mass slightly larger than 35 kDa, probably due to the presence of the Flag tag (Fig. S4b, lanes 2 vs 4). Blotting the membrane with an anti-Flag antibody showed that wild-type *OMA1*-Flag but not *OMA1*-E324Q-Flag produced a cleaved OMA1 band, indicating that OMA1 undergoes autocleavage in 143B and SJSA-1 cells. We also found that expression of wild-type *OMA1*-Flag but not *OMA1*-E324Q-Flag substantially blocked *OMA1* knockout-induced increase in total GSK3β, as well as a decrease in phosphorylated GSK3β and β-catenin (Fig. S4b, lanes 2 vs 4), indicating the requirement of the catalytical activity of OMA1.

How does OMA1, as a mitochondrial inner membrane protein, regulate the levels of cytosolic GSK3β? It has been reported that ROS production increases Ser-9 phosphorylation of GSK3β [27–29], and Ser-9 phosphorylation of GSK3β not only inhibits its activity but also induces GSK3β degradation [30]. Ser-9 phosphorylation of GSK3β was reduced in *OMA1* knockout cells (Fig. 4D). Therefore, we examined whether ROS production was affected in *OMA1* knockout cells. As shown in Fig. S4c, d, silencing of OMA1 significantly reduced mitochondrial ROS levels in both 143B and SJSA-1 cells. We then increased mitochondrial ROS production in cells using Retenone and found that it significantly blocked *OMA1* deficiency-induced increase in total GSK3β levels and reduction in the p-GSK3β (Ser9) and β-catenin (Fig. S4e). Therefore, OMA1 deficiency reduces mitochondrial ROS levels, which may contribute to the reduction in Ser-9 phosphorylation of GSK3β and subsequent increased GSK3β levels.

To further confirm the effect of OMA1 deficiency on GSK3β and β-catenin levels, we conducted cell fractionation and observed that OMA1 deficiency led to accumulation of GSK3β in the cytoplasm, reduction of GSK3β in the nucleus, and reduction of β-catenin in the cytoplasm and nucleus (Fig. 4E). Opposite phenotypes were observed in *OMA1-overexpressing* cells (Fig. S5a). We also performed confocal microscopy and observed that OMA1 knockout increased cytosolic GSK3β and reduced nuclear β-catenin (Fig. 4F–I), while overexpression of OMA1 displayed an opposite phenotype (Fig. S5b–e). Collectively, these findings indicate that OMA1 reduces cytoplasmic GSK3β and increases nuclear β-catenin.

Considering that cytoplasmic GSK3β phosphorylates β-catenin to promote its degradation via the ubiquitin-proteasome pathway [30, 31], we hypothesized that OMA1 might protect β-catenin from GSK3β-mediated protein degradation, promoting OS proliferation. To test this possibility, we immunoprecipitated β-catenin from the cytosolic fraction and observed that GSK3β co-immunoprecipitated with β-catenin (Fig. 4J). OMA1 knockout increased the amount of GSK3β coimmunoprecipitated with β-catenin, despite that OMA1 knockout reduced the levels of β-catenin in the cytosolic fraction (Input) and immunoprecipitated fraction (Fig. 4J). Consistently, ubiquitination of cytosolic β-catenin was significantly increased when OMA1 was knocked out (Fig. 4K). Taken together, these findings indicate that OMA1 deficiency increases GSK3β levels, enhances the interaction of cytosolic GSK3β with β-catenin, and increases ubiquitination of β-catenin, thereby reducing nuclear β-catenin levels and subsequent cell proliferation.

### Effect of OMA1 deficiency on OS cell migration and invasion

The GSK3β/β-catenin signaling pathway regulates tumor cell proliferation as well as tumor cell migration and invasion under various circumstances [32, 33]. Therefore, we investigated the impact of OMA1 deficiency on OS metastasis and invasion, an important driver of high mortality in patients with OS. We observed that silencing of OMA1 significantly inhibited 143B and SJSA-1 cell migration and invasion (Fig. S6a–d). Since epithelial-mesenchymal transition (EMT) plays a pivotal role in aggravating metastasis and invasion of cancer cells [34], we assessed the expression of the epithelial marker E-Cadherin and the mesenchymal markers fibronectin and vimentin. As shown in Fig. S6e, OMA1 deficiency markedly decreased the expression of pre-metastatic and pro-invasive proteins, fibronectin and vimentin, while increasing the levels of anti-metastatic protein E-Cadherin. These results indicate that loss of OMA1 alters EMT expression and inhibits migration and invasion of OS cells.

**Fig. 5:**
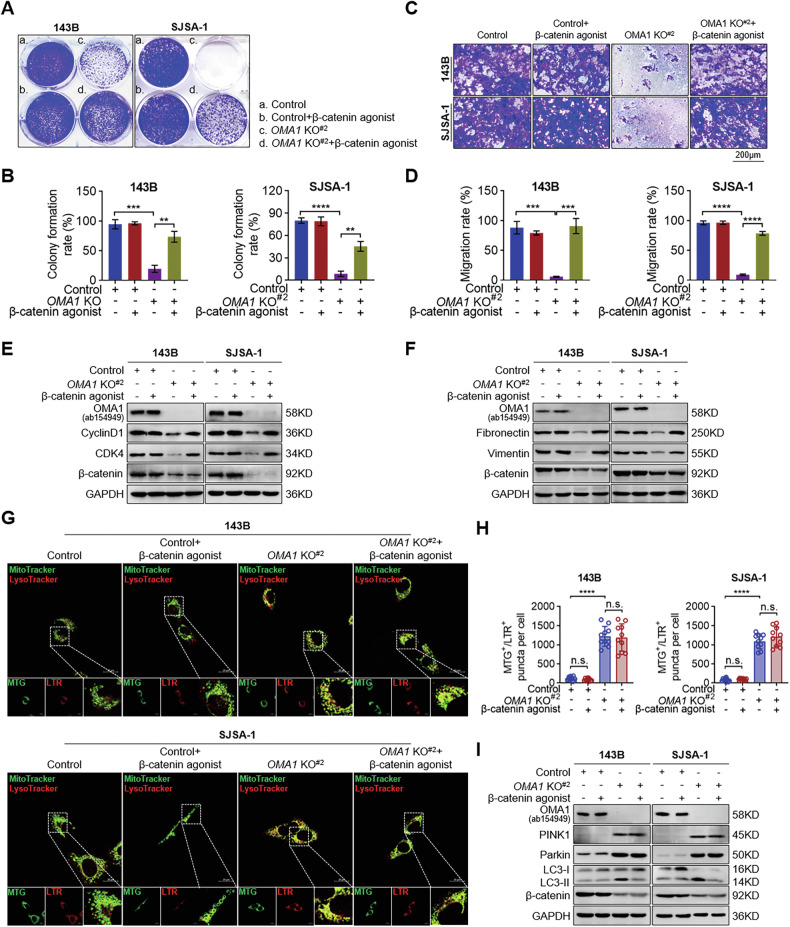
Ablation of OMA1 suppresses OS cell proliferation and migration through β-catenin. **A**, **B** Colony formation of *OMA1-deficient* cells treated with β-catenin agonist (10 µM) for 1–2 weeks, and cell colonies were stained with 0.1% crystal violet solution (**A**). Colony numbers were counted using ImageJ Plus software (**B**). Data were shown as mean ± SD, and statistical significance was assessed by two-tailed unpaired Student’s *t*-test (*n* = 3, **P < 0.01, ***P < 0.001, ****P < 0.0001). Cell migration activity of *OMA1-deficient* cell lines treated with β-catenin agonist (10 µM) for 48 h was measured using Transwell migration assay (**C**), and quantification of cell migration rate was determined using ImageJ Plus software (**D**). Migrated cells were stained with 0.1% crystal violet solution and then imaged with a light microscope (Scale bar, 200 µm). Data were shown as mean ± SD, statistical significance was analyzed by two-tailed unpaired Student’s *t*-test (*n* = 3, ***P < 0.001, ****P < 0.0001). Western blotting analysis of cell cycle-related (**E**) and cell migration- and invasion-related (**F**) protein levels in *OMA1-deficient* cells treated with β-catenin agonist (10 µM) for 12 h. Representative images of the co-localization of MitoTracker Green (MTG) and LysoTracker Red (LTR) in *OMA1* knockout cells treated with β-catenin agonist (10 µM) for 12 h (**G**) (Scale bar, 20 µm). The MTG^+^/LTR^+^ puncta per cell was quantified using ImageJ Plus software (**H**). Data were shown as mean ± SD, statistical significance was analyzed by two-tailed unpaired Student’s *t*-test (*n* > 3, ****P < 0.0001, or n.s., not significant). **I** Western blotting analysis of mitophagy-related protein levels in *OMA1-deficient* cells treated with β-catenin agonist (10 µM) for 12 h.

To further validate these findings, we treated control and *OMA1-deficient* OS cells with a β-catenin agonist and then evaluated cell proliferation, migration, mitophagy, and apoptosis. The β-catenin agonist markedly increased colony formation and cell migration of *OMA1-deficient* cells but had no significant effect on control cells (Fig. 5A–D). Consistently, the β-catenin agonist reversed the inhibitory effect of OMA1 deficiency on the expression of endogenous Cyclin D1 and CDK4, as well as fibronectin and vimentin (Fig. 5E, F). On the other hand, the agonist did not significantly affect the formation of mitochondrial autolysosomes and the increase in PINK1 and Parkin expression in *OMA1-deficient* OS cells (Fig. 5G–I). The β-catenin agonist also had no significant effect on *OMA1* knockout-induced apoptosis, increased in Cleaved-caspase 3, and decreased in β-catenin in 143B and SJSA-1 cells (Fig. S7a–c). These findings indicate that β-catenin is critical in *OMA1* deficiency-induced cell proliferation and migration. However, its role in *OMA1* deficiency-induced mitophagy and apoptosis in OS cells is negligible.

### Ciclopirox induces OMA1 self-cleavage and L-OMA1 degradation to inhibit OS tumorigenesis

**Fig. 6:**
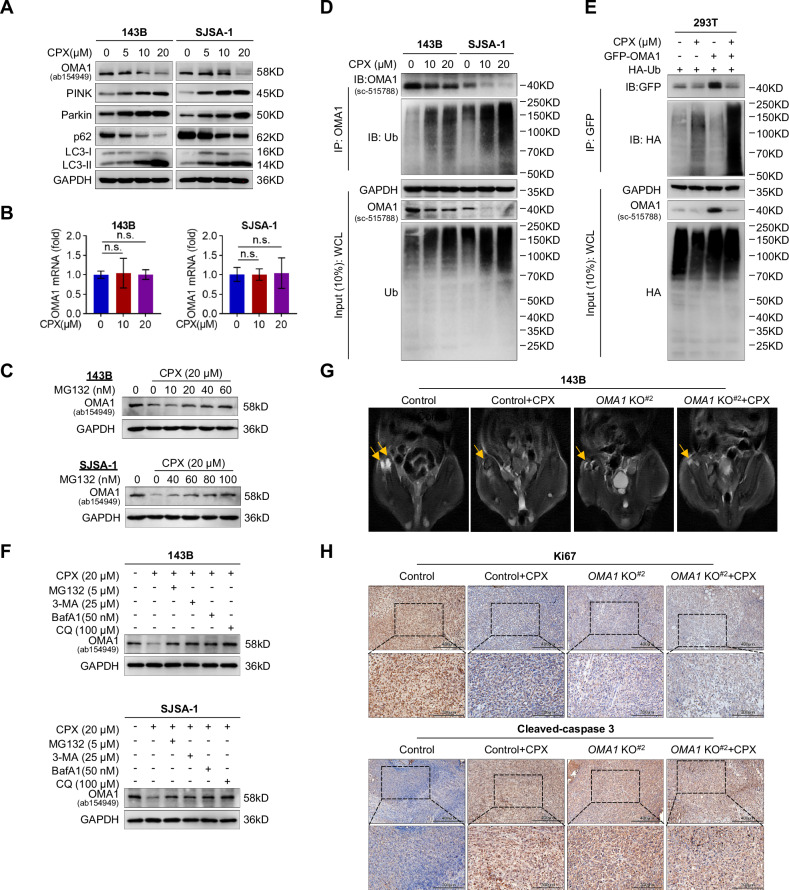
CPX induces L-OMA1 degradation via ubiquitination and lysosome pathways. **A** Western blotting analysis of OMA1 and mitophagy-related proteins in OS cells treated with CPX for 24 h. **B** RT-qPCR analysis of *OMA1* mRNA levels in OS cells treated with CPX for 24 h. Data were shown as mean ± SD, and One-way ANOVA assessed statistical significance with Bonferroni post-test and two-tailed unpaired Student’s *t*-test (*n* = 3, n.s., not significant). **C** Western blotting analysis of OMA1 protein in OS cells co-treated with CPX (20 µM) and a serial dose of MG132 for 24 h. **D** Endogenous ubiquitination levels of OMA1 in OS cells treated with CPX for 24 h were determined using an immunoprecipitation assay. **E** The whole cell lysates of HEK 293T cells treated with 10 µM CPX following co-transfected with GFP-tagged OMA1 and HA-tagged Ub were immunoprecipitated with the GFP tag antibody and blotted with HA tag antibody. **F** Western blotting analysis of OMA1 protein in OS cells co-treated with CPX and MG132, or autophagy inhibitors for 24 h. **G** Representative MRI images of nude mice with right proximal tibia injected control or *OMA1-deficient* 143B cells for 4 weeks were intraperitoneally injected with 0.9% NaCl or CPX (20 mg/kg) for 2 weeks (*n* = 3). The arrowhead indicates the location of bone lesions and tumors. **H** Immunohistochemical analysis of Ki67 and Cleaved-caspase 3 in the OS xenograft intraperitoneally injected with 0.9% NaCl or CPX (*n* = 3) (Scale bars, 400 µm; 200 µm).

We have previously reported that ciclopirox (CPX), an antifungal drug, activates mitophagy-related proteins, such as PINK1 and Parkin, in gastric cancer cells [35]. Here, we investigated its effect on OS cells and observed that CPX within IC_50_ concentration significantly increased the colocalization of MitoTracker with LysoTracker, indicating increased mitophagy (Fig. S8a–c). Furthermore, CPX reduced OMA1 levels, increased the levels of PINK, Parkin, and LC3-II, and reduced p62 levels, but did not affect *OMA1* mRNA levels (Fig. 6A, B), implying that CPX regulates OMA1 expression at the post-translational level. It has been reported that the ubiquitin-proteasome pathway is mainly responsible for OMA1 degradation [36, 37]. Therefore, we treated OS cells with CPX and Z-Leu-Leu-Leu-al (MG132), a proteasome inhibitor. MG132 indeed inhibited CPX-induced reduction in OMA1 protein levels in a dose-dependent manner (Fig. 6C). Furthermore, CPX markedly increased ubiquitination of endogenous and overexpressed L-OMA1 (40 kDa) (Fig. 6D, E), indicating that CPX promotes L-OMA1 degradation via the ubiquitin-proteasome pathway. We also observed that autophagy inhibitor 3-MA remarkably increased OMA1 protein levels in OS cells (Fig. S3g), suggesting that OMA1 protein may also be degraded in a lysosome-dependent manner. Therefore, we treated OS cells with autophagy inhibitors, including 3-MA, BafA1, and CQ. As shown in Fig. 6F and Fig. S8d, all these inhibitors blocked CPX-induced L-OMA1 degradation, while autophagy activator Rapamycin (RAPA) reduced L-OMA1 levels, indicating that OMA1 may also be degraded through the lysosome pathway. Furthermore, we co-stained OS cells with OMA1, LAMP1, and a fluorescent mitochondrial dye MitoTracker. We observed that OMA1 in mitochondria was transferred to lysosomes with CPX treatment (Fig. S8e, arrowed). These data suggest that CPX degrades OMA1 protein through the ubiquitination and lysosome pathways.

OMA1 is activated in response to various stresses, and its self-cleavage positively correlates with OMA1 activation and degradation [7, 26]. Qi et al. reported that CPX induces endoplasmic reticulum stress and impairs mitochondrial function in colorectal cancer cells [38]. We thus evaluated whether CPX affects OMA1 self-cleavage and its activity. After CPX treatment, endogenous L-OMA1 protein bands (40 kDa) were reduced; S-OMA1 bands at 35 kDa were accumulated, indicating that OMA1 cleavage is enhanced upon CPX treatment (Fig. S9a). OPA1 is cleaved by S-OMA1 rather than L-OMA1 [7]. Consistently, CPX substantially increased OPA1 cleavage in 143B and SJSA-1 cells with or without OMA1 overexpression (Fig. S9a). This leads to OPA1 inactivation and subsequent mitochondrial fragmentation and apoptosis [7, 8]. These findings also suggest that CPX may enhance OMA1 activity even though it promotes the degradation of L-OMA1 (40 kDa) (Fig. 6D, E). To further confirm the effect of CPX on OMA1 cleavage, we overexpressed mouse *OMA1*-Flag or its mutant *OMA1*-E324Q-Flag in *OMA1-deficient* 143B and SJSA-1 cells and analyzed OMA1 protein levels and itself cleavage by Western blot using anti-OMA1 or anti-Flag antibody after CPX treatment (Fig. S9b). We observed a cleaved S-OMA1 band in *OMA1-deficient* 143B and SJSA-1 cells overexpressing *OMA1*-Flag, whereas no S-OMA1 was observed in cells overexpressing catalytically dead *OMA1*-E324Q-Flag, suggesting the presence of self-processing of OMA1 in these cells (Fig. S9b). Interestingly, CPX increased the level of S-OMA1 in cells expressing *OMA1*-Flag (Fig. S9b). Therefore, CPX appears to promote the degradation of L-OMA1 through the ubiquitination and lysosome pathways and increases the levels of S-OMA1, probably by enhancing its self-cleavage, as CPX does not promote OMA1 cleavage in cells expressing *OMA1*-E324Q-Flag. Additionally, EdU incorporation and colony formation experiments showed that CPX significantly reduced the growth of *OMA1-overexpressing* cells. The growth of control cells that express endogenous OMA1 was also inhibited by CPX, but to a much lesser extent than that of *OMA1-overexpressing* cells (Fig. S9c–f).

To further determine whether CPX inhibits OS tumorigenesis through OMA1, we obtained MRI images of the right proximal tibia of nude mice at 6 weeks after injection of *OMA1-deficient* or control 143B cells with or without CPX treatment. We found that CPX treatment significantly suppressed OS growth compared with the control group (Fig. 6G), but CPX treatment did not change the *OMA1* deficiency-induced inhibition of OS volume (Fig. 6G). Consistently, IHC analysis showed that CPX reduced Ki67 and increased Cleaved-caspase 3 when compared with the control group. However, the reduction of proliferation (Ki67 staining) and the increase of apoptosis (Cleaved-caspase 3 staining) in the *OMA1-deficient* group were not affected by CPX treatment (Fig. 6H). These findings indicate that CPX inhibits OS tumorigenesis through a mechanism involving OMA1.

## Discussion

**Fig. 7:**
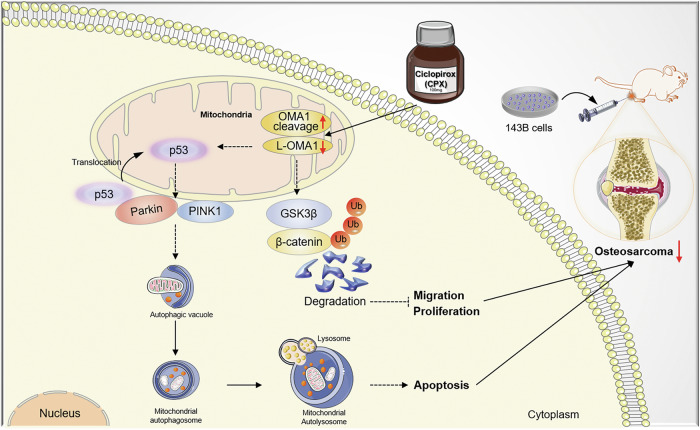
Schematic depicting the effects of mitochondrial OMA1 deficiency on inhibiting OS tumorigenesis. Loss of OMA1 increases cytosolic GSK3β, which facilitates cytosolic β-catenin ubiquitination degradation, inhibiting OS cell proliferation and migration. OMA1 deletion also attenuates the interaction of cytosolic p53 with cytosolic Parkin, leading to excessive mitochondrial localization of Parkin and excessive mitophagy, thereby stimulating apoptosis. CPX suppresses cell growth and triggers apoptosis by promoting OMA1 self-cleavage and degrading L-OMA1, thereby inhibiting OS tumorigenesis.

There is an urgent need to identify new OS therapeutic targets due to the low survival rate and lack of effective treatments for metastatic OS patients. Here, we demonstrated the pro-tumorigenic activity of OMA1 in OS, at least in part, by regulating p53 mitochondrial transport and β-catenin nuclear translocation. Importantly, we found that CPX promotes OMA1 self-cleavage and degradation to suppress OS tumorigenesis (Fig. 7). Of note, osteosarcoma has multiple origins. Due to the rarity of osteosarcoma, we could only obtain samples from four patients with advanced OS. However, we observed similar phenotypes in all samples and confirmed our findings in OS cell lines and mice. Therefore, our findings reveal a new role for OMA1 in OS, deepen our understanding of the etiology of OS, and lay the foundation for the development of new therapies.

OMA1 senses mitochondrial stresses and is then activated to cleave L-OPA1, inhibiting mitochondrial fusion and then converting oxidative phosphorylation (OXPHOS) to glycolysis. Given that glycolysis, rather than OXPHOS, produces large amounts of ATP in cancer cells [39], aberrant expression of OMA1 in various types of cancer has been reported in The Cancer Genome Atlas (TCGA) database. For example, the level of OMA1 in some tumors, such as renal pheochromocytoma, clear cell carcinoma of the kidneys, pheochromocytoma, and paraganglioma, is lower than in normal tissues. Darverey et al. reported that low expression of OMA1 is associated with poor overall survival of patients with breast cancer. They found that OMA1 deficiency increases the proliferation and invasion of breast cancer cells [40]. On the other hand, the TCGA database shows that in certain tumors, such as gastric cancer, esophageal carcinoma, glioblastoma multiforme, squamous cell carcinoma of the cervix, and endocervical adenocarcinoma, the expression of OMA1 is relatively high in comparison with that in normal tissues. Consistently, OMA1 is upregulated under hypoxia and contributes to colorectal cancer development by promoting glycolysis, and OMA1 deficiency reduces colorectal cancer growth [15]. Additionally, OMA1 protects embryonic fibroblasts, HEK 293 cells, and C2C12 myoblasts from DNA damage. Lack of OMA1 promotes DNA damage-induced apoptosis in these cells [41]. Here, we also observed increased expression of OMA1 in advanced human OS samples and OMA1 deficiency promotes apoptosis and suppresses OS cell growth in vitro and in vivo. Jun et al. reported that PCGEM acts as a miR-433-3p sponge, blocking the inhibitory effect of miR-433-3p on OMA1 expression in OS cells [18]. Knockdown of PCGEM1 reduced OMA1 expression and inhibited cell proliferation and invasion, which was reversed by overexpression of OMA1, implicating the contribution of OMA1 to OS cell proliferation [18]. Therefore, OMA1 plays a complex role in tumorigenesis, depending on the type and stage of tumors, and OMA1 appears to promote tumorigenesis in OS.

It has been recently reported that OMA1 deficiency increases apoptosis in immortalized mouse embryonic fibroblasts, HEK293 cells, and C2C12 myoblasts treated with DNA damage inducers such as hydroxyurea, suggesting OMA1 protects cells from DNA damage-induced death [41]. Here, we observed elevated OMA1 expression in human OS samples. Stable knockout or transient knockdown of OMA1-induced apoptosis in human OS cell lines without additional stimuli. On the other hand, Jiang et al. used a Dox-inducible system to express B cell lymphoma-2 (Bcl-2) interacting mediator of cell death (Bim) and truncated BH3 interacting death domain agonist (tBid) in human osteosarcoma U2OS cells [8]. They found that Bim and tBid stabilized and activated OMA1, which cleaved L-OPA1 and resulted in cytochrome c (CytC) release and subsequent apoptosis [8]. Knockdown of OMA1 blocked Bim/tBid-induced apoptosis in U2OS, indicating that OMA1 is required for apoptosis induced by overexpression of Bim/tBid in U2OS [8]. Therefore, the protective effect of OMA1 deficiency on apoptosis depends on the cell type.

How does OMA1 deficiency promote apoptosis? We observed increased mitophagy in *OMA1* knockout cells. In general, the accumulation of dysfunctional mitochondria induces programmed cell death, such as apoptosis [42]. Therefore, appropriate mitophagy can eliminate damaged mitochondria promptly and is critical for maintaining healthy and functional mitochondria and preventing programmed cell death [43]. However, dysregulated mitophagy occurs in various pathological conditions, such as Parkinson’s disease and cancer. Defective mitophagy can induce apoptosis [43]. On the other hand, excessive mitophagy has also been reported to induce cell death. For example, Basit et al. reported that BAY 87-2243 induces mitophagy in melanoma cell lines, leading to cell death via necroptosis and ferroptosis [44]. Cigarette smoke exposure stabilizes PINK1 and causes excessive mitophagy and subsequent cell death in lung epithelial cells via mitophagy-dependent necroptosis [45]. Excessive mitophagy has also been reported to induce cell death in hepatocytes and neurons [46, 47]. Furthermore, Chen et al. reported that ketoconazole increases PINK1 and Parkin and stimulates excessive mitophagy, thereby inducing apoptosis in hepatocellular carcinoma [19]. Consistently, in the current study, we observed that OMA1 deficiency increases PINK1 and Parkin levels and subsequently leads to excessive mitophagy and apoptosis. Therefore, excessive mitophagy induced by OMA1 inhibition appears to cause apoptosis in the OS cell lines we tested.

PINK1 and Parkin play an important role in inducing mitophagy [48, 49]. In healthy mitochondria, PINK1 is constitutively and rapidly degraded by the inner mitochondrial membrane (IMM)-bound proteases and is maintained at low levels. Under stress conditions, such as mitochondrial damage and unbalanced mitochondria membrane potential, PINK1 accumulates on the OMM and is activated, recruiting and activating Parkin, which ubiquitinates mitochondrial substrates, such as voltage-dependent anion channel 1 (VDAC1) [50]. This modification recruits the autophagy adaptor molecule, p62/SQSTM1, which targets mitochondria for autophagic removal [50]. Emerging evidence shows that p53 regulates PINK1/Parkin-mediated mitophagy. Cytosolic p53 in hepatocytes binds to Parkin and prevents its transfer from the cytosol to mitochondria, thereby inhibiting mitophagy in the livers of mice with heatstroke-induced acute liver injury or metabolically stressed ob/ob mice [51, 52]. In aged or doxorubicin-treated mouse hearts, cytosolic p53 also attenuates mitophagy through binding to Parkin, promoting cardiotoxicity [20]. In addition, Zhang et al. reported that p53 inhibits mitophagy in bone marrow mesenchymal stem cells under oxidative stress by binding to cytosolic Parkin and blocking its mitochondrial translocation [53]. Furthermore, mitochondrial p53 can promote apoptosis by directly interacting with Bcl-2 family proteins to release CytC in a transcription-independent manner [54–56]. Diverse stimuli, such as stress and mitochondrial uncoupling, can stimulate mitochondrial localization of p53 to trigger apoptosis [56, 57]; however, the molecular mechanism regulating p53 mitochondrial translocation remains elusive. Here, we found that OMA1 deficiency reduces the interaction of cytosolic p53 with cytosolic Parkin, which may lead to excessive mitochondrial localization of Parkin and mitophagy, thereby stimulating apoptosis. We also observed that mitochondrial p53 levels are increased in *OMA1-deficient* OS cells, although the underlying mechanisms are unclear. Considering that mitochondrial p53 promotes the release of pro-apoptotic factors from the mitochondrial intermembrane space [20, 21]. Therefore, OMA1 deficiency may promote mitochondrial p53 to directly drive apoptosis. Alternatively, both mechanisms may contribute to the enhanced apoptosis observed in *OMA1-deficient* OS cells.

The mutation rate of p53 in osteosarcoma is high, ranging from 20% to 90%. These mutations may lead to loss of or gain of function, thereby affecting tumorigenesis [58]. p53 is highly expressed in SJSA-1 and 143B cells, as evidenced by IHC and RT-qPCR. Standard genomic DNA and cDNA sequencing unveiled that SJSA-1 holds wild-type p53, while 143B harbors mutant R156P p53 [59]. R156P localizes within the DNA-binding domain of p53 and impairs its binding to DNA, reducing the transcriptional activity of p53 [60]. Additionally, Luo et al. reported that R156P may affect the secondary structure of p53 and impair the rigidity-sensing function of OS cells [61]. Whether R156P affects the subcellular localization of p53 is unknown. However, we observed similar phenotypes in SJSA-1 and 143B cells, indicating that R156P is not the primary driver of changes caused by OMA1 deficiency.

The canonical Wnt/β-catenin pathway plays a critical role in promoting tumor development and progression, including OS. The accumulation of β-catenin, a key downstream regulatory component of Wnt signaling, in the cytosol enhances its translocation to the nucleus, where the protein induces the expression of target genes [62]. β-catenin expression is increased in human OS samples, and its overexpression is positively associated with tumor size, distant metastasis, and poor survival in patients with OS [63–65]. Inhibition of the Wnt/β-catenin activity reduces OS cell proliferation, migration, and colony formation [66]. We observed that knockout of OMA1 significantly reduces β-catenin in the cytoplasm and nucleus of OS cells. β-catenin agonist reverses the inhibitory effect of OMA1 deficiency on OS cell migration and colony formation, suggesting that β-catenin plays an important role in these processes.

How does OMA1 deficiency lead to reduced cellular β-catenin levels? It is well known that GSK3β, as a negative regulator of the canonical Wnt/β-catenin signaling, can form a complex with cytosolic β-catenin to phosphorylate the protein, leading to proteasomal degradation of β-catenin. Dissociation of β-catenin from GSK3β is essential for its nuclear translocation [62, 67]. Indeed, we observed accumulation of cytosolic GSK3β and increased interaction of GSK3β and β-catenin and the ubiquitination of β-catenin in *OMA1-deficient* OS cells, which may be responsible for the reduction in β-catenin caused by *OMA1* knockout. In addition, phosphorylation of GSK3β on Ser9 leads to proteasomal degradation and inactivation of GSK3β [68], and ROS can increase Ser-9 phosphorylation of GSK3β [27–29]. Knockout of OMA1 in OS cells significantly reduced mitochondrial ROS levels in both 143B and SJSA-1 cells, which may contribute to the decrease in phosphorylated GSK3β-Ser9 levels and, the subsequent increase in total GSK3β levels. Excessive mitophagy induced by *OMA1* knockout may be attributed to reduced mitochondrial ROS production. However, the detailed mechanism by which OMA1 deficiency leads to cytoplasmic GSK3β accumulation requires further investigation.

CPX has been shown to exert tumor suppressive effects in various cancers, such as lung adenocarcinoma and gastric cancer, via different mechanisms. CPX suppresses cell proliferation and induces apoptosis by inhibiting topoisomerase II alpha in lung adenocarcinoma cells and reducing DJ-1 in colorectal cancer cells [69, 70]. In gastric cancer, CPX has been reported to reduce signal transducer and activator of transcription 3 (STAT3) phosphorylation, leading to cell growth arrest and autophagic death [35]. CPX, together with bortezomib, promotes apoptosis and induces cellular senescence in glioblastoma multiforme cells [71]. Here, we observed that CPX reduces OMA1 expression, suppresses cell proliferation, and promotes apoptosis, thereby inhibiting OS tumorigenesis through a mechanism involving OMA1. We observed that CPX stimulates OMA1 degradation via the polyubiquitination-proteasome pathway. Consistently, Yang et al. reported that lectin induces OMA1 degradation in mesenchymal stem cells primarily via the polyubiquitination-proteasome pathway [37]. Wang et al. also reported that knockdown of p66Shc increased OMA1 ubiquitination and reduced OMA1 protein levels in hepatocytes [36]. Furthermore, OMA1 interacts with the 26S proteasome [6]. Of note, OMA1 is a mitochondrial inner membrane protein. It is currently unclear how OMA1 is degraded by the polyubiquitination-proteasome pathway that occurs in the cytosol. OMA1 is a nuclear-encoded protein containing a mitochondrial import sequence at the N-terminus [6]. It is possible that OMA1 is polyubiquitinated and then delivered to the proteasome for degradation during its transport to mitochondria. Alternatively, polyubiquitination-proteasomal degradation may occur after OMA1 reaches the inner mitochondrial membrane, but how this occurs is unclear. Recently, Sun et al. reported that the ubiquitin-proteasome system can degrade photosynthesis proteins inside intact double membrane-bound chloroplasts [72]. It would be of interest to evaluate whether a similar mechanism exists in mammalian cells. Nevertheless, the antitumor effects of CPX indicate its therapeutic potential in cancer treatment.

In summary, we elucidated the critical role of OMA1 in regulating OS tumorigenesis and revealed that CPX promotes OMA1 self-cleavage and induces L-OMA1 degradation via ubiquitination and lysosome pathways. Mechanistically, OMA1 deficiency promotes p53-mediated programmed death response. OMA1 deletion also increases cytoplasmic GSK3β, which promotes β-catenin degradation and impedes β-catenin nuclear translocation, rendering OS cells a growth arrest (Fig. 7). Therefore, our findings indicate the promise and potential of OMA1 as a valuable therapeutic target for OS.

## Materials and methods

### Ethics statement

All human samples were obtained from patients at the Affiliated Qingyuan Hospital of Guangzhou Medical University. The study of these samples was approved by the Ethics Committee of Qingyuan Hospital (Approval No.IRB-2022-081), and standard informed consent and consent for publication of the images were obtained from the patients. All research was conducted in accordance with both the Declarations of Helsinki and Istanbul. Animal experiments were performed in accordance with the Institutional Animal Ethics Committee guidelines for the Use of Experimental Animals at the Affiliated Qingyuan Hospital of Guangzhou Medical University, Qingyuan People’s Hospital (Approval No. LAEC-2023-022).

### Patient specimens

The OS tissue specimens, including adjacent non-cancerous and tumor tissues from the same sample, were collected from the Affiliated Qingyuan Hospital of Guangzhou Medical University. All patients were clinically and pathologically confirmed to have OS. After surgical resection, tissues were immediately frozen in liquid nitrogen and stored at −80 °C until use.

### Animal experiments

Male nude mice were housed under specific pathogen-free conditions. Control and *OMA1* KO 143B cells (1 × 10^7^) per mouse, 100 µl supplement with 100 µl of Matrigel® Matrix Basement Membrane HC (BD Biocoat, Corning, New York, USA) were injected subcutaneously into the right armpit of 4-week-old Balb/c nude mice (*n* = 4). Tumor growth was monitored every 2 days by measuring the length and width of the tumor, and tumor volume was calculated by the following formula: volume = (length × width^2^)/2. After 12 days, the mice were euthanized and photographed, and tumors were dissected, weighed, and fixed.

The cell-line-derived OS tumor xenograft model for in situ in nude mice was constructed as follows: Control, *OMA1* KO, and re-expressed *OMA1*-Flag or *OMA1*-E324Q-Flag in *OMA1-deficient* 143B cells (6 × 10^5^ cells per mouse) were prepared into a single-cell suspension and injected subcutaneously into the proximal right tibia of 4-week-old Balb/c nude mice (*n* = 5). After the indicated time, mice were anesthetized with isoflurane and subjected to MRI. The image of the OCOR T2 Prop fs series was acquired using ITK-SNAP software, and the tumor volume was calculated using software 2Dslicer.

### Cell culture, reagents, and antibodies

Human 143B, SJSA-1, and HEK 293T cells were generously provided by Dr. Xiangjiang Wang (The Affiliated Qingyuan Hospital of Guangzhou Medical University) and cultured in Dulbecco’s modified Eagle’s medium (DMEM, Gibco, Thermo Fisher Scientific, MA, USA) supplemented with 10% fetal bovine serum (FBS, Newzerum, Christchurch, New Zealand) and 1% penicillin-streptomycin (Gibco, Thermo Fisher Scientific, MA, USA) at 37 °C in a humidified incubator with 5% CO_2_. Cell lines were authenticated by short tandem repeats (STR) and routinely checked and confirmed to be free of mycoplasma.

The reagents and kits used were as follows: The Pierce BCA^TM^ protein assay kit, MTT Cell Proliferation and Cytotoxicity Assay Kit, Cell cycle Analysis Kit, BeyoClick EdU-488 Cell Proliferation Detection Kit, Crystal Violet Staining Solution, Horseradish peroxidase (HRP)-conjugated secondary antibodies (Goat anti-rabbit and goat anti-mouse), Alexa Fluor Plus 555, and 4′,6-diamidino-2-phenylindole (DAPI) were purchased from the Beyotime Institute of Biotechnology (Haimen, Jiangsu, China). The FITC Annexin V Apoptosis Detection Kit I was from BD Pharmingen^TM^ (Franklin Lakes, New Jersey, USA). The MitoSOX Red, 3-MA, BafA1, MG132, RAPA, CQ, CPX, and β-catenin agonists were purchased from MedChemExpress (New Jersey, USA). The MitoTracker® Deep Red FM and MitoTracker® Green FM were obtained from Cell Signaling Technology (Beverley, MA, USA). The LysoTracker™ Deep Red was from Thermo Fisher Scientific Inc (Invitrogen, MA, USA).

Primary antibodies were: anti-p62 (ab56416), anti-LC3 (ab192890), anti-caspase-3 (ab13847), anti-Fibronectin (ab268020), anti-vimentin (ab92547), and anti-OMA1 (ab154949) from Abcam (Cambridge, UK); Anti-p21 (10355-1-AP), anti-CyclinD1 (26939-1-AP), anti-Rb (17218-1-AP), β-catenin (51067-2-AP), anti-LaminB1 (12987-1-AP), anti-HA tag (51064-2-AP), anti-Flag tag (66008-4-Ig), and anti-GFP tag (66002-1-Ig) were purchased from ProteinTech (Wuhan, Hubei, China). Anti-ubiquitin (sc-8017) and anti-OMA1 (sc515788) were purchased from Santa Cruz Biotechnology (Dallas, TX, USA). Anti-p-Rb (Ser807/811) (8516T), anti-p53 (9282), anti-PINK1 (6946S), anti-Parkin (4211), anti-Parkin (2132) anti-GAPDH (2118), anti-GSK3β (9832S), anti-p-GSK3β (9323S) anti-Ki67 (9449S), and anti-LAMP1 (9091) were purchased from Cell Signaling Technology (Beverley, MA, USA). Anti-CDK4 (R23889) and anti-β-tubulin (380628) were purchased from Zen Bioscience (Chengdu, Sichuan, China). Anti-PARP1 (A19596) and anti-E-cadherin (A22850) were purchased from ABclonal (Wuhan, Hubei, China). Anti-β-tubulin (M20005M) was purchased from Abmart (Shanghai, China). Anti-OPA1(18/OPA1) (612607) was obtained from BD PharmingenTM (Franklin Lakes, New Jersey, USA).

### Constructing knockout cell lines

143B and SJSA-1 cells lacking OMA1 were generated by CRISPR/Cas9 gene editing. The sgRNA sequence of OMA1 was selected as described previously [15] and cloned in eSpCas9-LentiCRISPR v2 plasmid (Addgene 52961) by CloneEZ. Lentiviruses were prepared by transfecting HEK 293T cells (2 µg plasmid/60 mm dish) using PEI (Maokang Biotechnology, Shanghai, China). The cDNAs of *OMA1*-Flag and its dominant negative mutant *OMA1*-E324Q-Flag were purchased from Tsingke Biotechnology Co., Ltd (Beijing, China). 143B and SJSA-1 cells were infected with the lentiviruses for 48 h and selected with 0.5 and 1.0 µg/ml puromycin (Amresco, Shanghai, China) for 5–7 days to prepare stable transformants. Single cells were then sorted into 96-well dishes, and viable clones were expanded and checked by RT-qPCR and Western blotting.

### Cell viability and proliferation, cell cycle distribution, and apoptosis analysis

Cell viability, cell proliferation, cell cycle distribution, and apoptosis analysis were performed as described previously [35]. All analyses were performed in triplicate at least 3 times, with at least 10,000 cells examined per condition.

### Western blotting, immunohistochemistry, and RNA extraction, and RT-qPCR

Western blotting, immunohistochemistry, RNA extraction, and RT-qPCR were performed as described previously [35]. Primers used were: Human *OMA1*: forward, 5′-TACTTCTCCACGGTTTCAA GC-3′, and reverse 5′-GATTGGACTTACTTCCAGGTGAG5′; Human *β-ACTIN*: forward, 5’-AGCACAGAGCCTCGCCTTTG, and reverse 5′-AAGCCGGCCTTGCACATG-3′.

### Immunofluorescence staining

143B and SJSA-1 cells were grown on microscope cover glasses in a 24-well plate, incubated with 250 nM MitoTracker or/and 500 nM LysoTracker for 30 min at 37 °C, fixed in ice-cold 100% methanol at –20 °C for 15 min, and rinsed 3 times with 1× PBS for 5 min. After appropriate treatment, cells were fixed with 4% paraformaldehyde for 15 min at room temperature and washed with 1× PBS. Cells were then permeabilized with 0.3% Triton X-100 in PBS for 30 min at room temperature, blocked with blocking buffer (5% BSA and 0.3% Triton-X in PBS) for 2 h at room temperature, and incubated with anti-GSK3β (1:100), anti-β-catenin (1:100), or β-tubulin (Zen Bioscience, 1:100; Abmart, 1:1000) overnight at 4 °C. After washing with 0.3% Triton-X in PBS, cells were incubated with Alexa Fluor Plus 488 for 1 h at room temperature. Cells were stained with DAPI before mounting and imaging on an LSM900 Zeiss laser confocal microscope (Zeiss, German).

### Immunoprecipitation and ubiquitination assay

Cells were lysed with 1× Co-IP cell lysis buffer (Cell Signaling Technology, Beverley, MA, USA) supplemented with protease inhibitors for 30 min on ice. Cell lysates were centrifuged at 12,000 rpm for 30 min at 4 °C. The concentration of the supernatant was determined by BCA assay using the Pierce^TM^ BCA Protein Assay Kit. Cell lysate was cleared using Protein A/G PLUS-Agarose (30 µl/1 mg supernatant, Santa Cruz Biotechnology, Dallas, TX, USA). The mixture was rotated at 4 °C for 4 h. After centrifugation at 12,000 rpm for 5 min, the supernatant was collected and incubated with either anti-β-catenin (2 μg) or p53 (1:50) antibody overnight at 4 °C. 50 µl Protein A/G PLUS-Agarose was then added to each sample. After rotating at 4 °C for 4 h, the samples were centrifuged at 12,000 rpm for 5 min. The pellets were washed three times with 1× Co-IP cell lysis buffer. Immunoprecipitated proteins were eluted in 2× SDS-Loading buffer and analyzed using Western blotting.

### Preparation of cytoplasm, nuclear extracts, and mitochondria

The preparation of nuclear extracts and mitochondria was performed as described previously [73]. Cells were collected from two 100 mm culture dishes and treated with 1000 µl ice-cold IB_cells_-1 solution. All glassware was prechilled in an ice bath for 5 min before being homogenized. 300 ul of the homogenate was centrifuged at 14,000 × *g* for 30 min at 4 °C. The supernatant was collected as the cytosolic fraction and stored on ice. 700 µl cell suspensions were homogenized in a glass homogenizer (150–200 times/sample) and then centrifuged at 600 × *g* for 5 min at 4 °C. The supernatant was collected and centrifuged at 800 × *g* at 4 °C for 5 min. The pellets were the crude nuclei, and the supernatant was collected and centrifuged at 7000 × *g* for 10 min. The pellets were then resuspended in 1 ml of ice-cold IB_cells_-2 and centrifuged at 10,000 × *g* for 10 min. The supernatant containing mitochondria was collected. The crude nuclei were treated with 1 ml of 0.25 mol/L sucrose citric acid solution, then slowly added to another fresh tube containing 4 ml of 0.88 mol/L sucrose citric acid solution, and centrifuged at 3000 × *g* for 10 min at 4 °C. The pellets were washed twice with 0.05 mol/L Tris-HCl-NaCl solution and centrifuged at 2000 × *g* for 10 min at 4 °C. Total proteins in cytosolic fraction, nuclear fraction, and mitochondrial fraction were extracted using Triton X-100 cell lysis buffer containing phosphate inhibitor cocktail for 20 min on ice. Protein concentration was determined using the BCA assay.

### Statistical analysis

Data were expressed as mean ± SD. All experiments were repeated at least three times (biological replicates). Statistical significance was assessed with One-way ANOVA with Bonferroni post-test or two-tailed unpaired Student’s *t*-test, using GraphPad Prism 8.0 software (GraphPad Software Inc., San Diego, CA, USA). *P* < 0.05 was considered statistically significant (*P < 0.05, **P < 0.01, ***P < 0.001, ****P < 0.0001, and n.s., no significant difference). The Kaplan–Meier survival method was used to analyze the relationship between OMA1 expression and the prognosis of patients with OS. The correlation between OMA1 and β-catenin or p53 was calculated by Liner regression (95% confidence interval) using GraphPad Prism 8.0 software.

## Supplementary information

Supplementary Material

Original Data

## Data Availability

Data is contained within the article or supplementary material.
