# Supplementary material for: Inhibition of mitochondrial OMA1 ameliorates osteosarcoma tumorigenesis
Source: Cell Death Dis. 2024 Nov 1;15(11):786. doi: 10.1038/s41419-024-07127-1 (PMC11530700; doi:10.1038/s41419-024-07127-1)
Supplement: Supplementary file 1 — Supplementary Material [file 41419_2024_7127_MOESM1_ESM.docx]

**Fig.S1**


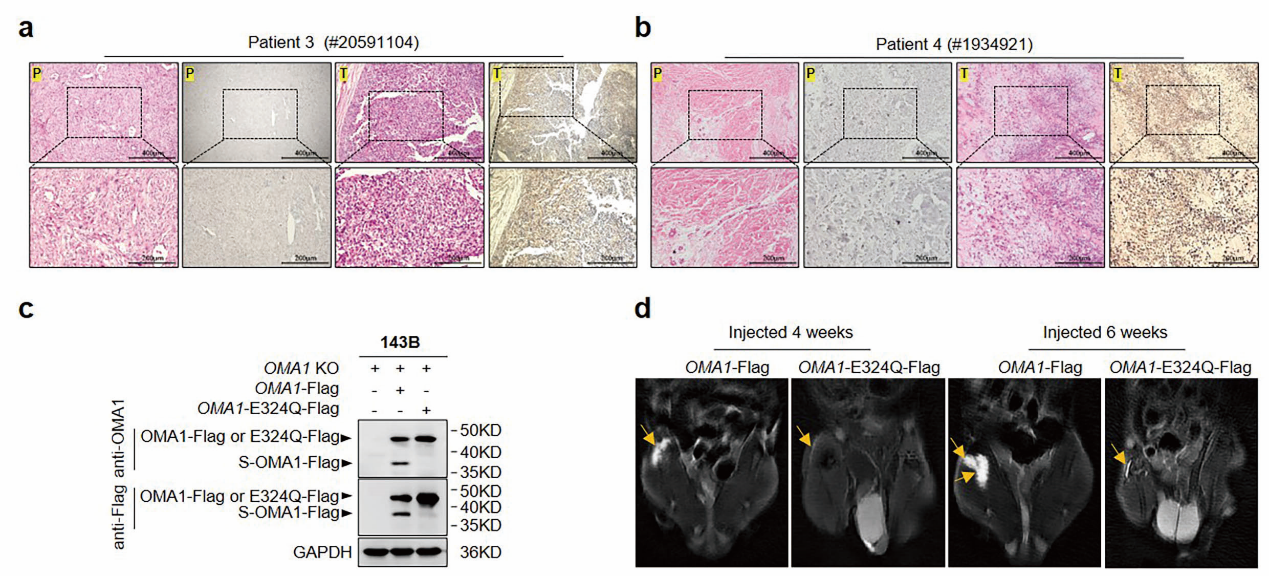
**Fig.S1 The catalytical activity of OMA1 is critical for OS tumorigenesis. a, b** H&E of OS tumors (T) and para-cancerous (P) tissues from patient 3 (#20591104) and patient 4 (#1934921) with advanced OS. The OMA1 levels in OS tissues from tumors and adjacent tissues were analyzed by IHC staining analysis (Scale bars, 400 µm; 200 µm). **c** The effectiveness of re-expressing *OMA1*-Flag and catalytic mutant *OMA1*-E324Q-Flag in *OMA1* KO 143B cells was measured using Western blotting analysis. **d** Representative MRI images of nude mice with right proximal tibia injected with the re-expressing *OMA1-*Flag and *OMA1*-E324Q-Flag of *OMA1* KO 143B cells for 4 or 6 weeks (n=5). The arrowhead indicates the location of bone lesions and tumors.

**Fig.S2**


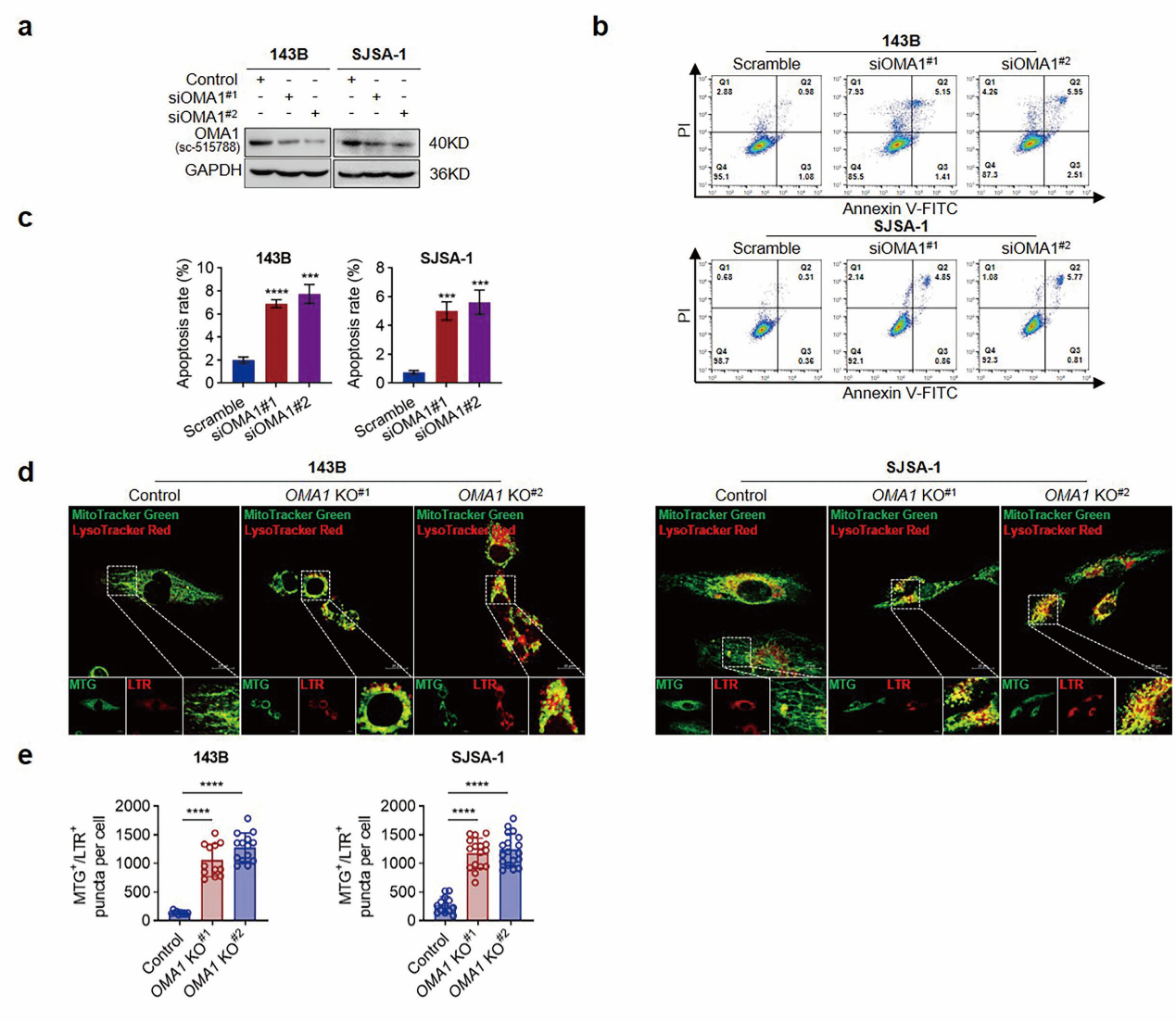
**Fig.S2 OMA1 deficiency induces apoptosis and mitophagy of OS cells.** **a** Western blotting analysis of OMA1 in OS cells transfected with siOMA1 for 48 h. **b, c** 143B and SJSA-1 were stained with annexin V-FITC/PI using flow cytometry after transfecting with siOMA1 for 48 h (**b**). The apoptosis rates were plotted followed by statistical analysis (**c**). Data were shown as mean ± SD, and statistical significance was assessed by One-way ANOVA with Bonferroni post-test and two-tailed unpaired Student’s t-test (n=3, ****P* < 0.001, *****P* < 0.0001). **d, e** Representative confocal microscopy images of the co-localization of MitoTracker Green (MTG) and LysoTracker Red (LTR) in control and *OMA1-deficient* cells (**d**) (Scale bar, 20 µm). The MTG^+^/LTR^+^ puncta per cell was quantified using ImageJ Plus software (**e**). Data were shown as mean ± SD and statistical significance was assessed by One-way ANOVA with Bonferroni post-test and two-tailed unpaired Student’s *t*-test (n＞3, *****P* < 0.0001).

**Fig.S3**


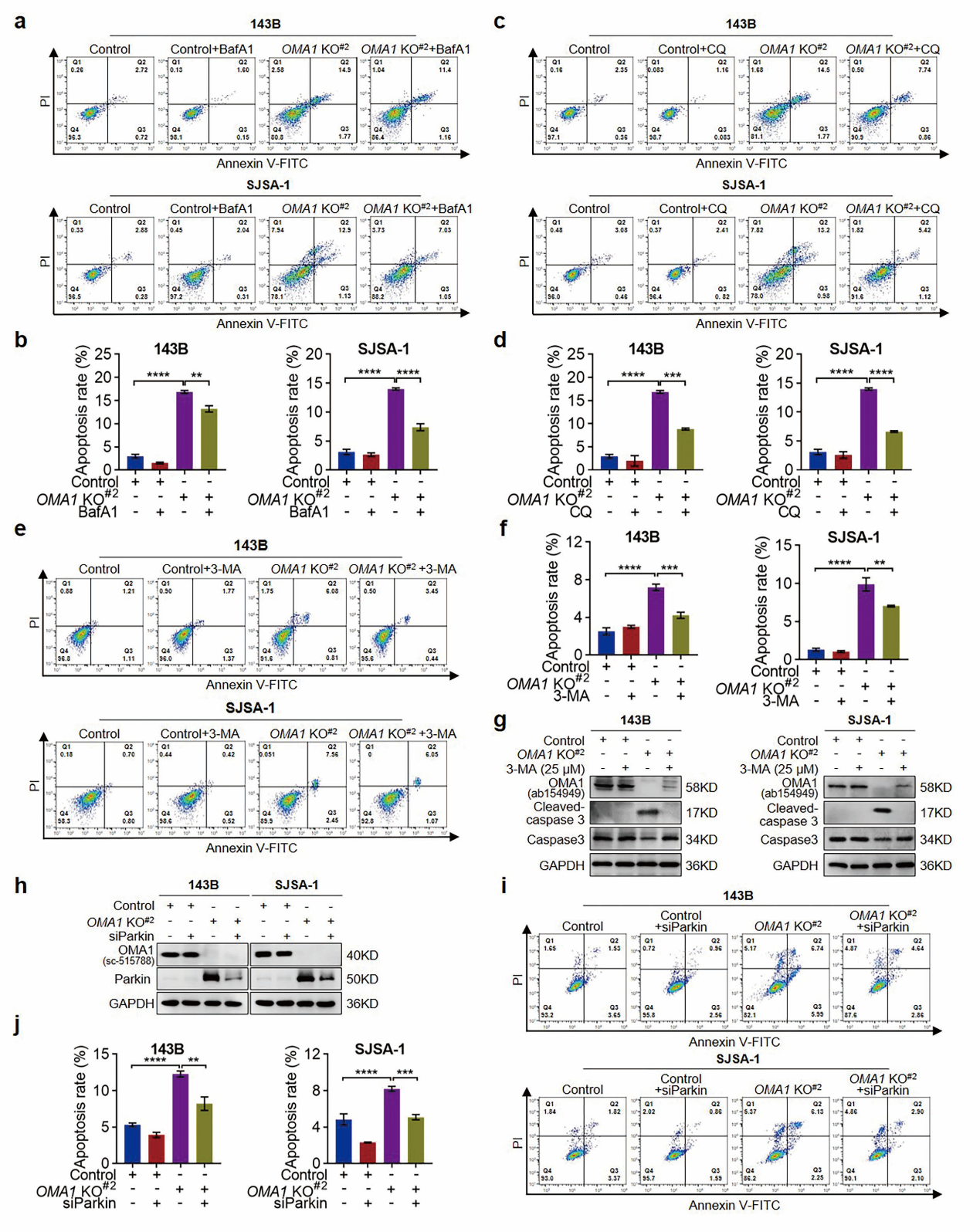
**Fig.S3 OMA1 deficiency induces apoptosis via excessive mitophagy activation in OS cells.** **a-f** Apoptosis of control or *OMA1-deficient* cells treated with BafA1 (50 nM) (**a**), CQ (100 µM) (**c**), or 3-MA (25 µM) (**e**) for 24 h and stained with Annexin V-FITC/PI were analyzed using flow cytometry. The apoptosis rates were plotted followed by statistical analysis (**b, d, f**). Data were shown as mean ± SD, and statistical significance was assessed by two-tailed unpaired Student’s *t*-test (n=3, ***P* < 0.01, ****P* < 0.001, *****P* < 0.0001). **g** Western blotting analysis of Cleaved-caspase 3 and Caspase 3 protein levels in *OMA1-deficient* cells treated with 3-MA (25 µM) for 24 h. **h** Control and *OMA1-deficiency* were transfected with siParkin for 48 h and cell lysates were assessed for the OMA1 and Parkin by Western blotting analysis. **i, j** Apoptosis of control or *OMA1-deficiency* cells transfected with siParkin for 48 h and stained with Annexin V-FITC/PI were analyzed using flow cytometry (**i**). The apoptosis rates were plotted followed by statistical analysis (**j**). Data were shown as mean ± SD, and statistical significance was assessed by two-tailed unpaired Student’s t-test (n=3, ***P* < 0.01, ****P* < 0.001, *****P* < 0.0001).


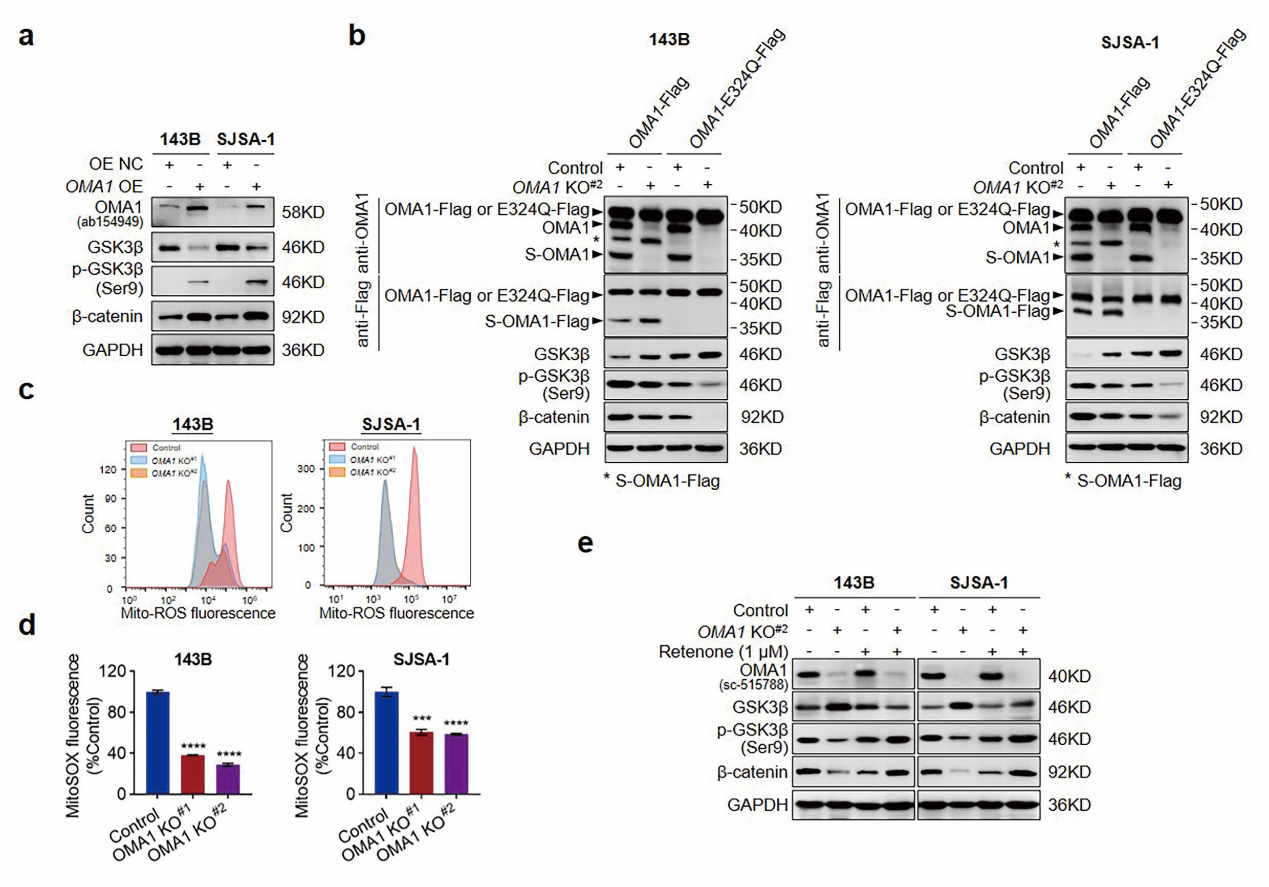
**Fig.S4**

**Fig. S4 OMA1 activity and mitochondrial ROS level are essential for GSK3β/β-catenin signaling in OS cells. a** The whole cell lysates of *OMA1-overexpressing* cells were determined by Western blotting with antibodies against GSK3β, p-GSK3β, or β-catenin. **b** Lysates from control and *OMA1-deficiency* 143B and SJSA-1 cells expressing mouse *OMA1*-Flag or *OMA1*-E324Q-Flag were assessed for the processing of OMA1 and the protein levels of GSK3β, p-GSK3β, and β-catenin by Western blotting analysis. **c, d** Mitochondrial ROS in *OMA1* knockout cells stained with MitoSOX was detected using flow cytometry (**c**). The MitoSOX fluorescence ratio was plotted using the mean ± FITC value (**d**). Data were shown as mean ± SD, and statistical significance was assessed by One-way ANOVA with Bonferroni post-test and two-tailed unpaired Student’s t-test (n=3, ****P* < 0.001, *****P* < 0.0001). **e** Western blotting analysis of GSK3β, p-GSK3β, and β-catenin protein levels in *OMA1-deficiency* cells treated with Retenone (1 µM) for 48 h.

**Fig.S5**
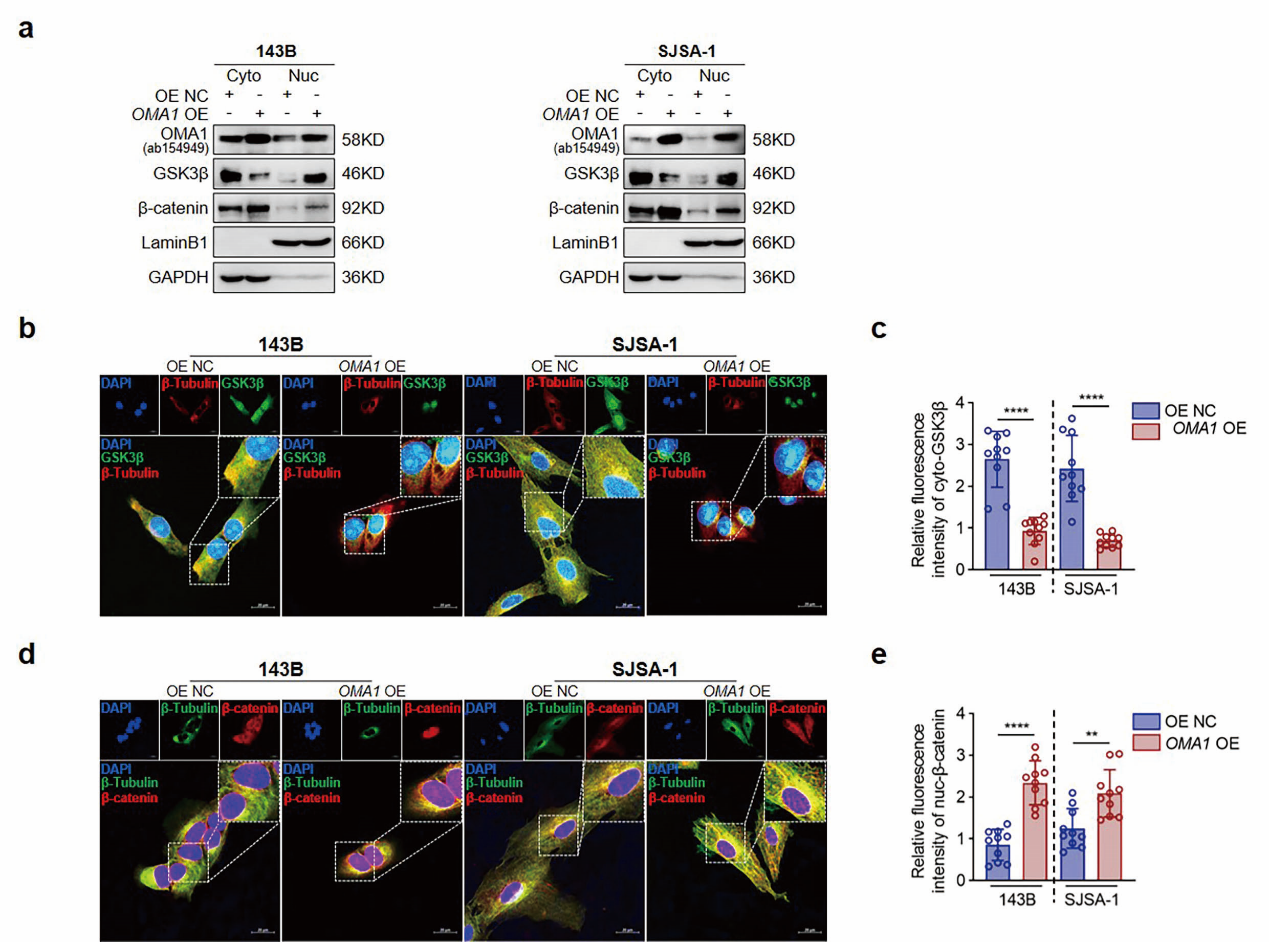


**Fig. S5 Overexpression of OMA1 decreased cytosolic GSK3β and increased β-catenin nuclear transportation. a** The protein levels of GSK3β, and β-catenin in the cytoplasm (Cyto) and nucleus (Nuc) of *OMA1-overexpressing* OS cells were analyzed by nuclear/cytosolic fractionation and Western blotting. **b-e** Representative confocal microscopy images of the co-localization of β-tubulin and cytosolic GSK3β (**b**) or nuclear β-catenin (**d**) in *OMA1-deficient* cells (Scale bar, 20 µm). The relative fluorescence intensity of cytosolic GSK3β (**c**) or nuclear β-catenin (**e**) was quantified using ImageJ Plus software. Data were shown as mean ± SD and statistical significance was assessed by two-tailed unpaired Student’s *t*-test (n＞3, ***P* < 0.01, *****P* < 0.0001).

**Fig.S6**
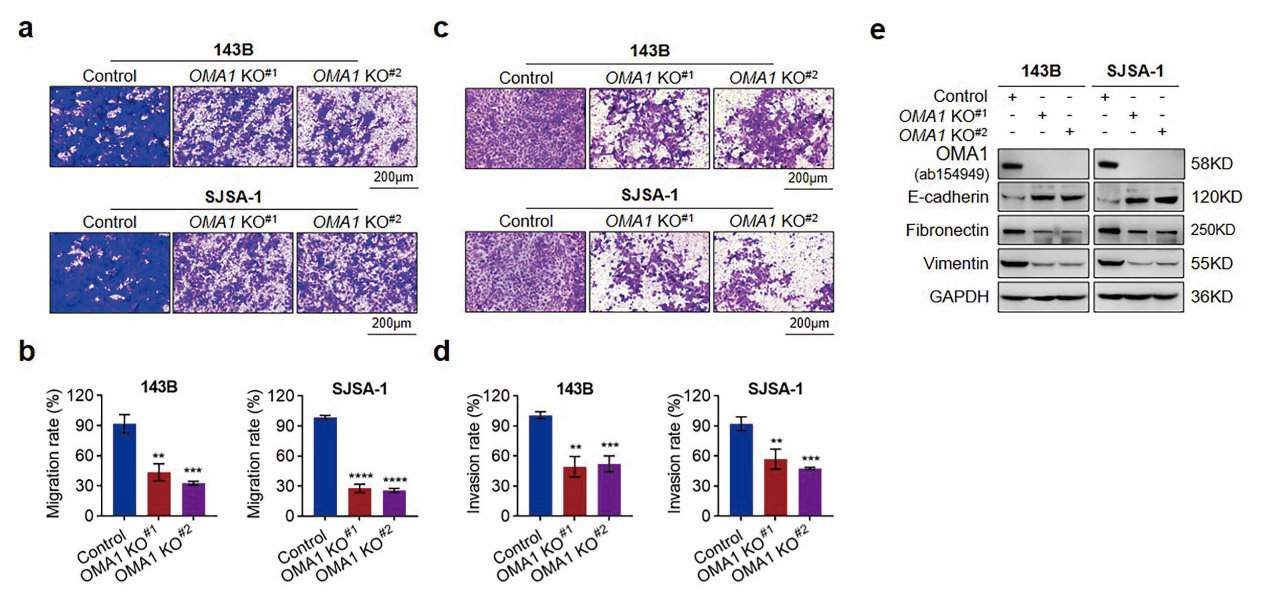


**Fig. S6 OMA1 deficiency decelerates migration and invasion of OS cells. a, b** Cell migration activity of control and *OMA1-deficient* cells were detected using Transwell migration assay (**a**), and quantification of cell migration rate was determined using ImageJ Plus software (**b**). Migrated cells were stained with 0.1 % crystal violet solution and then imaged with a light microscope (Scale bar, 200 µm). Data were shown as mean ± SD, and One-way ANOVA assessed statistical significance with Bonferroni post-test and two-tailed unpaired Student’s *t*-test (n=3, ***P* < 0.01, ****P* < 0.001, *****P* < 0.0001). **c, d** Cell invasion activity of control and *OMA1-deficient* cells was detected using Transwell invasion assay (**c**), and quantification of cell invasion rate was determined using ImageJ Plus software (**d**). Invaded cells were stained with 0.1 % crystal violet solution and imaged with a light microscope (Scale bar, 200 µm). Data were shown as mean ± SD, and One-way ANOVA assessed statistical significance with Bonferroni post-test and two-tailed unpaired Student’s *t*-test (n=3, ***P* < 0.01, ****P* < 0.001). **e** The levels of cell migration- and invasion-related proteins were detected using Western blotting analysis.

**Fig.S7**
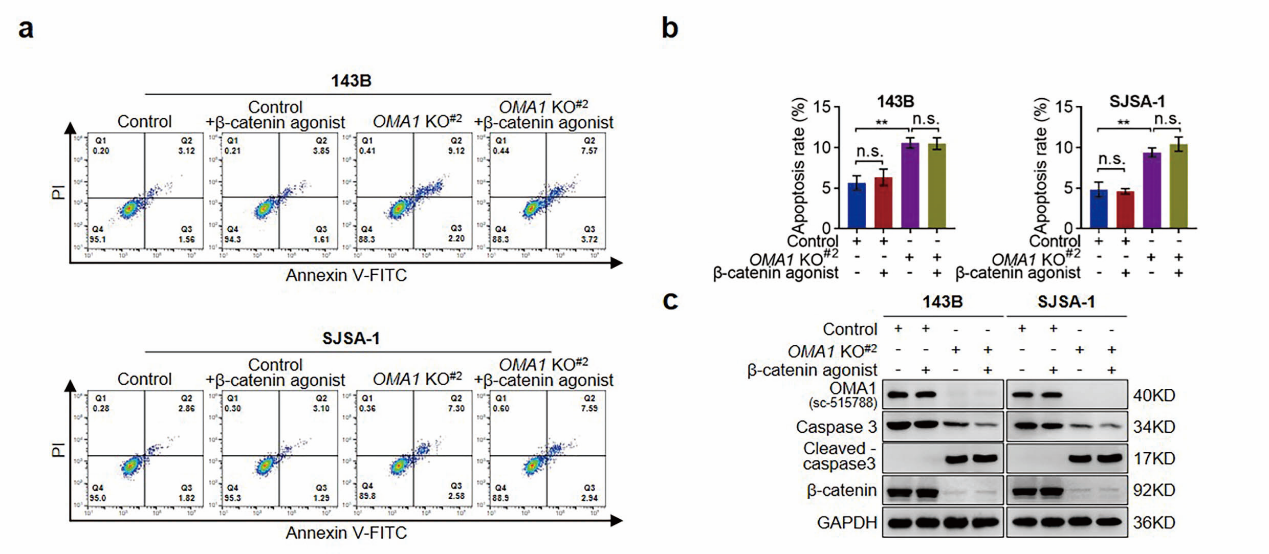


**Fig. S7 The role of β-catenin on *OMA1* deficiency-induced apoptosis in OS cells is negligible. a, b** Control and *OMA1-deficiency* 143B and SJSA-1 cells following treatment with β-catenin agonist (10 µM) for 12 h were determined using flow cytometry (**a**). The apoptosis rates were plotted followed by statistical analysis (**b**). Data were shown as mean ± SD, statistical significance was analyzed by two-tailed unpaired Student’s t-test (n=3, ***P*<0.01, or n.s., not significant). **c** Western blotting analysis of apoptotic-related protein in *OMA1-deficiency* cells treated with β-catenin agonist (10 µM) for 12 h.


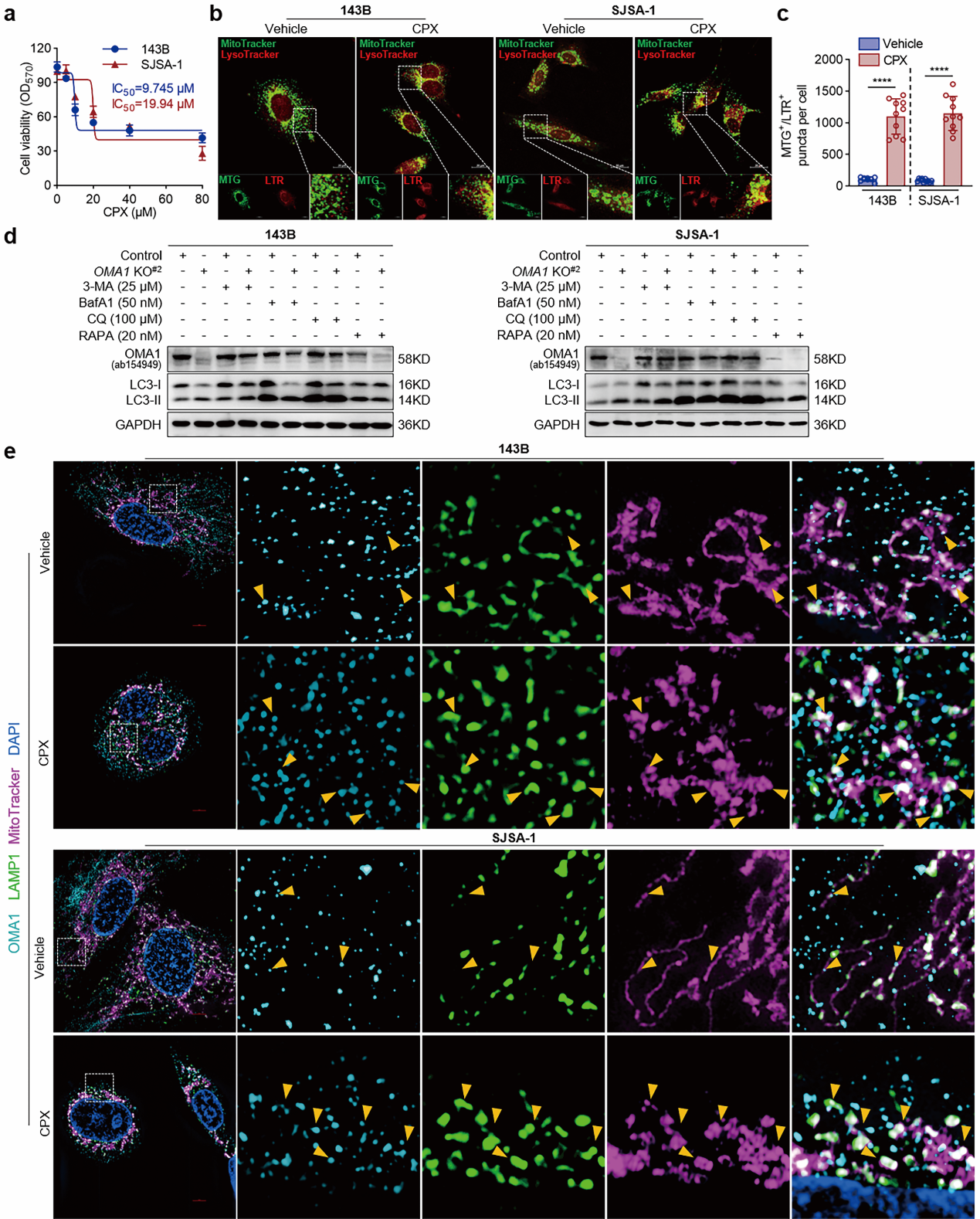
**Fig.S8**

**Fig.S8 CPX promotes OMA1 protein degradation via the ubiquitination and lysosome pathways in OS cells. a** The IC_50_ value of OS cells treated with a serial dose of CPX for 24 h was evaluated using an MTT cellular proliferation and cytotoxicity assay kit. Data were shown as mean ± SD (n=6). **b, c** Representative confocal microscopy images of the co-localization of MitoTracker Green (MTG) and LysoTracker Red (LTR) in OS cells treated with CPX (10 µM) for 24 h (**b**) (Scale bar, 20 µm). The MTG^+^/LTR^+^ puncta per cell was quantified using ImageJ Plus software (**c**). Data were shown as mean ± SD and statistical significance was assessed by two-tailed unpaired Student’s *t*-test (n＞3, *****P* < 0.0001). **d** Western blotting analysis of OMA1 protein levels in control and *OMA1-deficient* cells treated with an indicated concentration of three autophagy inhibitors or activator RAPA for 24 h. **e** Representative images of OS cells treated with 10 µM CPX for 24 h before immunostaining with MitoTracker Green (MTG), LAMP1 (1:200), and endogenous OMA1 (1:200). Cells were stained with DAPI before mounting and imaging on a Nikon A1R-SIM-STORM (Nikon, Japan) (Scale bar, 5 µm).


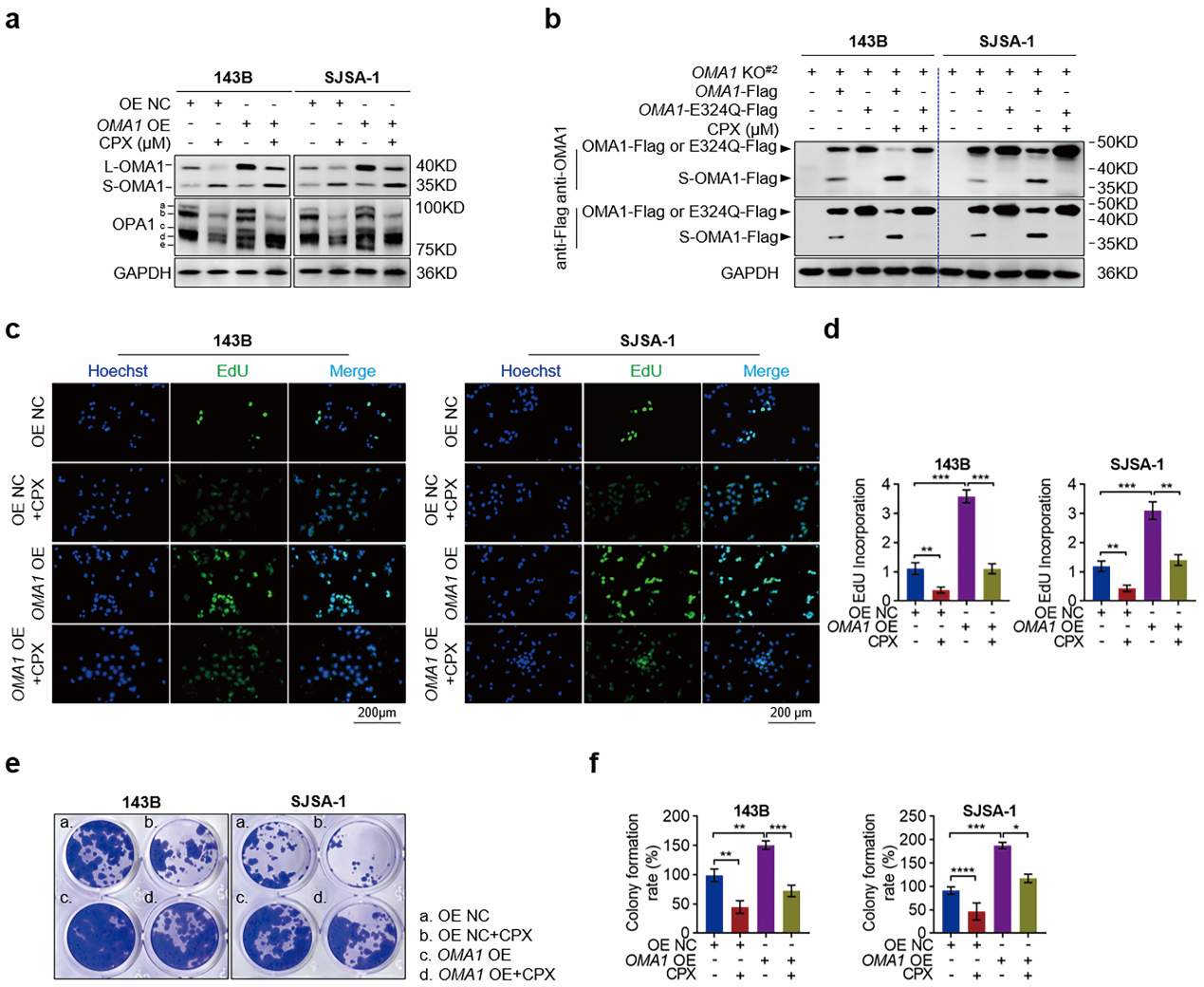
**Fig.S9**

**Fig.S9 CPX inhibits OS cell proliferation through OMA1. a** Control and *OMA1-overexpressing* cells were incubated in the absence or presence of CPX (20 µM) for 24 h and the whole-cell extracts were probed with antibody against OPA1. **b** *OMA1* knockout cells re-expressing *OMA1*-Flag or *OMA1*-E324Q-Flag were incubated with or without CPX (20 µM) for 24 h and the whole-cell extracts were probed with the indicated antibodies. **c, d** Cell proliferation of control and *OMA1-overexpressing* 143B and SJSA-1 cells treated with CPX (20 µM) for 24 h was measured using an EdU cell proliferation kit with Alexa Fluor 488 (**c**). Scale bar, 200 µm. EdU incorporation was quantified using ImageJ Plus software (**d**). Data were shown as mean ± SD, and statistical significance was assessed by two-tailed unpaired Student’s t-test (n=3, ***P* < 0.01, ****P* < 0.001). **e, f** Representative images (**e**) and quantification (**f**) of colony formation assay of control or *OMA1-deficient* cells. Data were shown as mean ± SD, and statistical significance was assessed by two-tailed unpaired Student’s t-test (n=3, **P* < 0.05, ***P* < 0.01, ****P* < 0.001, *****P* < 0.0001).
