# Supplementary material for: Inhibition of mitochondrial OMA1 ameliorates osteosarcoma tumorigenesis
Source: Cell Death Dis. 2024 Nov 1;15(11):786. doi: 10.1038/s41419-024-07127-1 (PMC11530700; doi:10.1038/s41419-024-07127-1)

Fig. 1C

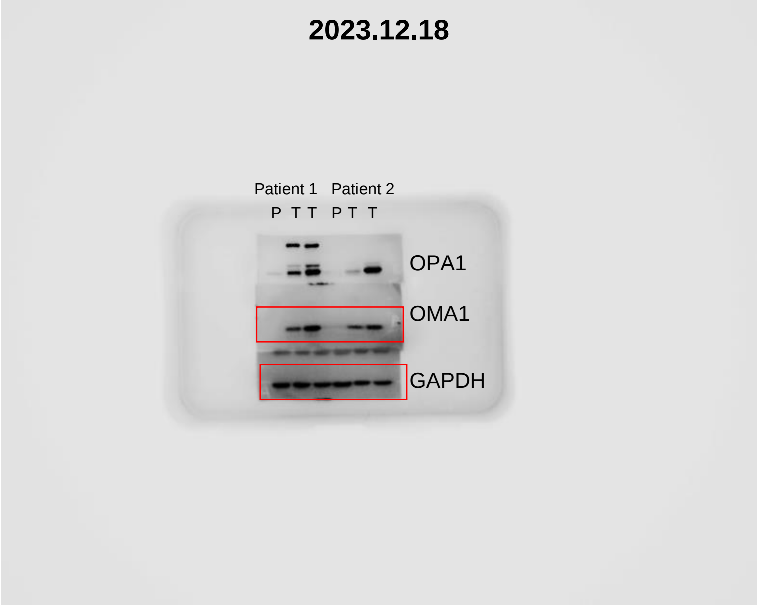
2023.12.18
Patient 1
Patient 2
P T T
P T T
OPA1
OMA1
GAPDH

Fig. 2C

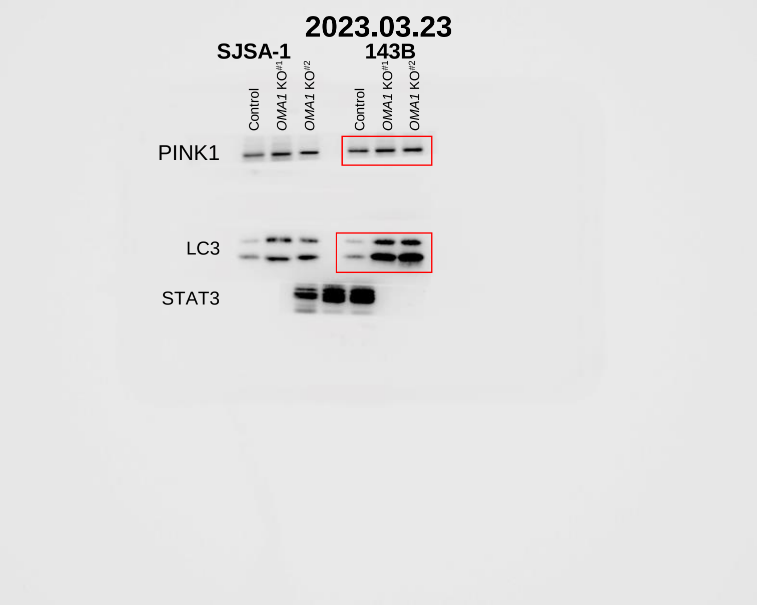
2023.03.23
SJSA-1
143B
Control
OMA1 KO#1
OMA1 KO#2
Control
OMA1 KO#1
OMA1 KO#2
PINK1
LC3
STAT3

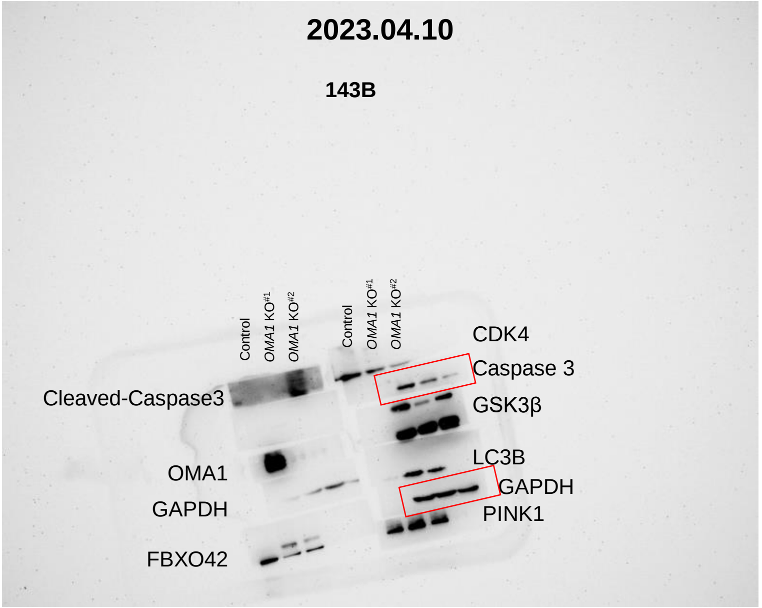
2023.04.10
143B
Control
OMA1 KO#1
OMA1 KO#2
Control
OMA1 KO#1
OMA1 KO#2
CDK4
Caspase 3
Cleaved-Caspase3
GSK3β
OMA1
LC3B
GAPDH
GAPDH
PINK1
FBXO42

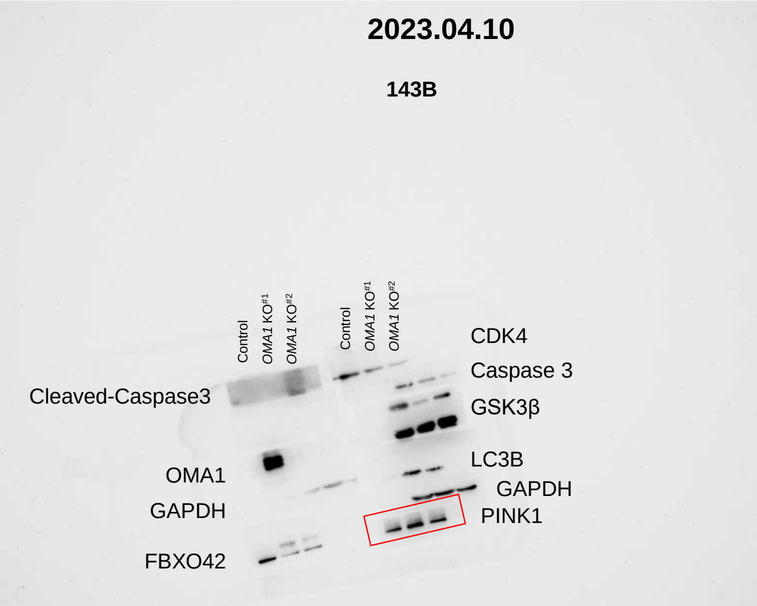
2023.04.10
143B
Control
OMA1 KO#1
OMA1 KO#2
Control
OMA1 KO#1
OMA1 KO#2
CDK4
Caspase 3
Cleaved-Caspase3
GSK3β
OMA1
LC3B
GAPDH
GAPDH
PINK1
FBXO42

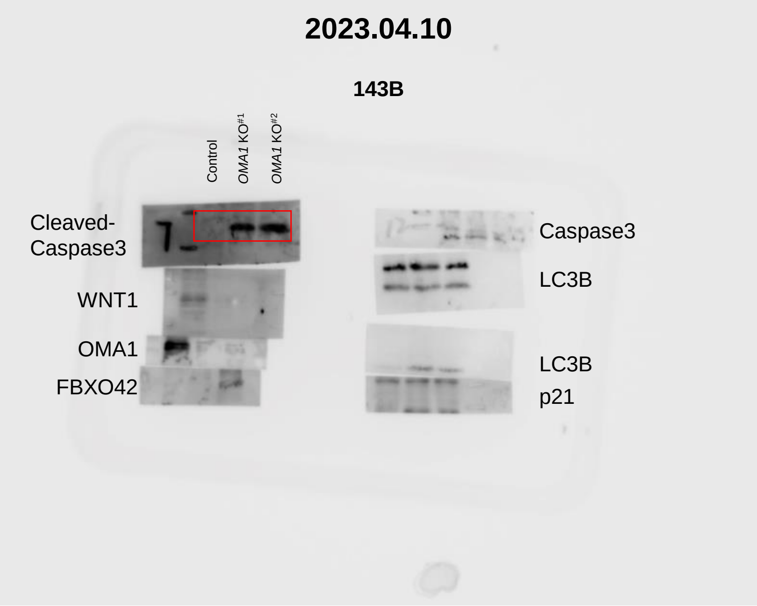
2023.04.10
143B
Control
OMA1 KO#1
OMA1 KO#2
Cleaved-
Caspase3
Caspase3
WNT1
LC3B
OMA1
FBXO42
LC3B
p21

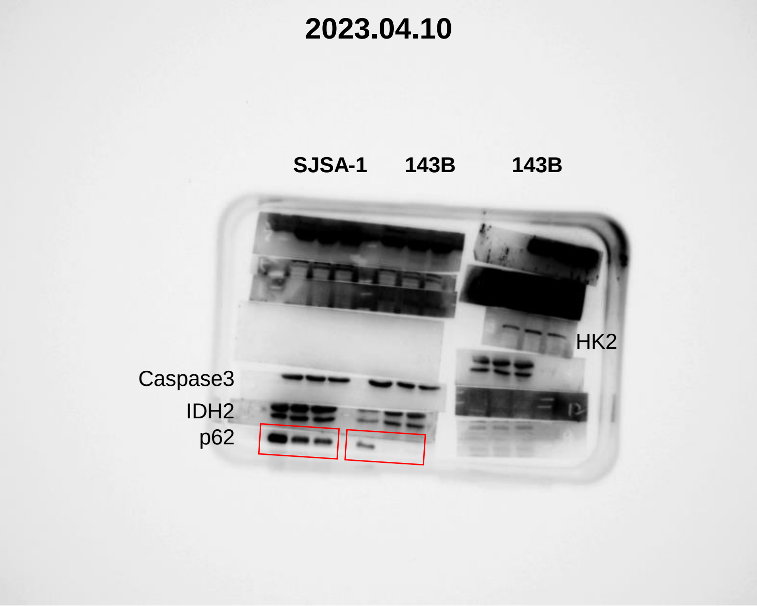
2023.04.10
SJSA-1
143B
143B
HK2
Caspase3
IDH2
p62

Fig. 2C

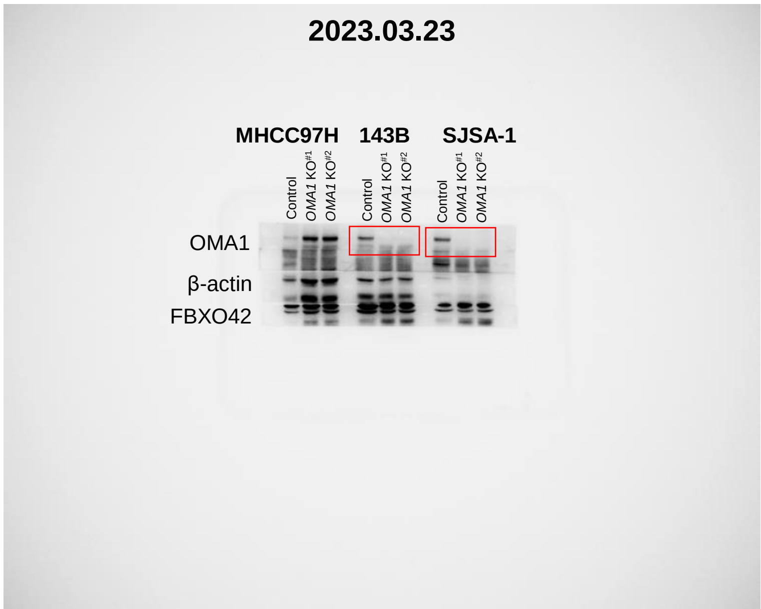
2023.03.23
MHCC97H
143B
SJSA-1
Control
OMA1 KO#1
OMA1 KO#2
OMA1
β-actin
FBXO42

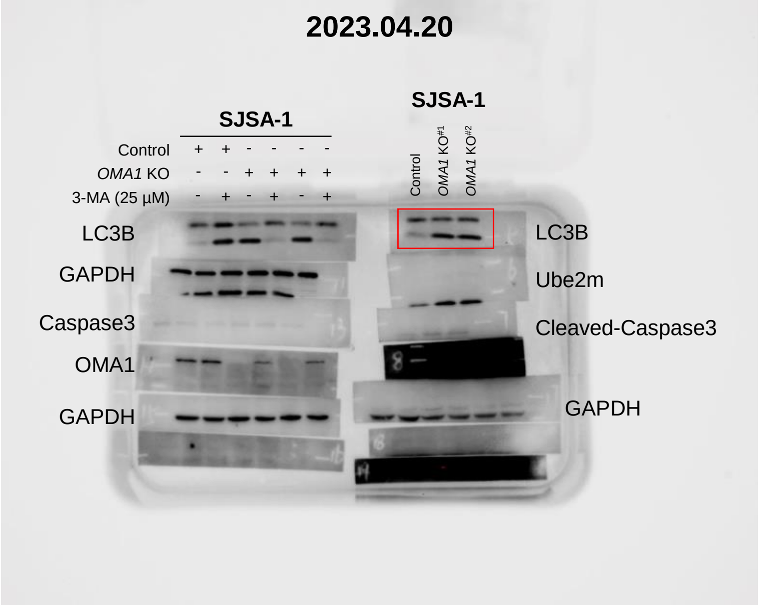
2023.04.20
SJSA-1
Control
OMA1 KO
3-MA (25 μM)
+ + - - - -
- - + + + +
- + - + - +
SJSA-1
Control
OMA1 KO#1
OMA1 KO#2
LC3B
GAPDH
Caspase3
OMA1
GAPDH
LC3B
Ube2m
Cleaved-Caspase3
GAPDH

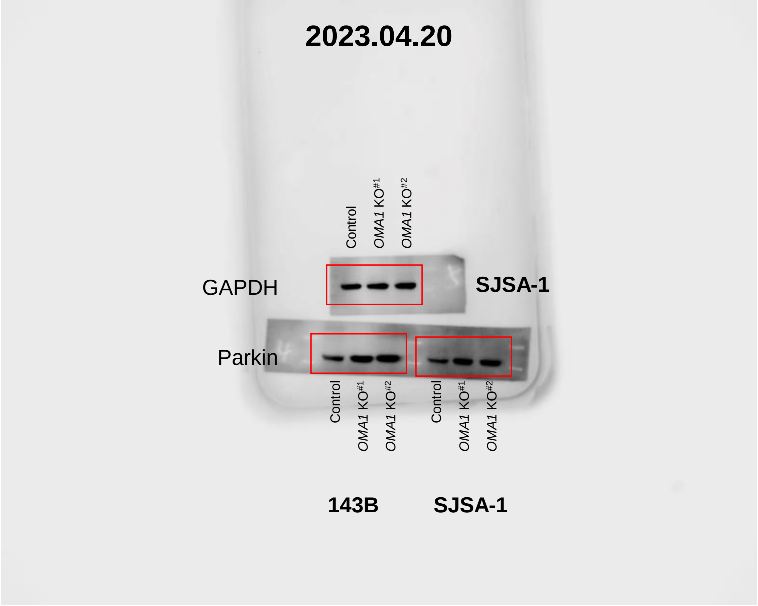
2023.04.20
Control
OMA1 KO#1
OMA1 KO#2
GAPDH
SJSA-1
Parkin
Control
OMA1 KO#1
OMA1 KO#2
Control
OMA1 KO#1
OMA1 KO#2
143B
SJSA-1

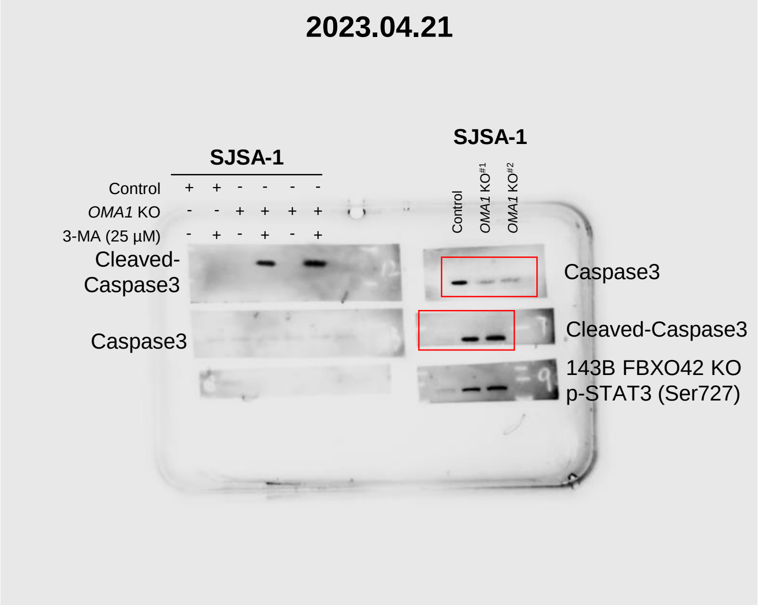
2023.04.21
SJSA-1
Control
OMA1 KO
3-MA (25 μM)
+ + - - - -
- - + + + +
- + - + - +
Cleaved-
Caspase3
Caspase3
SJSA-1
Control
OMA1 KO#1
OMA1 KO#2
Caspase3
Cleaved-Caspase3
143B FBXO42 KO
p-STAT3 (Ser727)

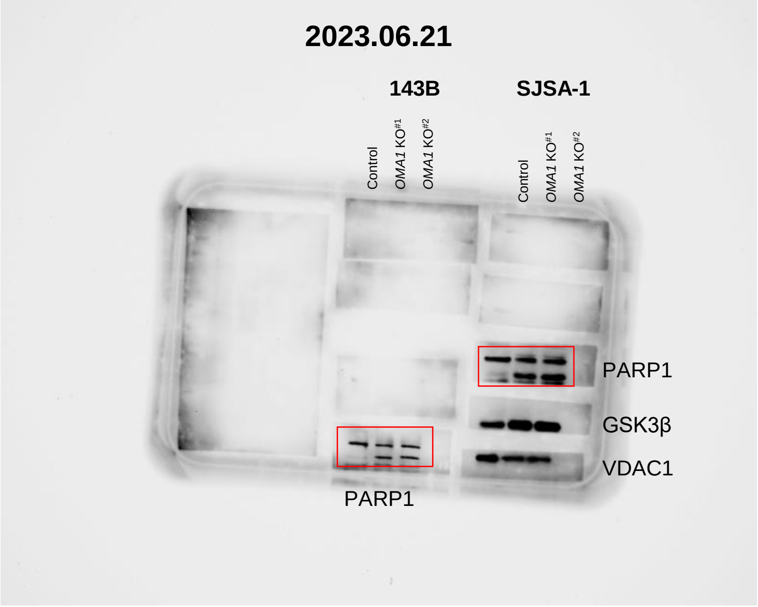
2023.06.21
143B
SJSA-1
Control
OMA1 KO#1
OMA1 KO#2
Control
OMA1 KO#1
OMA1 KO#2
PARP1
GSK3β
VDAC1
PARP1

Fig. 2F

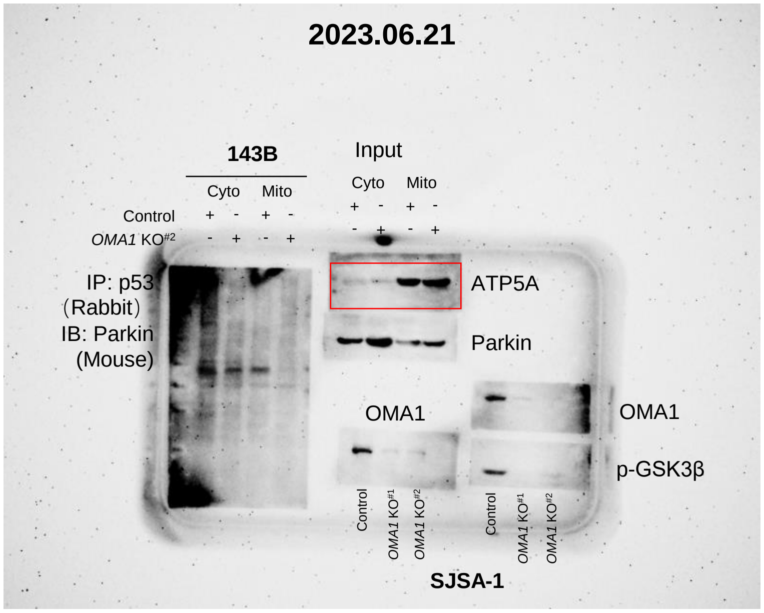
2023.06.21
143B
Cyto
Mito
Control
OMA1 KO#2
+ - + -
- + - +
Input
Cyto
Mito
+ - + -
- + - +
IP: p53
(Rabbit)
IB: Parkin
(Mouse)
ATP5A
Parkin
OMA1
OMA1
p-GSK3β
Control
OMA1 KO#1
OMA1 KO#2
Control
OMA1 KO#1
OMA1 KO#2
SJSA-1

Fig. 2F

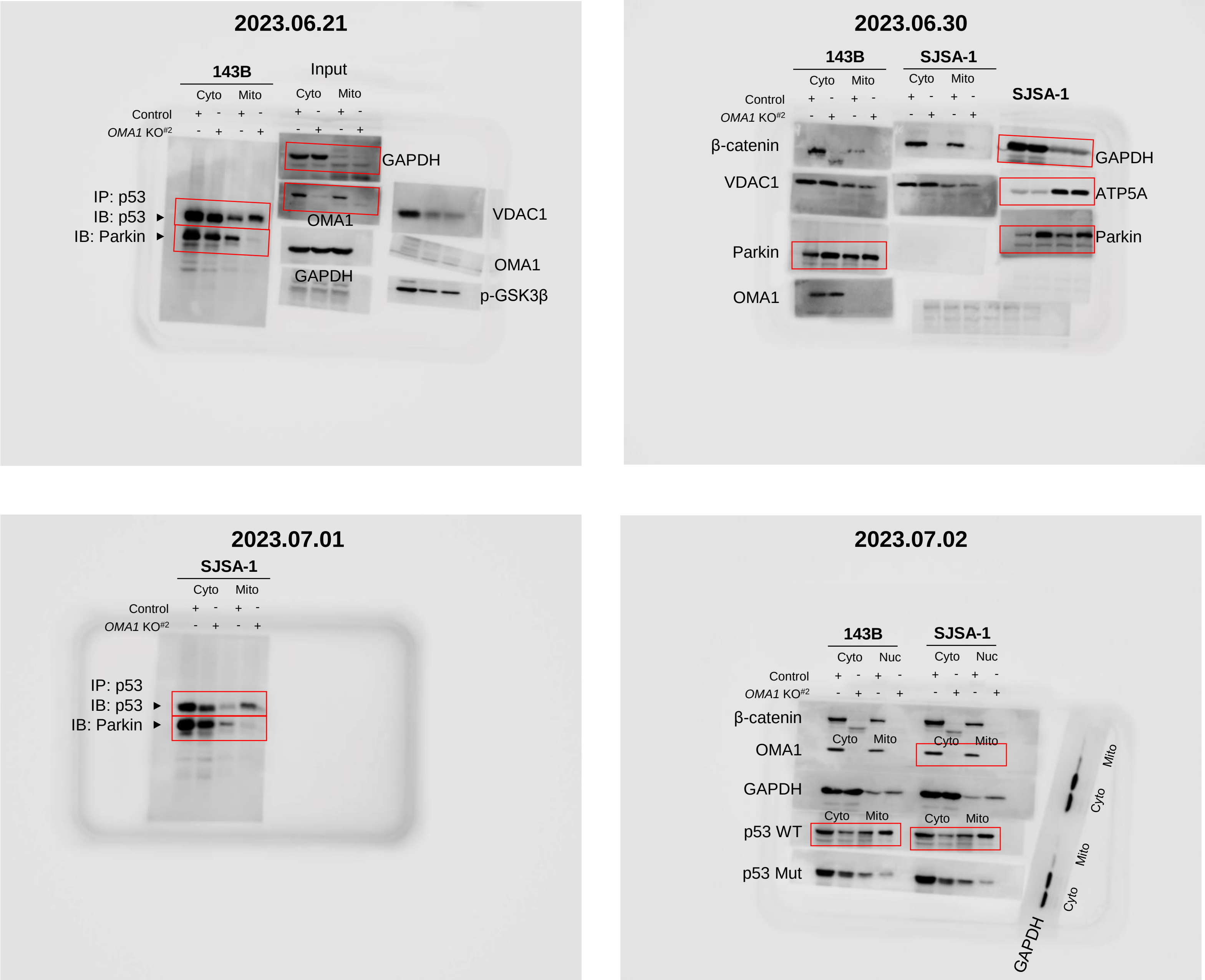
2023.06.21
143B
Input
Cyto
Mito
Control
OMA1 KO#2
IP: p53
IB: p53
IB: Parkin
GAPDH
OMA1
VDAC1
OMA1
GAPDH
p-GSK3β
2023.06.30
143B
SJSA-1
SJSA-1
Cyto
Mito
Control
OMA1 KO#2
β-catenin
VDAC1
Parkin
OMA1
GAPDH
ATP5A
Parkin
2023.07.01
SJSA-1
Cyto
Mito
Control
OMA1 KO#2
IP: p53
IB: p53
IB: Parkin
2023.07.02
143B
SJSA-1
Cyto
Nuc
Control
OMA1 KO#2
β-catenin
OMA1
GAPDH
p53 WT
p53 Mut
Mito
Cyto
GAPDH

Fig. 3G

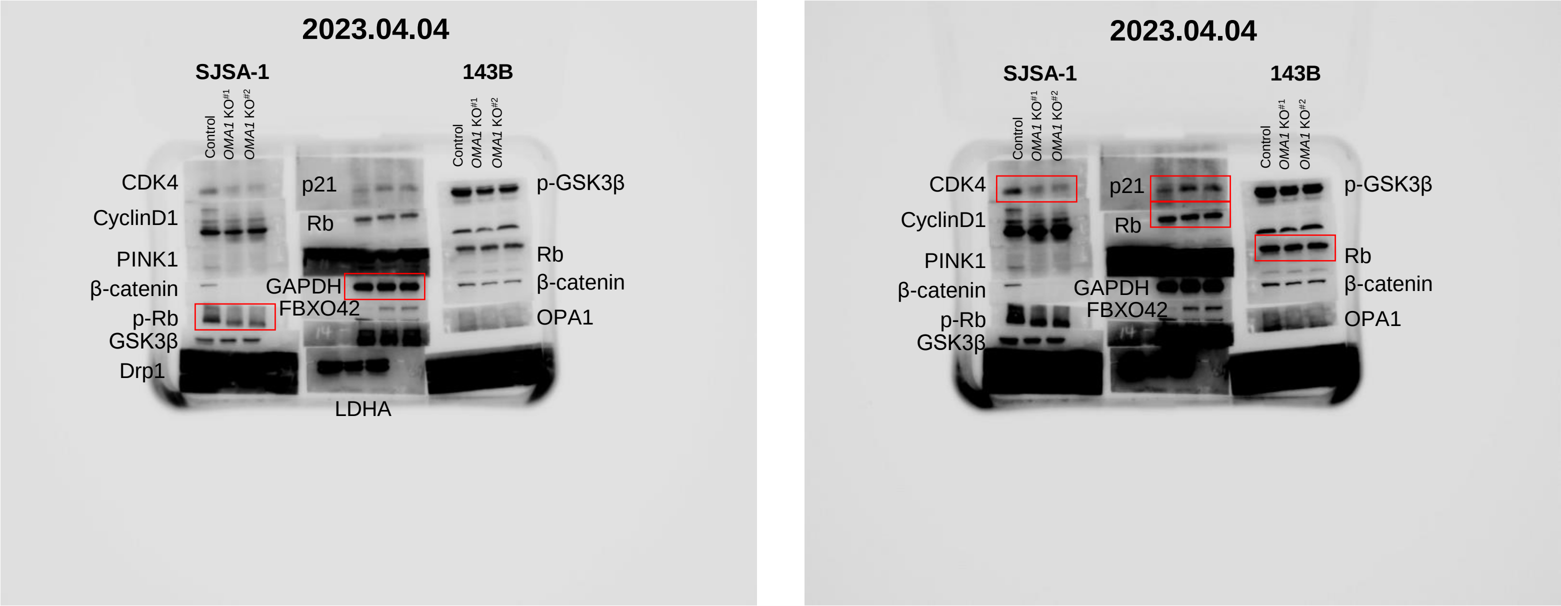
2023.04.04
SJSA-1
143B
Control
OMA1 KO#1
OMA1 KO#2
CDK4
CyclinD1
PINK1
β-catenin
p-Rb
GSK3β
Drp1
p21
Rb
GAPDH
FBXO42
LDHA
p-GSK3β
Rb
β-catenin
OPA1
2023.04.04
SJSA-1
143B
Control
OMA1 KO#1
OMA1 KO#2
CDK4
CyclinD1
PINK1
β-catenin
p-Rb
GSK3β
p21
Rb
GAPDH
FBXO42
p-GSK3β
Rb
β-catenin
OPA1

Fig. 3G

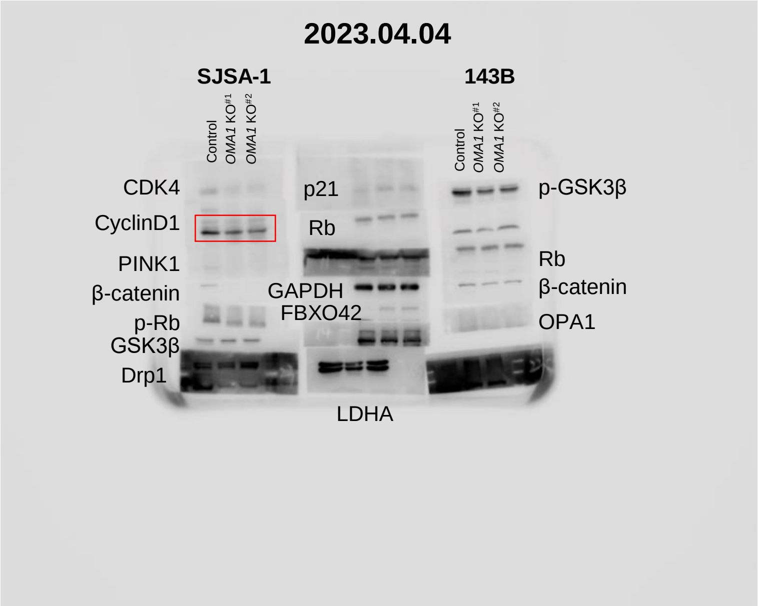
2023.04.04
SJSA-1
143B
Control
OMA1 KO[#1]
OMA1 KO[#2]
Control
OMA1 KO[#1]
OMA1 KO[#2]
CDK4
CyclinD1
PINK1
β-catenin
p-Rb
GSK3β
Drp1
p21
Rb
GAPDH
FBXO42
LDHA
p-GSK3β
Rb
β-catenin
OPA1

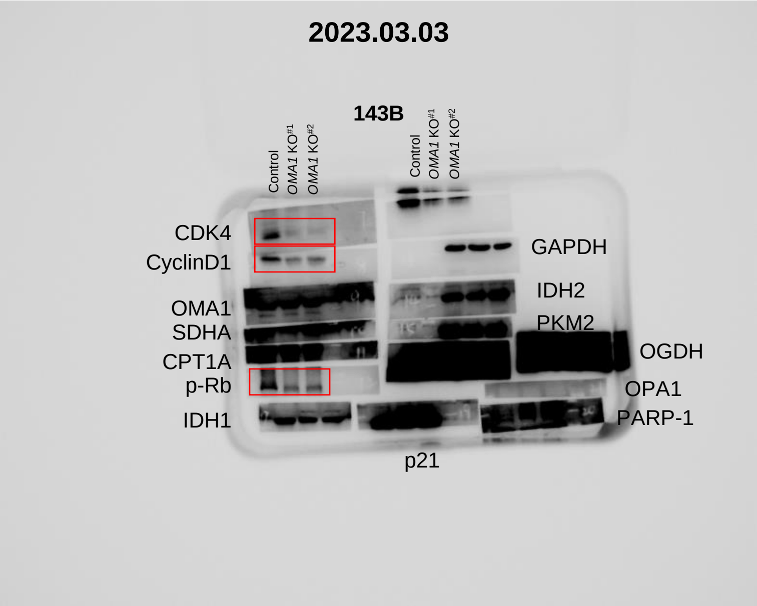
2023.03.03
143B
Control
OMA1 KO[#1]
OMA1 KO[#2]
Control
OMA1 KO[#1]
OMA1 KO[#2]
CDK4
CyclinD1
OMA1
SDHA
CPT1A
p-Rb
IDH1
p21
GAPDH
IDH2
PKM2
OGDH
OPA1
PARP-1

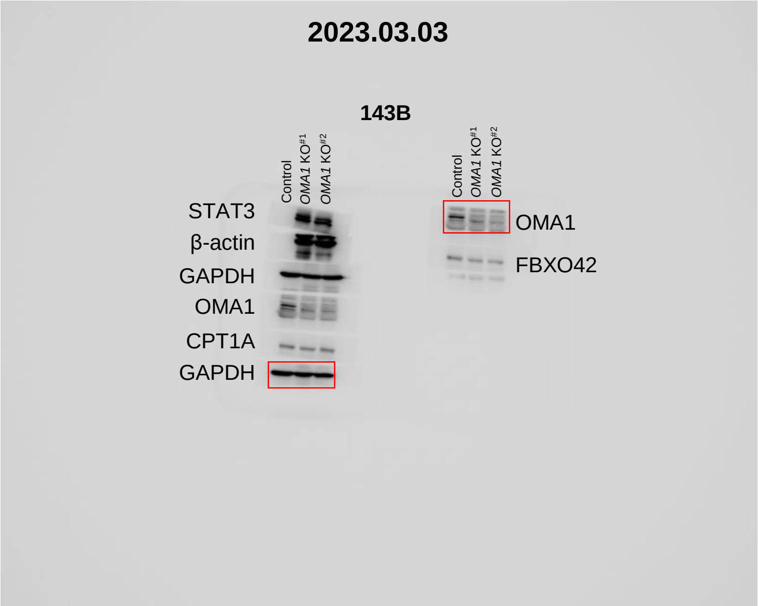
2023.03.03
143B
Control
OMA1 KO[#1]
OMA1 KO[#2]
Control
OMA1 KO[#1]
OMA1 KO[#2]
STAT3
β-actin
GAPDH
OMA1
CPT1A
GAPDH
OMA1
FBXO42

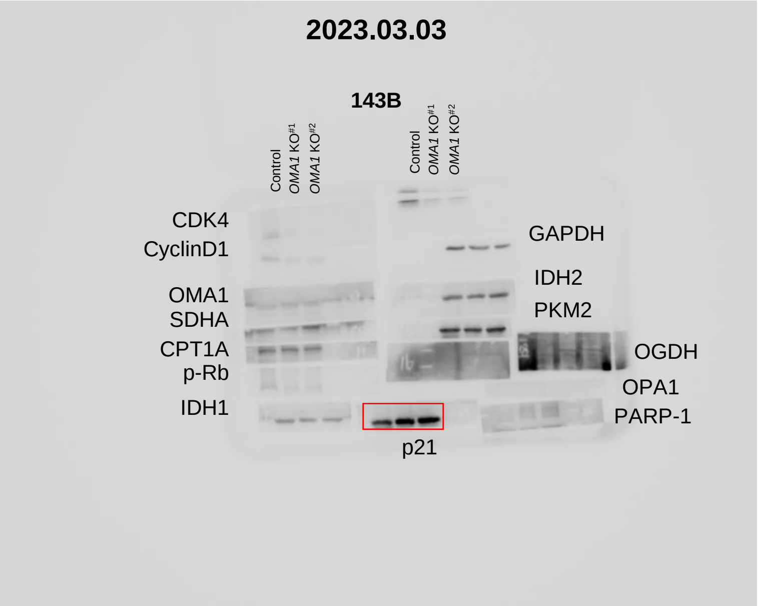
2023.03.03
143B
Control
OMA1 KO[#1]
OMA1 KO[#2]
Control
OMA1 KO[#1]
OMA1 KO[#2]
CDK4
CyclinD1
OMA1
SDHA
CPT1A
p-Rb
IDH1
p21
GAPDH
IDH2
PKM2
OGDH
OPA1
PARP-1

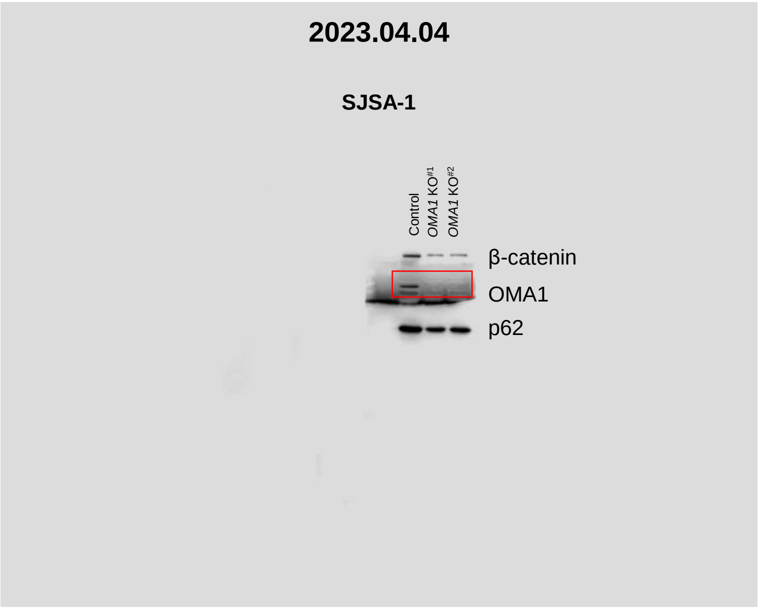
2023.04.04
SJSA-1
Control
OMA1 KO[#1]
OMA1 KO[#2]
β-catenin
OMA1
p62

Fig. 4D

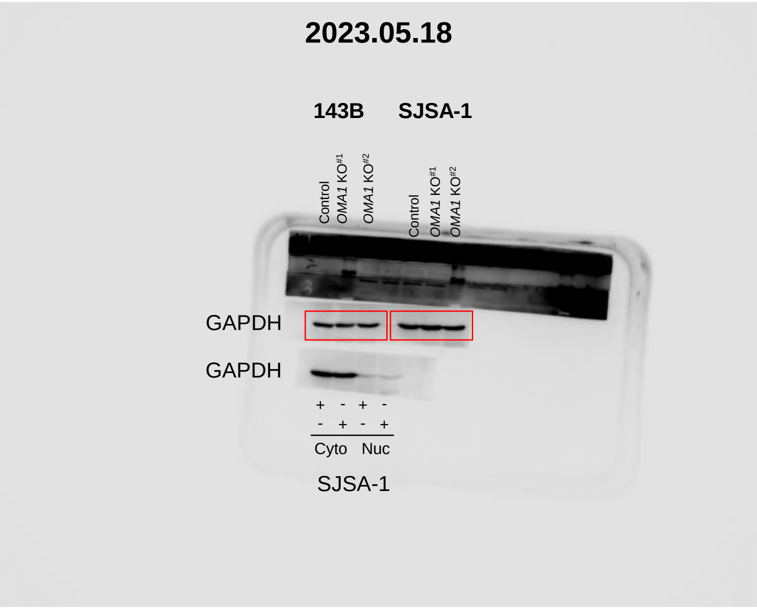
2023.05.18
143B
SJSA-1
Control
OMA1 KO[#1]
OMA1 KO[#2]
Control
OMA1 KO[#1]
OMA1 KO[#2]
GAPDH
GAPDH
+ - + -
- + - +
Cyto Nuc
SJSA-1

Fig. 4D

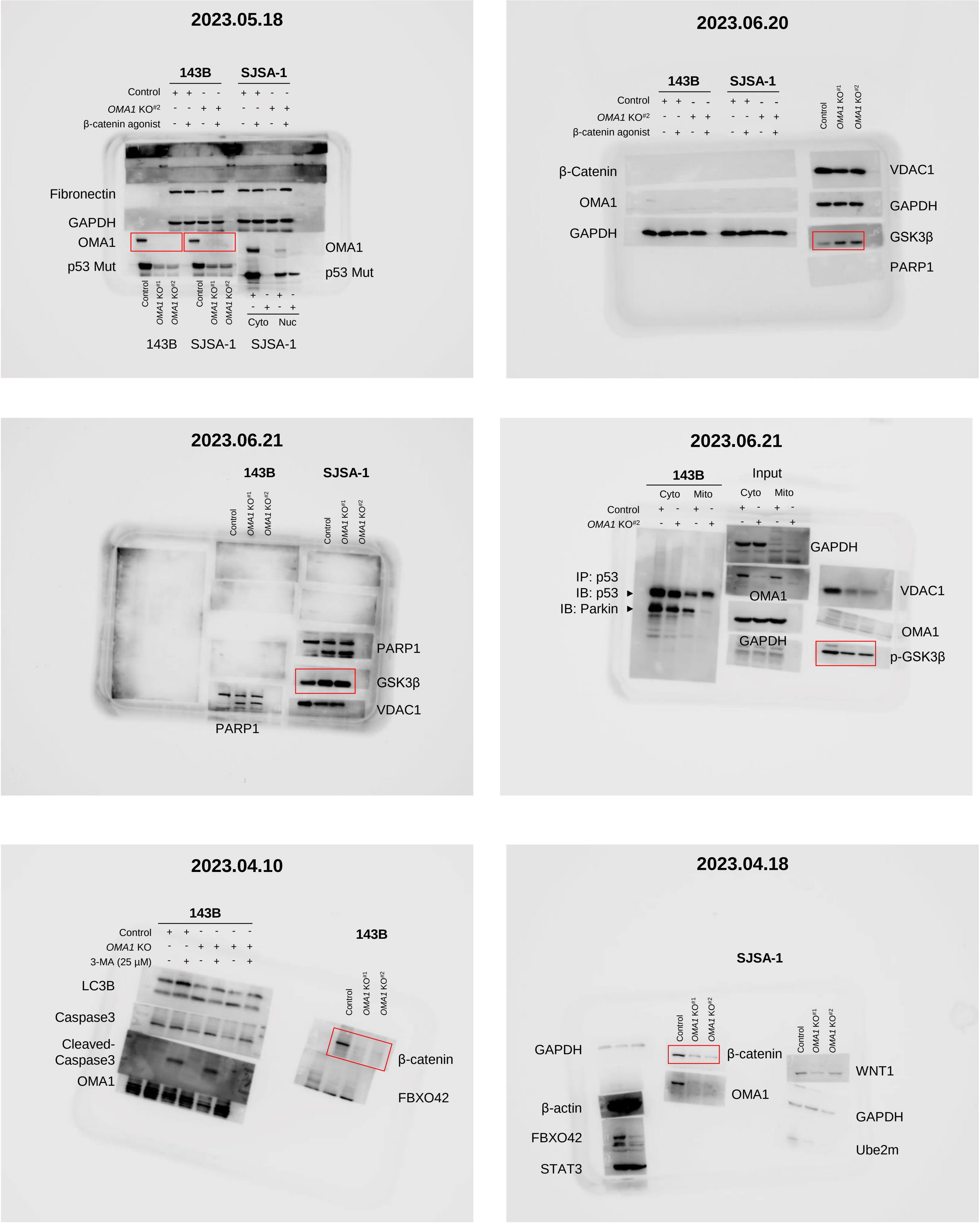
2023.05.18
143B
SJSA-1
Control
OMA1 KO#2
β-catenin agonist
Fibronectin
GAPDH
OMA1
p53 Mut
Control
OMA1 KO#1
OMA1 KO#2
Cyto
Nuc
143B
SJSA-1
SJSA-1
2023.06.20
β-Catenin
OMA1
GAPDH
VDAC1
GSK3β
PARP1
2023.06.21
PARP1
GSK3β
VDAC1
2023.06.21
Input
Cyto
Mito
IP: p53
IB: p53
IB: Parkin
p-GSK3β
2023.04.10
OMA1 KO
3-MA (25 μM)
LC3B
Caspase3
Cleaved-
Caspase3
β-catenin
FBXO42
2023.04.18
β-actin
STAT3
WNT1
Ube2m

Fig. 4D

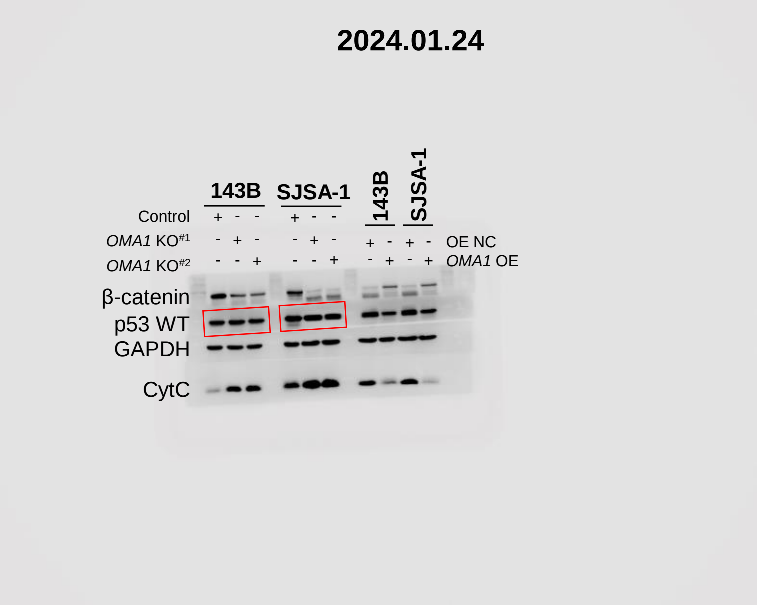
2024.01.24
143B
SJSA-1
143B
SJSA-1
Control
OMA1 KO#1
OMA1 KO#2
OE NC
OMA1 OE
β-catenin
p53 WT
GAPDH
CytC

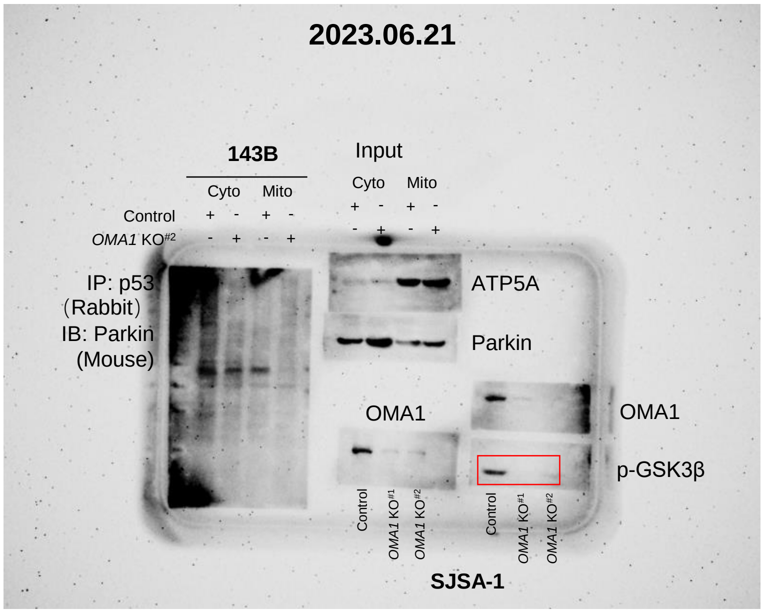
2023.06.21
143B
Input
Cyto
Mito
Control
OMA1 KO#2
IP: p53
(Rabbit)
IB: Parkin
(Mouse)
ATP5A
Parkin
OMA1
OMA1
p-GSK3β
Control
OMA1 KO#1
OMA1 KO#2
SJSA-1

Fig. 4E

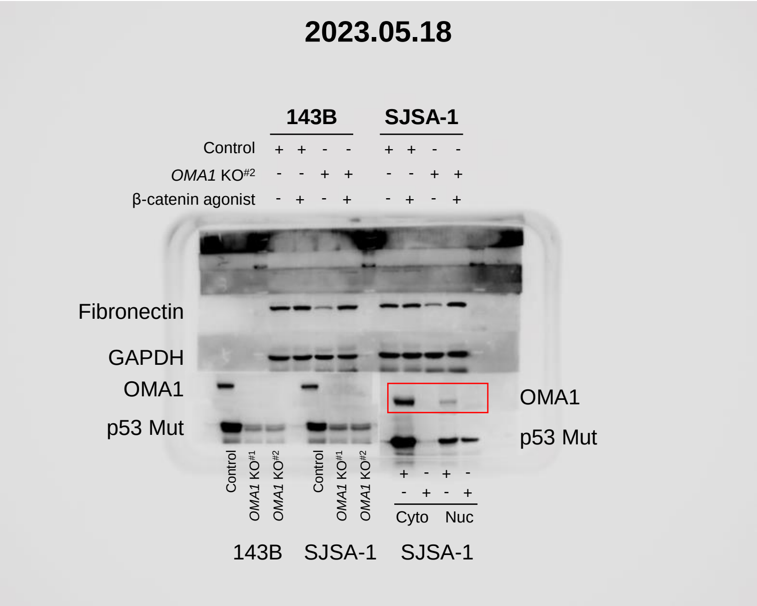
2023.05.18
143B
SJSA-1
Control
OMA1 KO#2
β-catenin agonist
Fibronectin
GAPDH
OMA1
p53 Mut
OMA1
p53 Mut
Control
OMA1 KO#1
OMA1 KO#2
Cyto
Nuc
143B
SJSA-1
SJSA-1

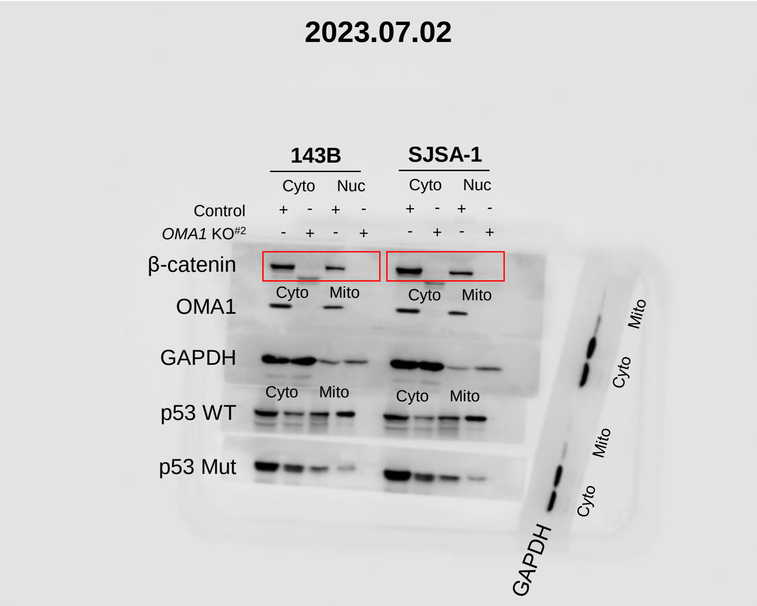
2023.07.02
143B
SJSA-1
Cyto
Nuc
Control
OMA1 KO#2
β-catenin
OMA1
GAPDH
p53 WT
p53 Mut
Mito
GAPDH

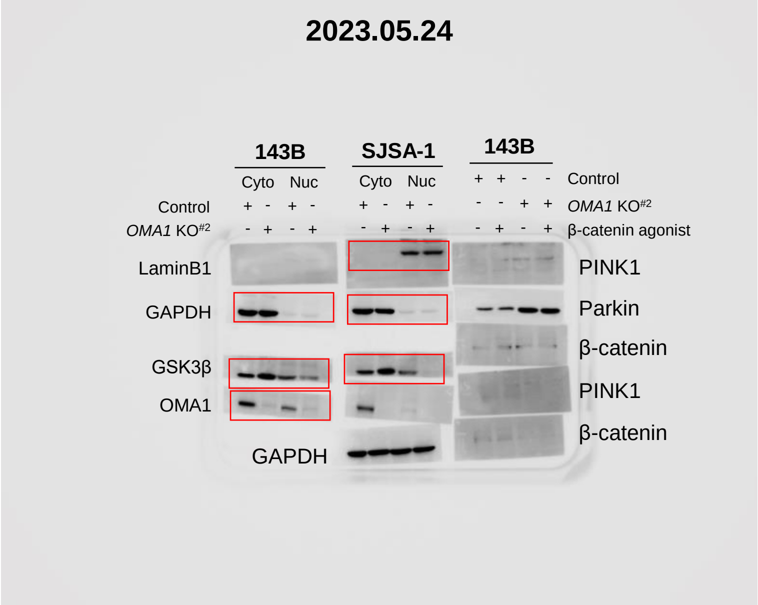
2023.05.24
143B
SJSA-1
143B
Cyto
Nuc
Control
OMA1 KO#2
β-catenin agonist
LaminB1
GAPDH
GSK3β
OMA1
GAPDH
PINK1
Parkin
β-catenin
PINK1
β-catenin

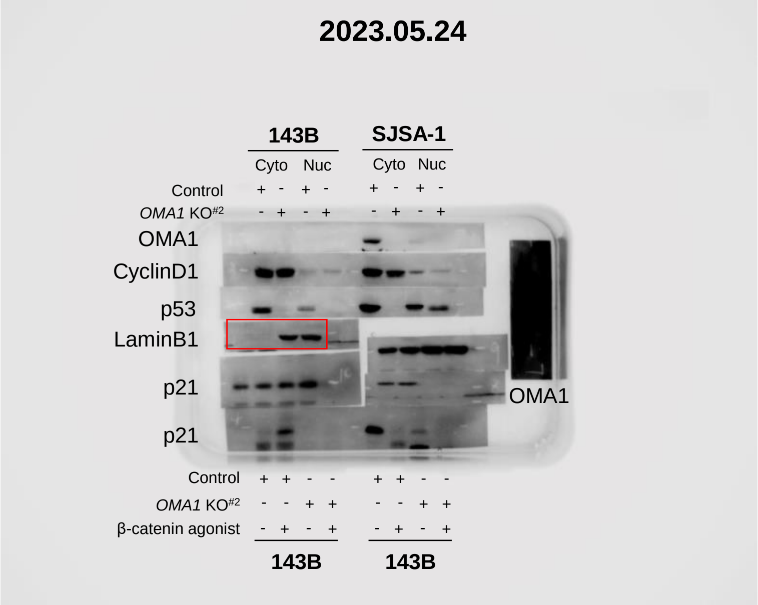
2023.05.24
143B
SJSA-1
Cyto
Nuc
Control
OMA1 KO#2
OMA1
CyclinD1
p53
LaminB1
p21
p21
OMA1
β-catenin agonist
143B
143B

Fig. 4J

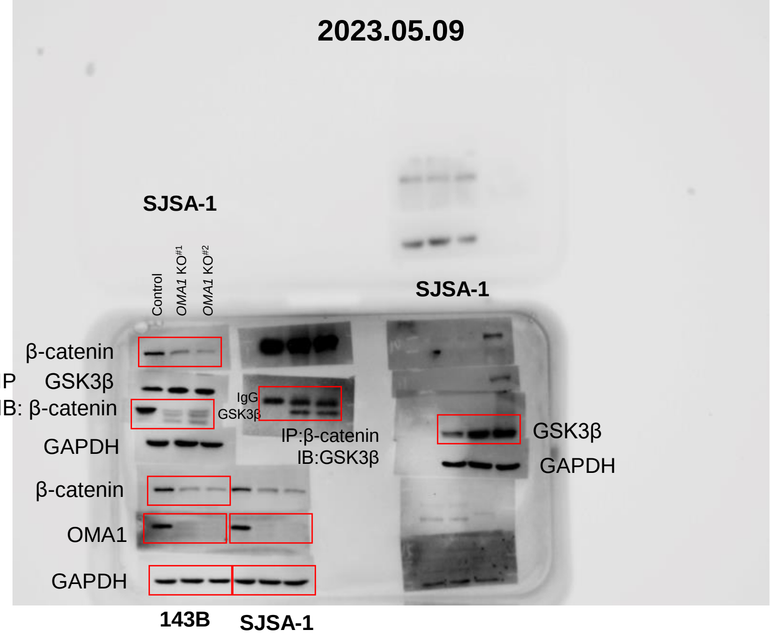
2023.05.09
SJSA-1
Control
OMA1 KO#1
OMA1 KO#2
SJSA-1
β-catenin
IP GSK3β
IB: β-catenin
GAPDH
IgG
GSK3β
IP:β-catenin
IB:GSK3β
GSK3β
GAPDH
β-catenin
OMA1
GAPDH
143B
SJSA-1

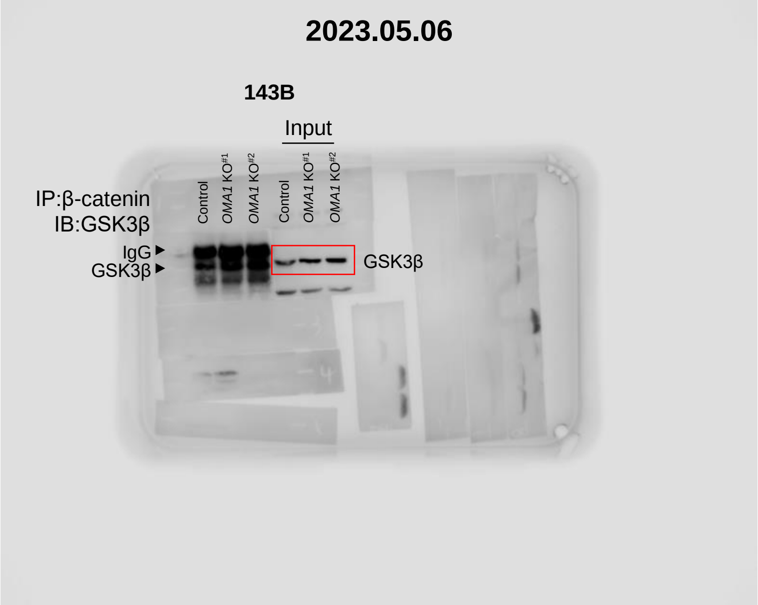
2023.05.06
143B
Input
IP:β-catenin
IB:GSK3β
Control
OMA1 KO#1
OMA1 KO#2
Control
OMA1 KO#1
OMA1 KO#2
IgG
GSK3β
GSK3β

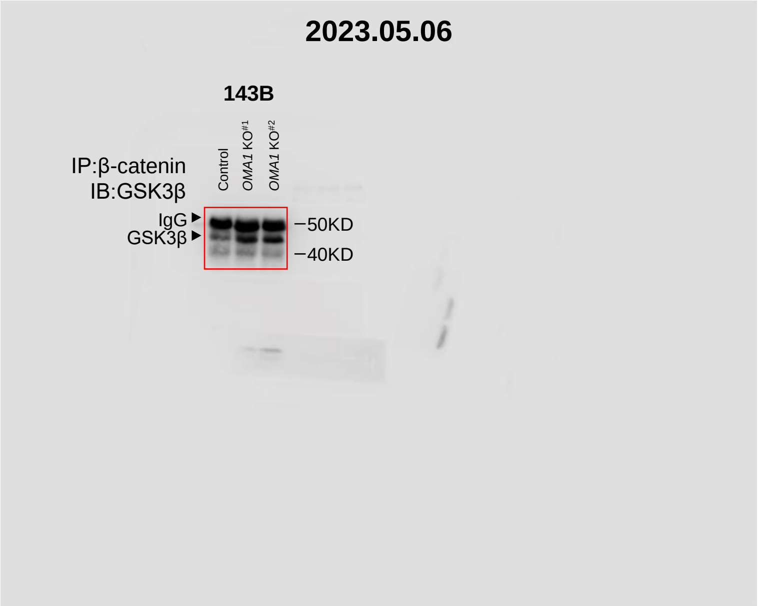
2023.05.06
143B
IP:β-catenin
IB:GSK3β
Control
OMA1 KO#1
OMA1 KO#2
IgG
GSK3β
−50KD
−40KD

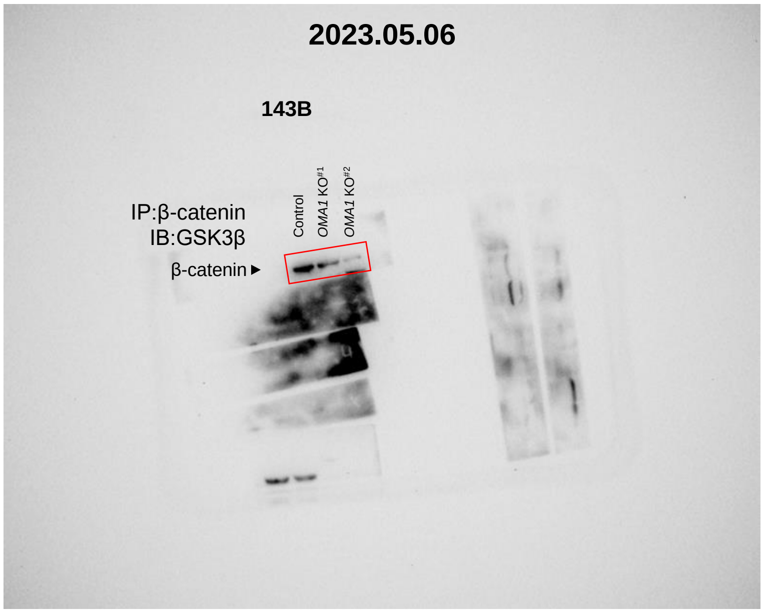
2023.05.06
143B
IP:β-catenin
IB:GSK3β
Control
OMA1 KO#1
OMA1 KO#2
β-catenin

Fig. 4K

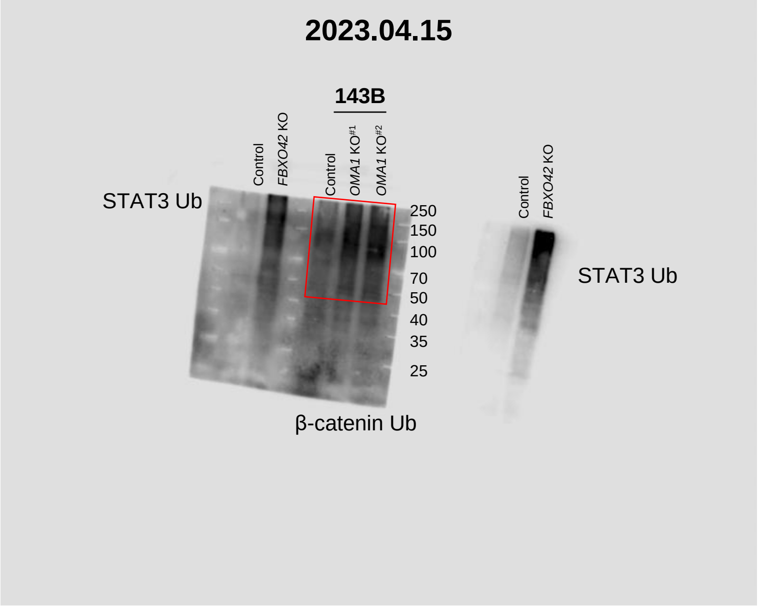
2023.04.15
143B
Control
FBXO42 KO
Control
OMA1 KO#1
OMA1 KO#2
STAT3 Ub
250
150
100
70
50
40
35
25
β-catenin Ub
Control
FBXO42 KO
STAT3 Ub

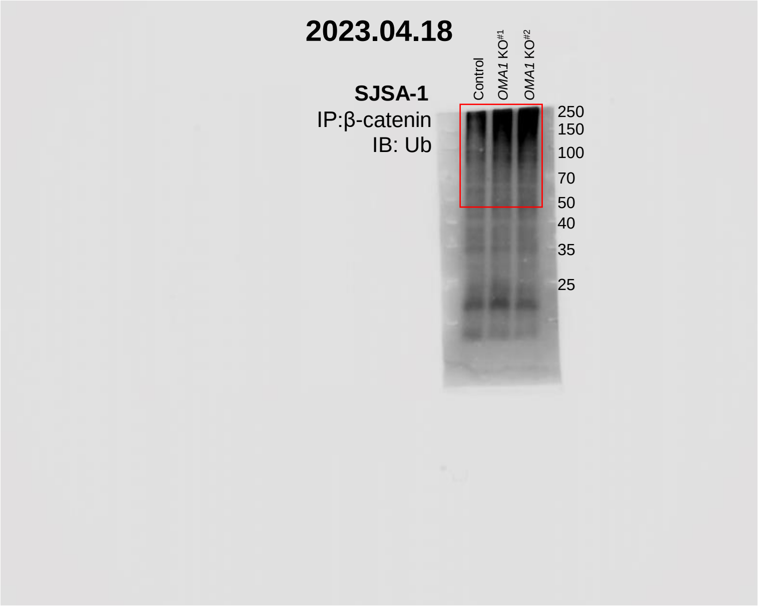
2023.04.18
SJSA-1
IP:β-catenin
IB: Ub
Control
OMA1 KO#1
OMA1 KO#2
250
150
100
70
50
40
35
25

Fig. 4K

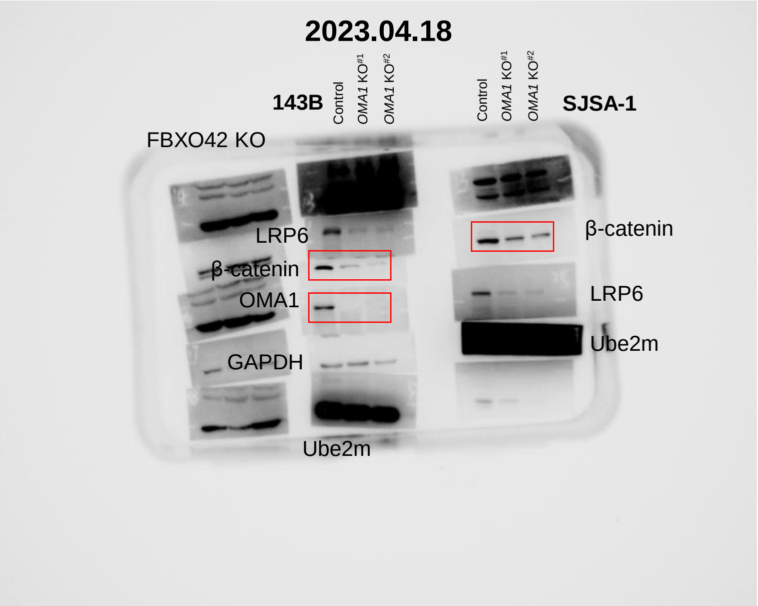
2023.04.18
143B
Control
OMA1 KO#1
OMA1 KO#2
Control
OMA1 KO#1
OMA1 KO#2
SJSA-1
FBXO42 KO
LRP6
β-catenin
OMA1
GAPDH
Ube2m
β-catenin
LRP6
Ube2m

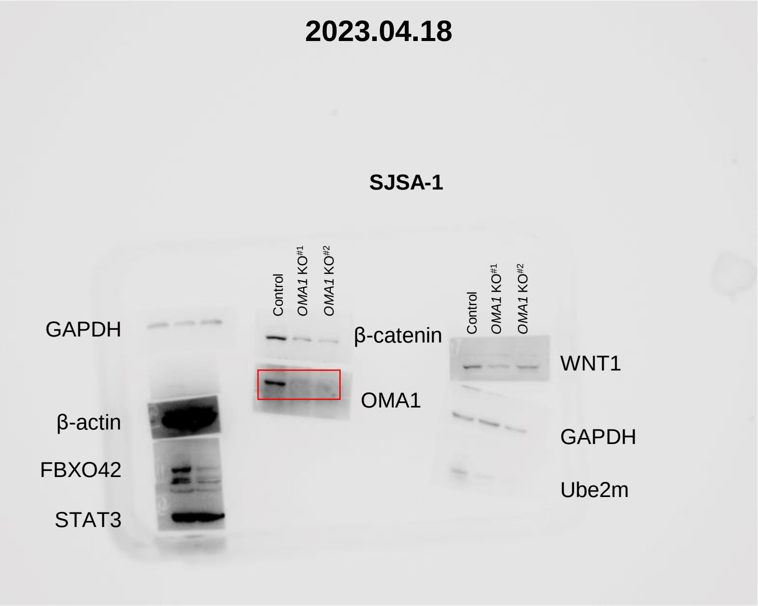
2023.04.18
SJSA-1
Control
OMA1 KO#1
OMA1 KO#2
Control
OMA1 KO#1
OMA1 KO#2
GAPDH
β-catenin
WNT1
β-actin
OMA1
GAPDH
FBXO42
Ube2m
STAT3

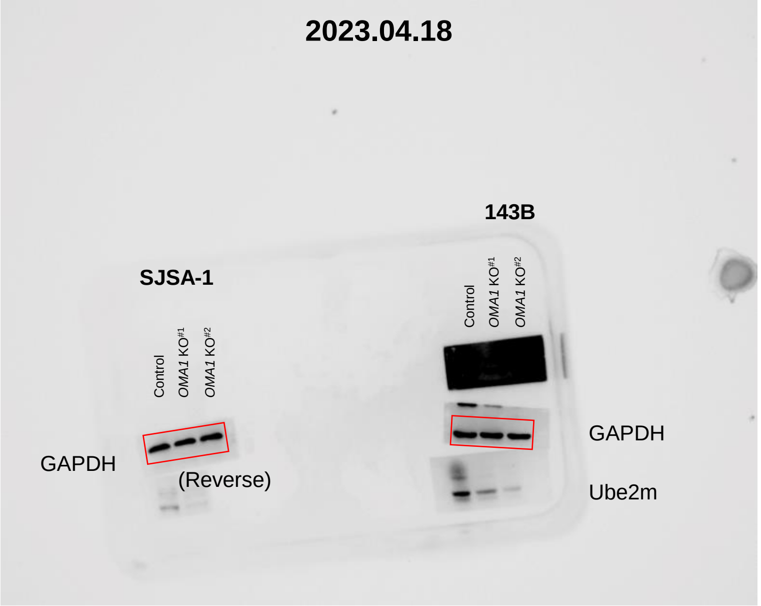
2023.04.18
143B
SJSA-1
Control
OMA1 KO#1
OMA1 KO#2
Control
OMA1 KO#1
OMA1 KO#2
GAPDH
(Reverse)
GAPDH
Ube2m

Fig. 5E

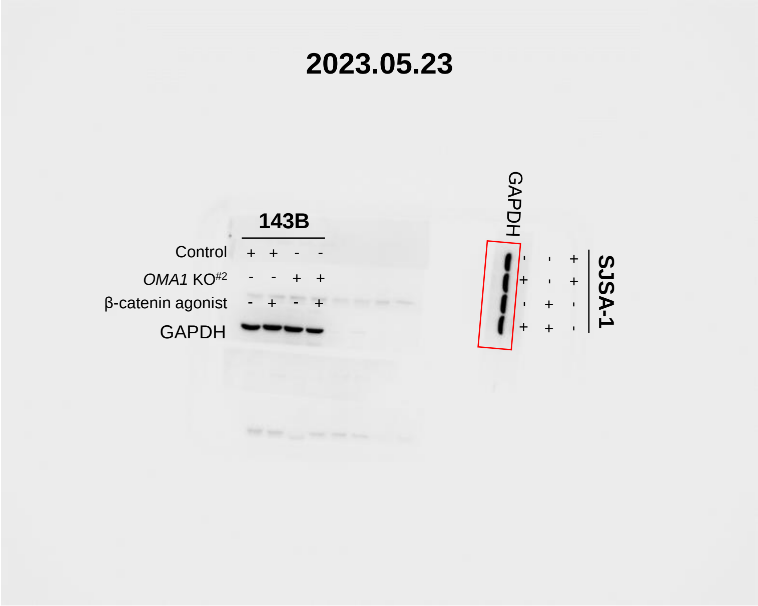
2023.05.23
143B
Control + + - -
OMA1 KO#2 - - + +
β-catenin agonist - + - +
GAPDH
GAPDH
SJSA-1

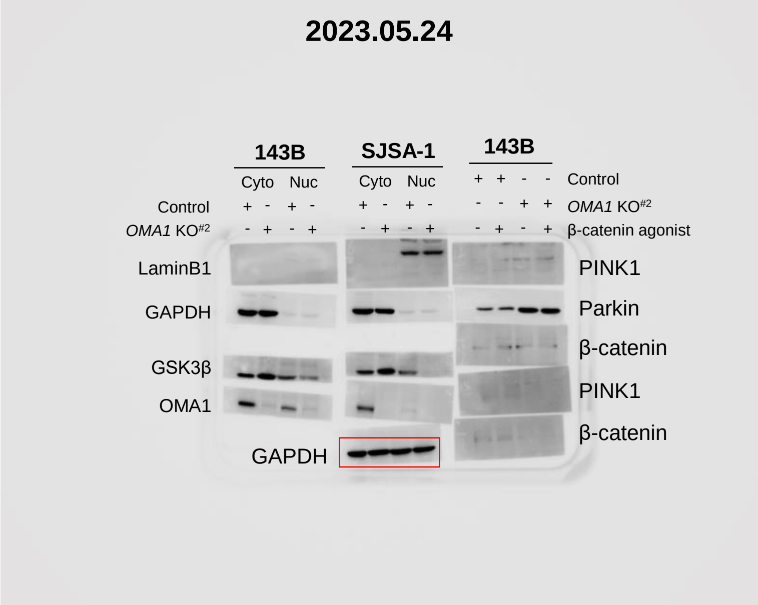
2023.05.24
143B
SJSA-1
143B
Cyto Nuc
Cyto Nuc
Control + - + -
OMA1 KO#2 - + - +
+ - + -
- + - +
+ + - - Control
- - + + OMA1 KO#2
- + - + β-catenin agonist
LaminB1
GAPDH
GSK3β
OMA1
GAPDH
PINK1
Parkin
β-catenin
PINK1
β-catenin

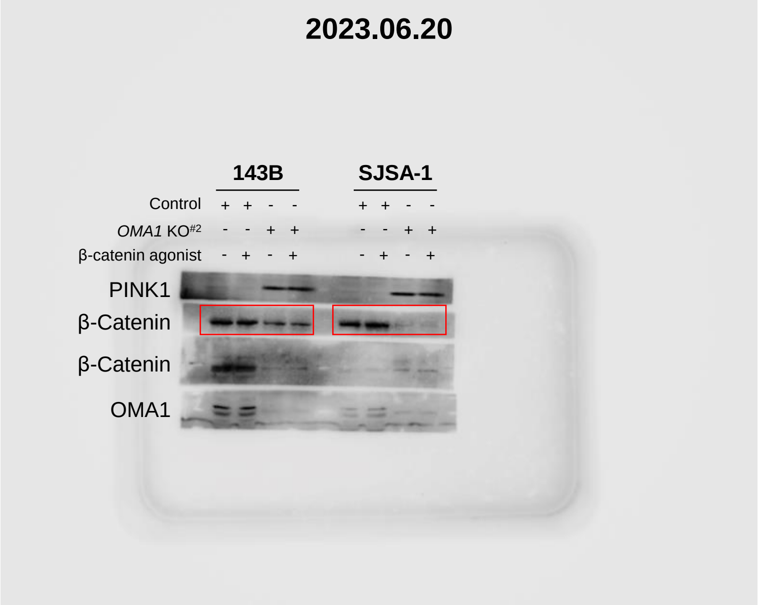
2023.06.20
143B
SJSA-1
Control + + - -
OMA1 KO#2 - - + +
β-catenin agonist - + - +
+ + - -
- - + +
- + - +
PINK1
β-Catenin
β-Catenin
OMA1

Fig. 5E

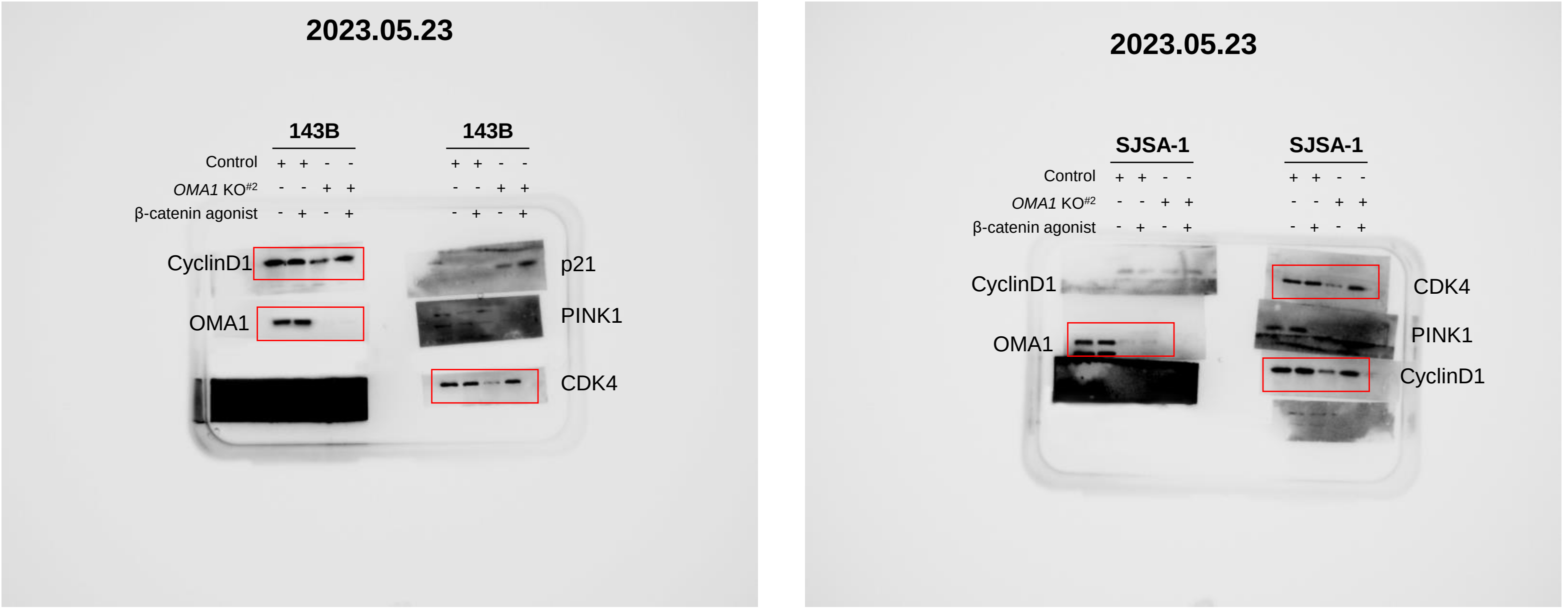
2023.05.23
143B
143B
Control + + - -
OMA1 KO#2 - - + +
β-catenin agonist - + - +
CyclinD1
OMA1
p21
PINK1
CDK4
2023.05.23
SJSA-1
SJSA-1
Control + + - -
OMA1 KO#2 - - + +
β-catenin agonist - + - +
CyclinD1
OMA1
CDK4
PINK1
CyclinD1

Fig. 5F

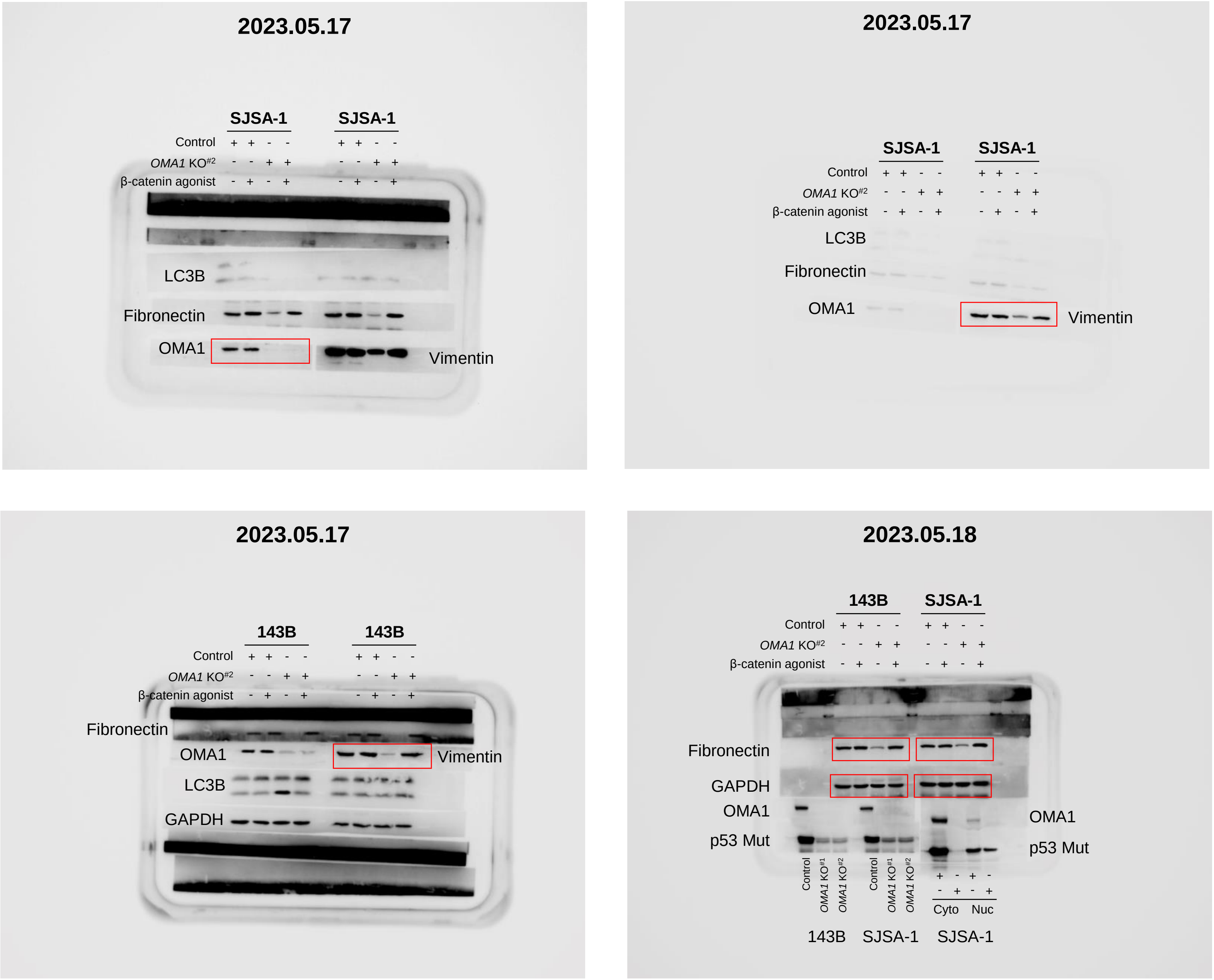
2023.05.17
SJSA-1
SJSA-1
Control + + - -
OMA1 KO#2 - - + +
β-catenin agonist - + - +
LC3B
Fibronectin
OMA1
Vimentin
2023.05.17
SJSA-1
SJSA-1
Control + + - -
OMA1 KO#2 - - + +
β-catenin agonist - + - +
LC3B
Fibronectin
OMA1
Vimentin
2023.05.17
143B
143B
Control + + - -
OMA1 KO#2 - - + +
β-catenin agonist - + - +
Fibronectin
OMA1
Vimentin
LC3B
GAPDH
2023.05.18
143B
SJSA-1
Control + + - -
OMA1 KO#2 - - + +
β-catenin agonist - + - +
Fibronectin
GAPDH
OMA1
p53 Mut
OMA1
p53 Mut
Control
OMA1 KO#1
OMA1 KO#2
Control
OMA1 KO#1
OMA1 KO#2
Cyto
Nuc
143B
SJSA-1
SJSA-1

Fig. 5F

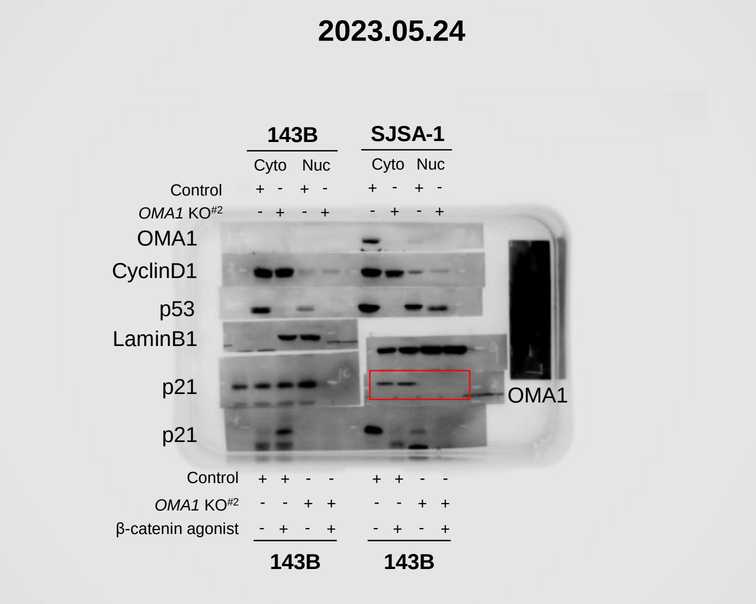
2023.05.24
143B
SJSA-1
Cyto Nuc
Cyto Nuc
Control
OMA1 KO#2
OMA1
CyclinD1
p53
LaminB1
p21
p21
OMA1
Control
OMA1 KO#2
β-catenin agonist
143B
143B

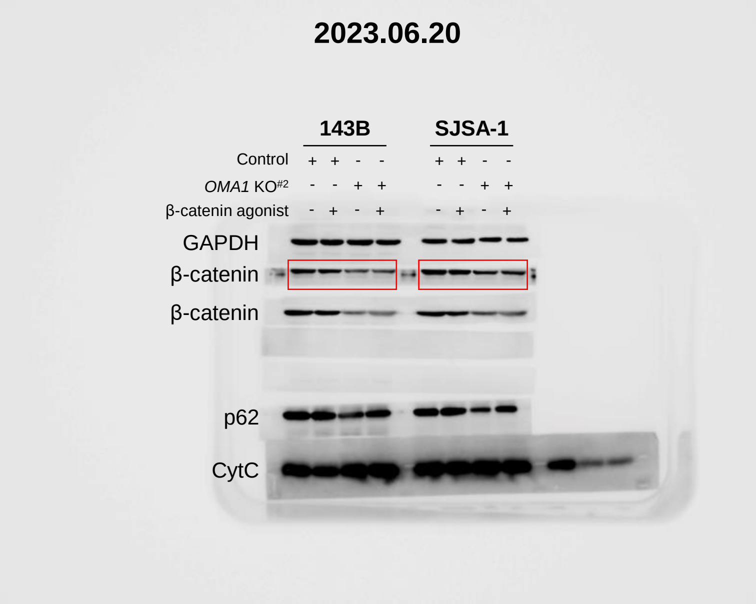
2023.06.20
143B
SJSA-1
Control
OMA1 KO#2
β-catenin agonist
GAPDH
β-catenin
β-catenin
p62
CytC

Fig. 5I

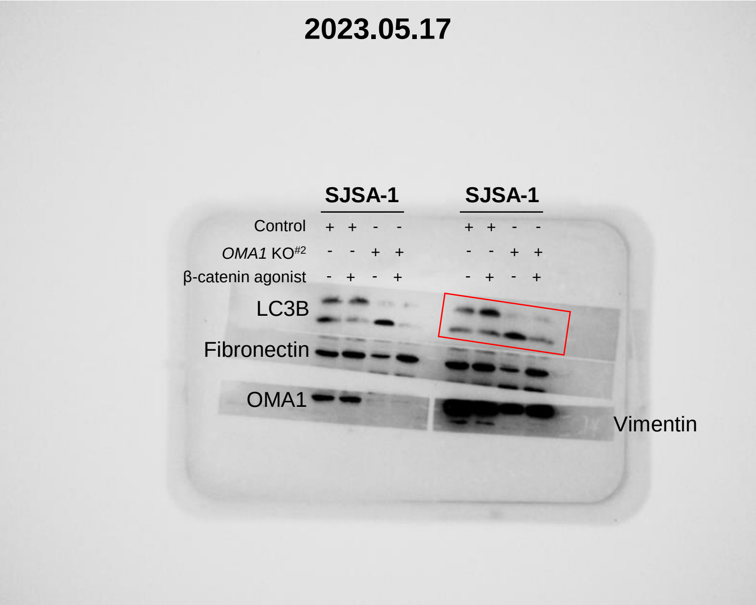
2023.05.17
SJSA-1
SJSA-1
Control
OMA1 KO#2
β-catenin agonist
LC3B
Fibronectin
OMA1
Vimentin

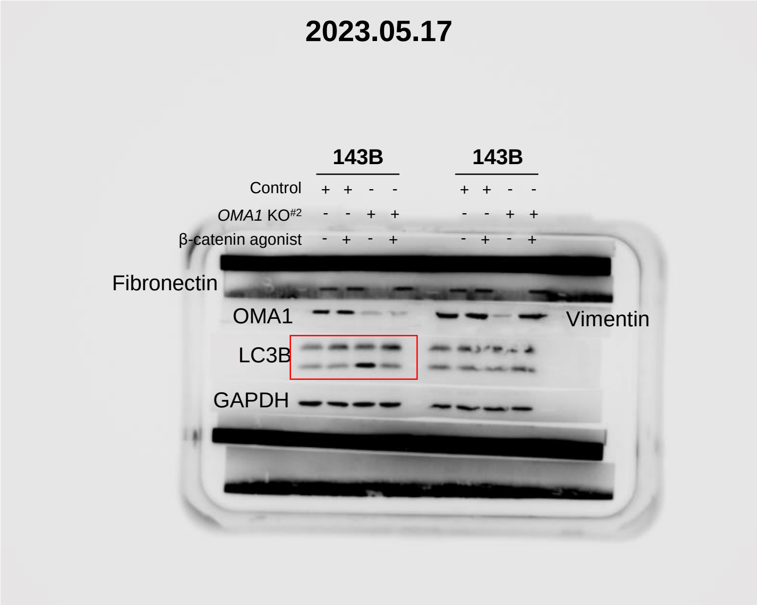
2023.05.17
143B
143B
Control
OMA1 KO#2
β-catenin agonist
Fibronectin
OMA1
Vimentin
LC3B
GAPDH

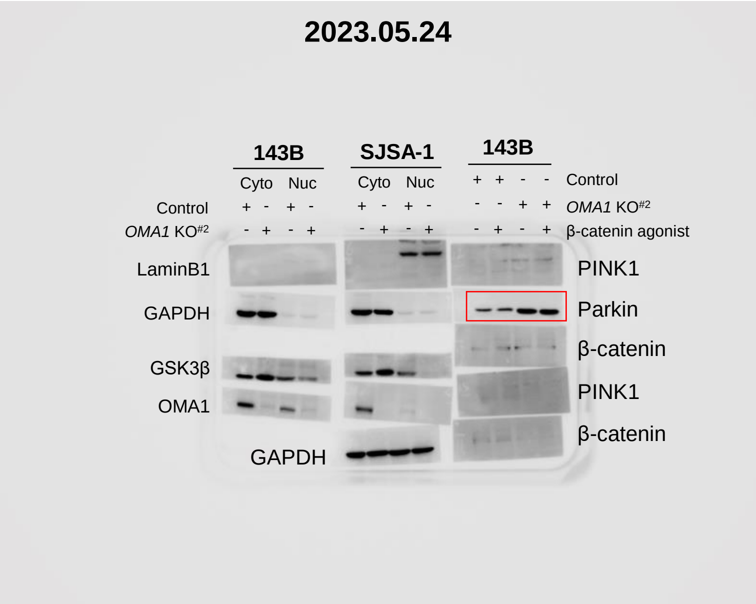
2023.05.24
143B
SJSA-1
143B
Cyto Nuc
Cyto Nuc
Control
OMA1 KO#2
β-catenin agonist
LaminB1
GAPDH
GSK3β
OMA1
GAPDH
PINK1
Parkin
β-catenin
PINK1
β-catenin

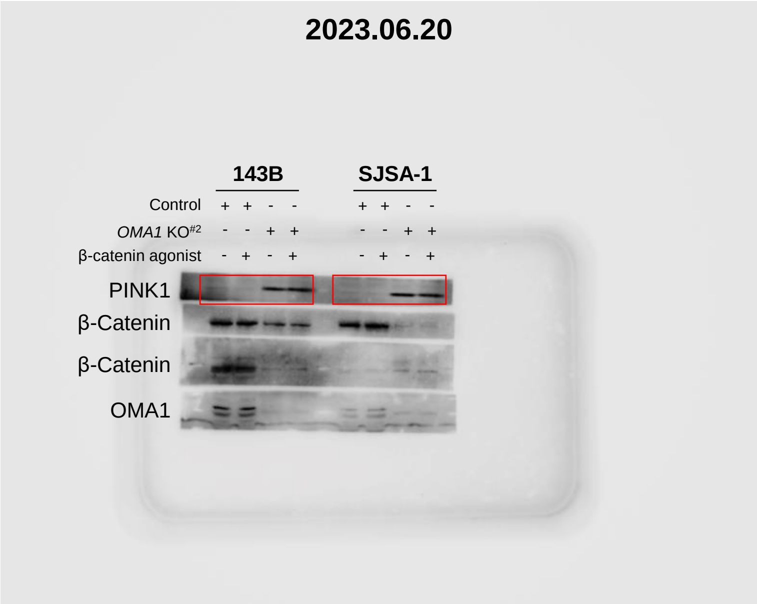
2023.06.20
143B
SJSA-1
Control
OMA1 KO#2
β-catenin agonist
PINK1
β-Catenin
β-Catenin
OMA1

Fig. 5I

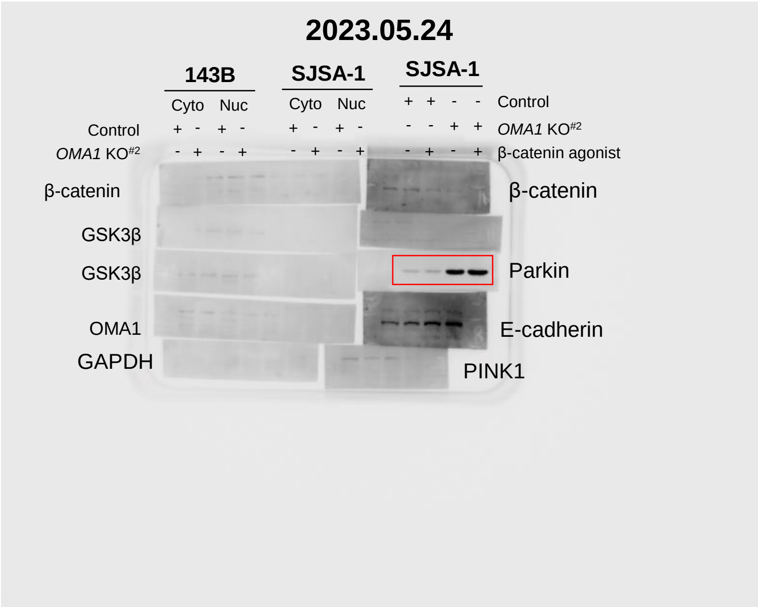

2023.05.24
143B
SJSA-1
SJSA-1
Cyto Nuc
Cyto Nuc
Control
OMA1 KO#2
β-catenin agonist
β-catenin
GSK3β
GSK3β
OMA1
GAPDH
β-catenin
Parkin
E-cadherin
PINK1

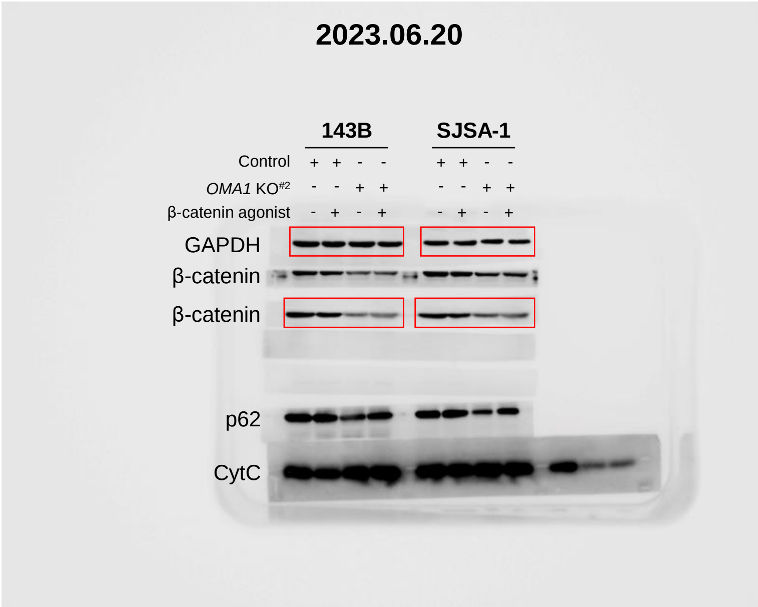

2023.06.20
143B
SJSA-1
Control
OMA1 KO#2
β-catenin agonist
GAPDH
β-catenin
β-catenin
p62
CytC

Fig. 6A

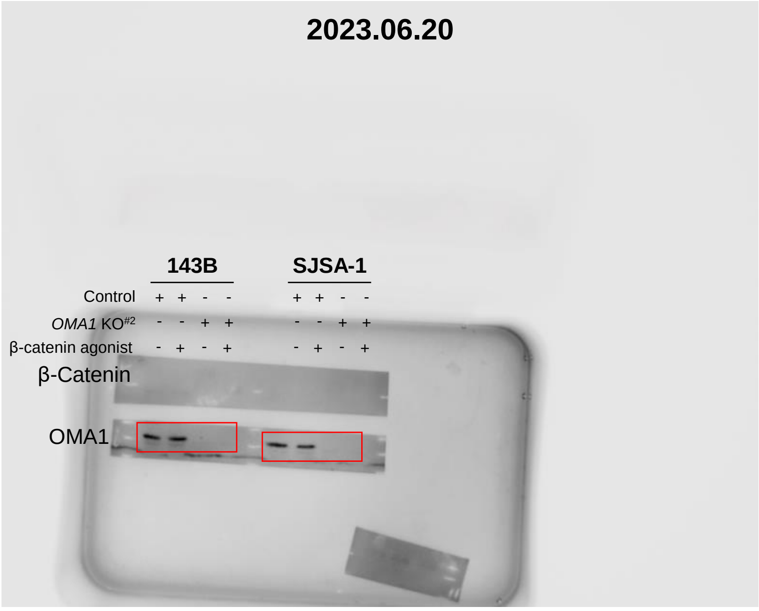

2023.06.20
143B
SJSA-1
Control
OMA1 KO#2
β-catenin agonist
β-Catenin
OMA1

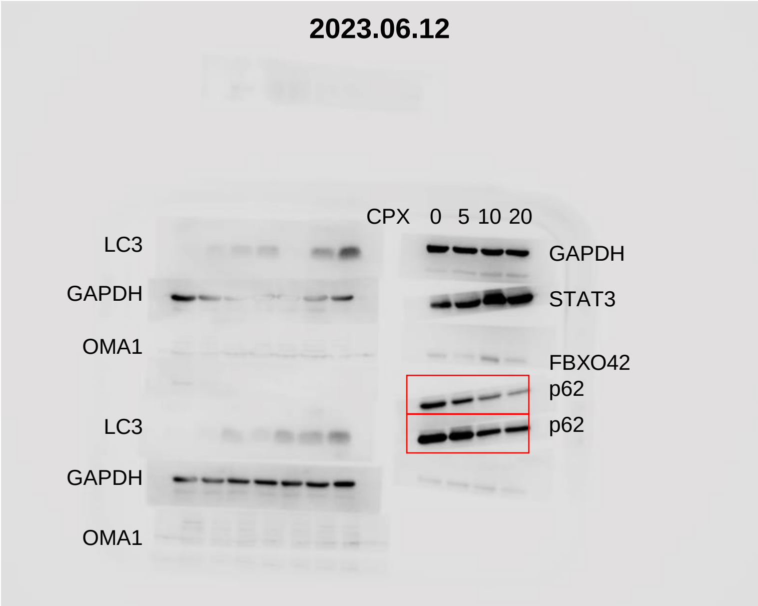

2023.06.12
CPX 0 5 10 20
LC3
GAPDH
OMA1
LC3
GAPDH
OMA1
GAPDH
STAT3
FBXO42
p62
p62

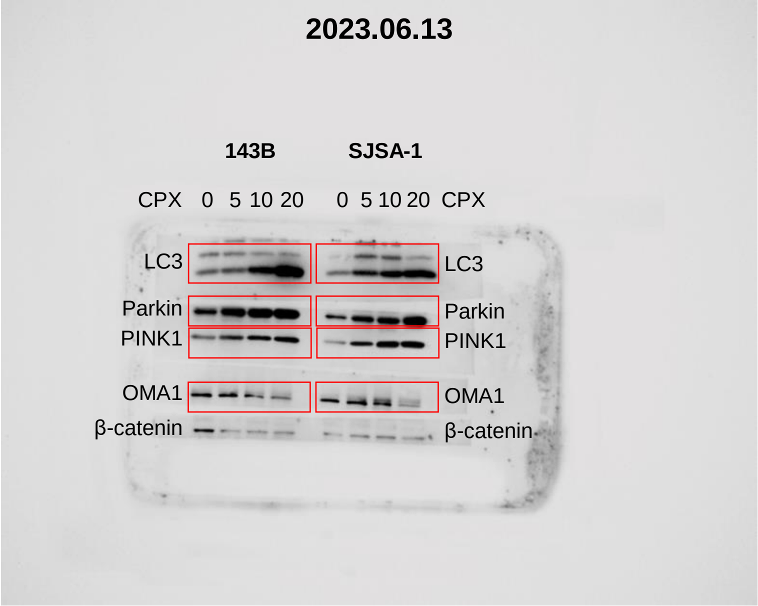

2023.06.13
143B
SJSA-1
CPX 0 5 10 20
0 5 10 20 CPX
LC3
Parkin
PINK1
OMA1
β-catenin
LC3
Parkin
PINK1
OMA1
β-catenin

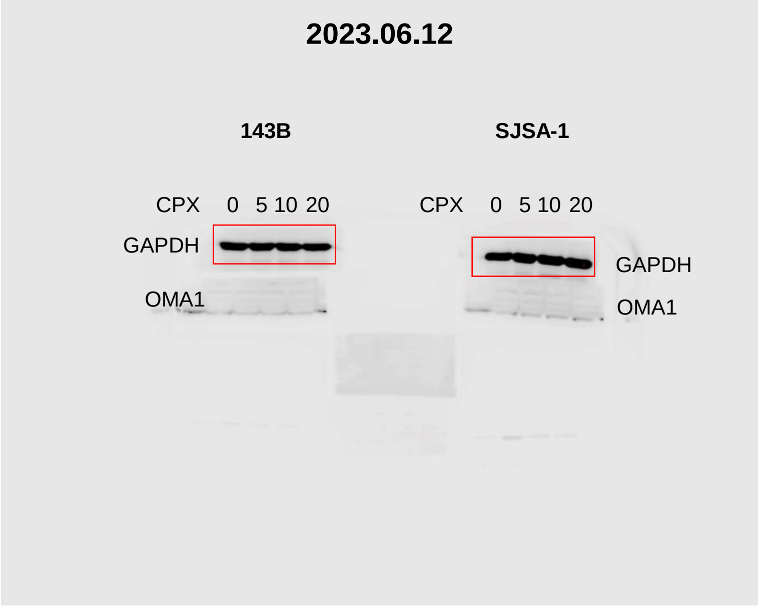

2023.06.12
143B
SJSA-1
CPX 0 5 10 20
CPX 0 5 10 20
GAPDH
OMA1
GAPDH
OMA1

Fig. 6C

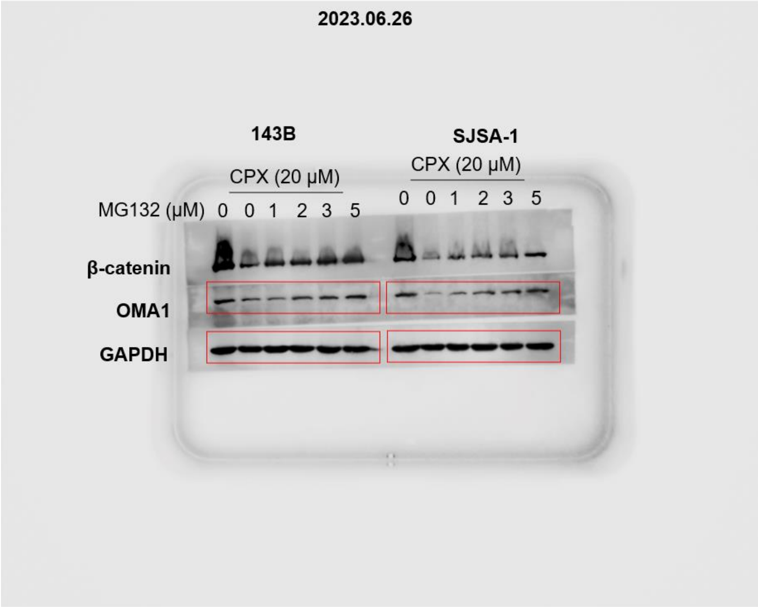

Fig. 6D

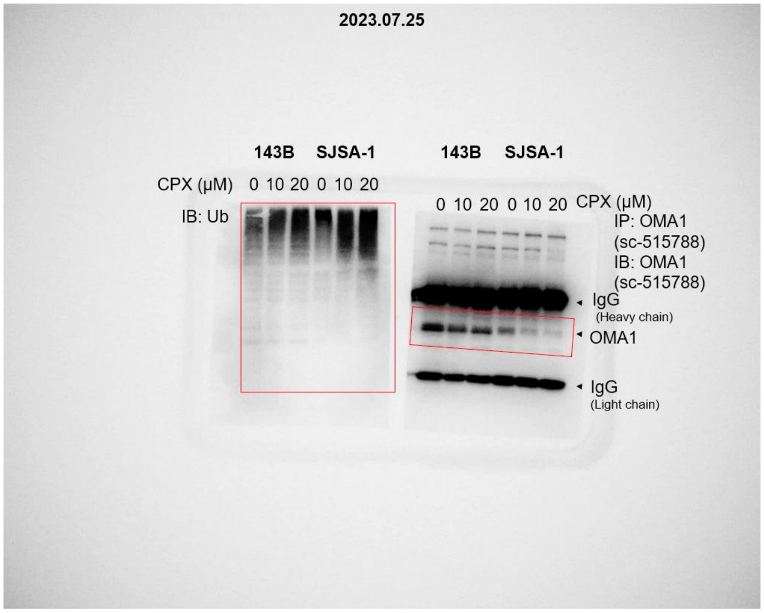

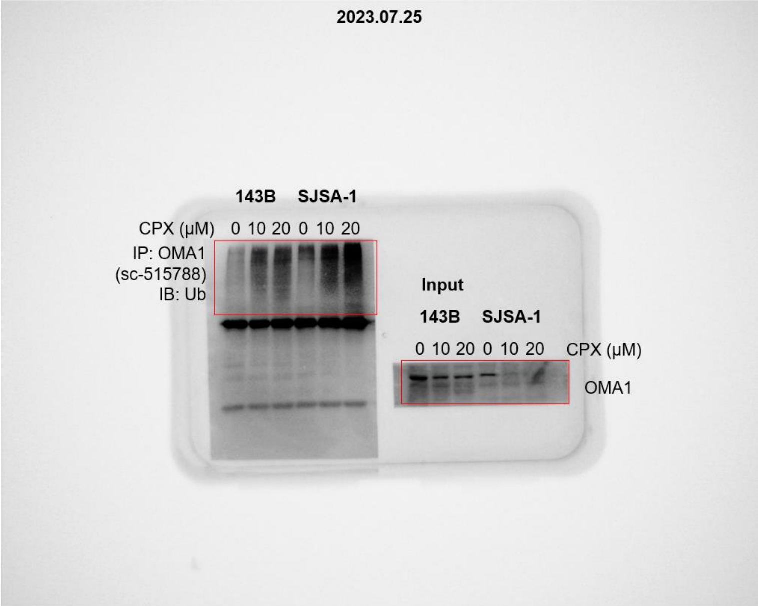

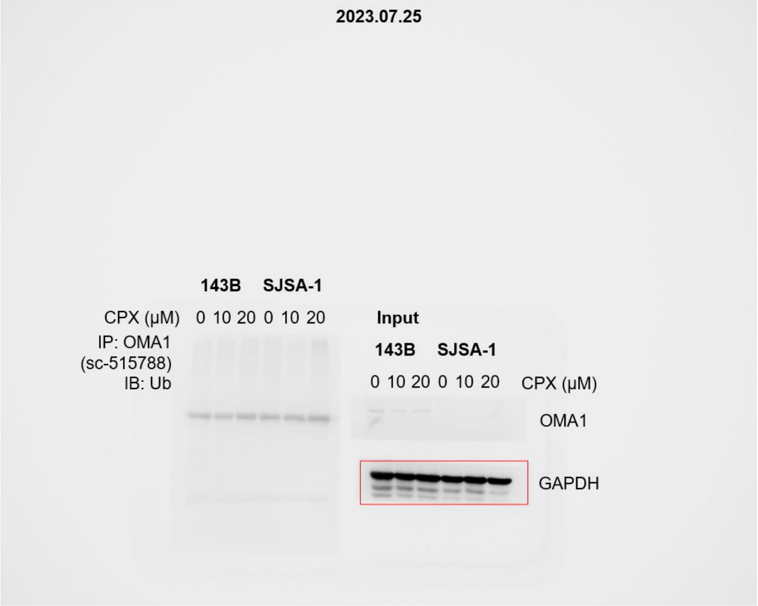

Fig. 6E

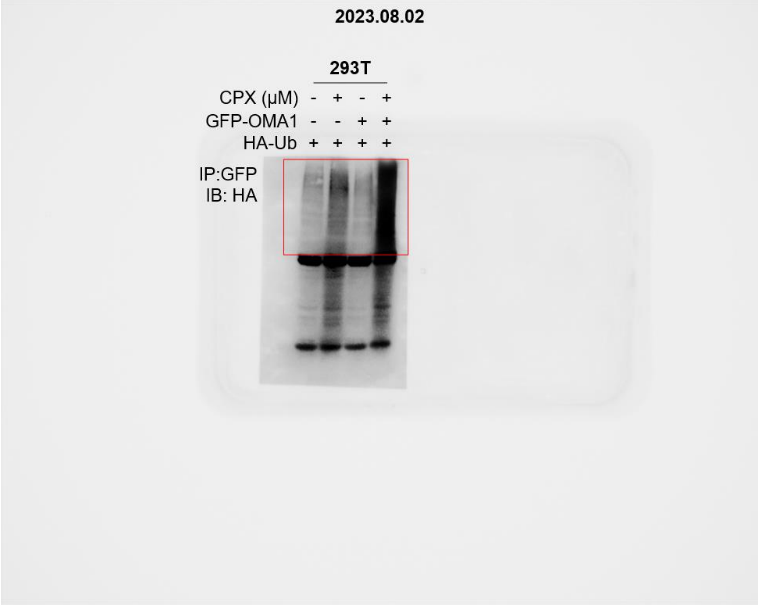

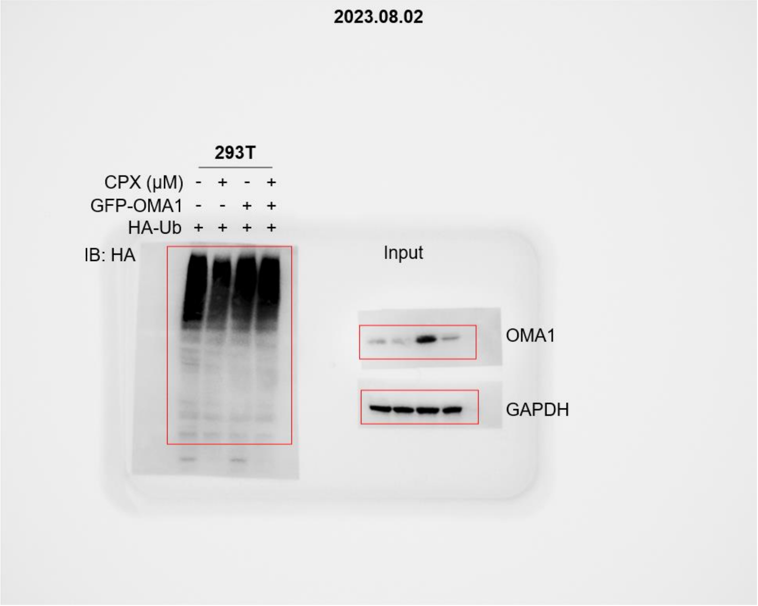

Fig. 6E

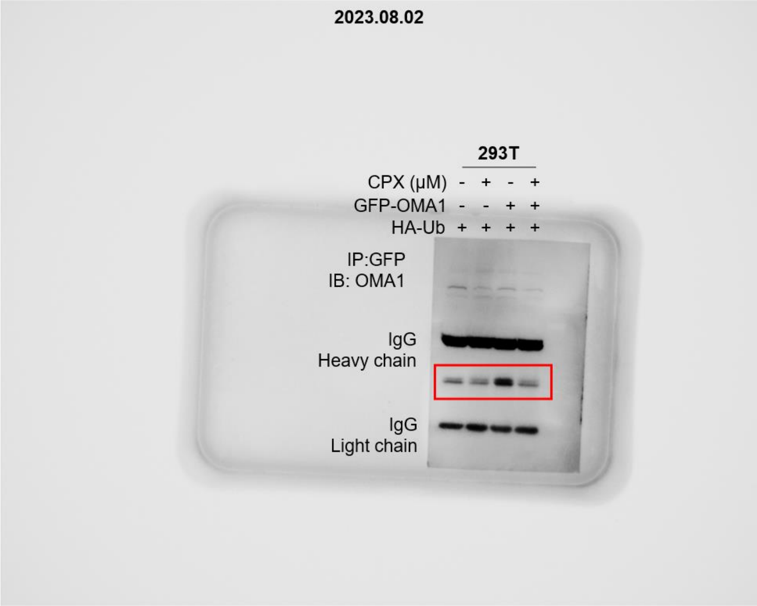

Fig. 6F

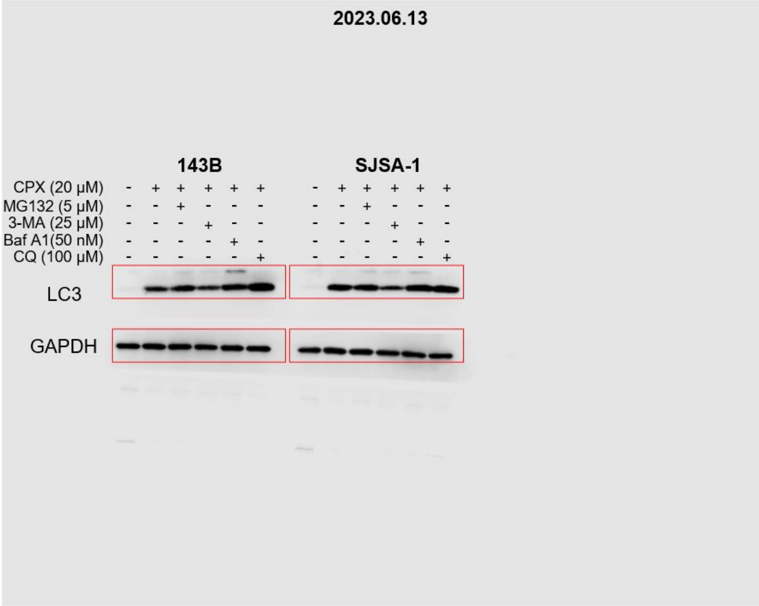

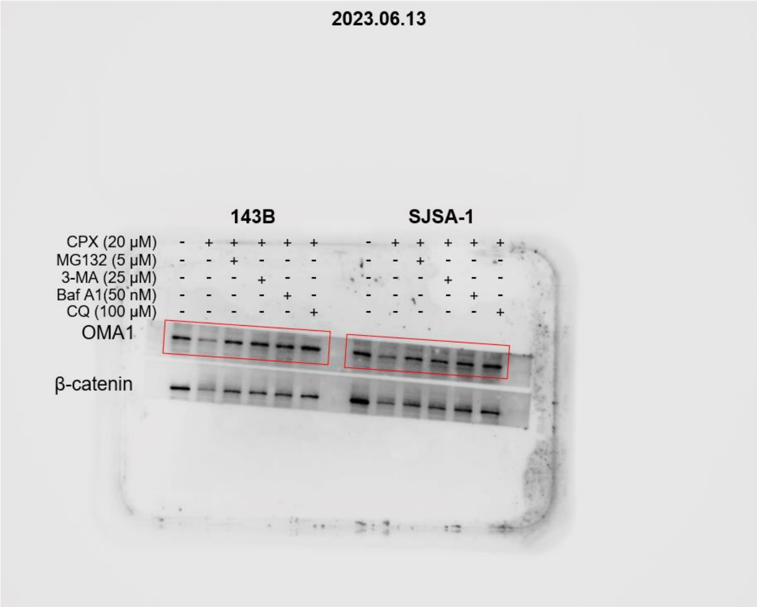

Fig. S1c

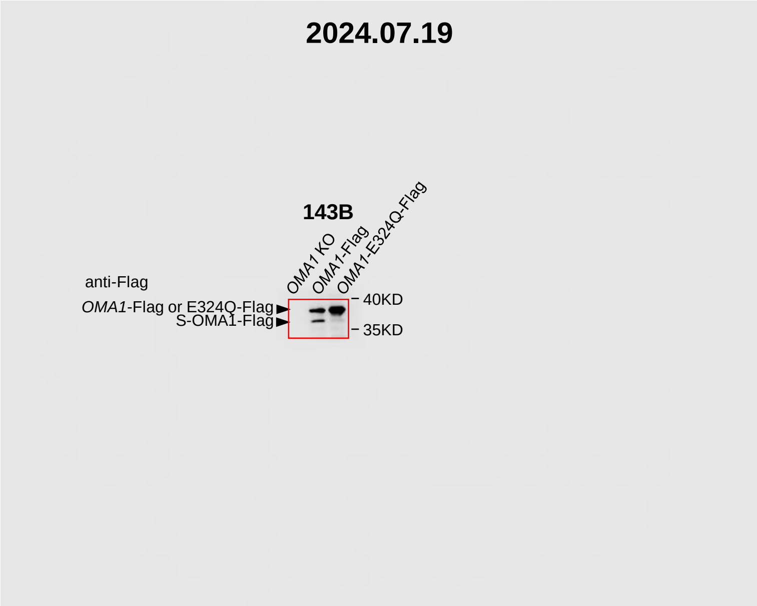

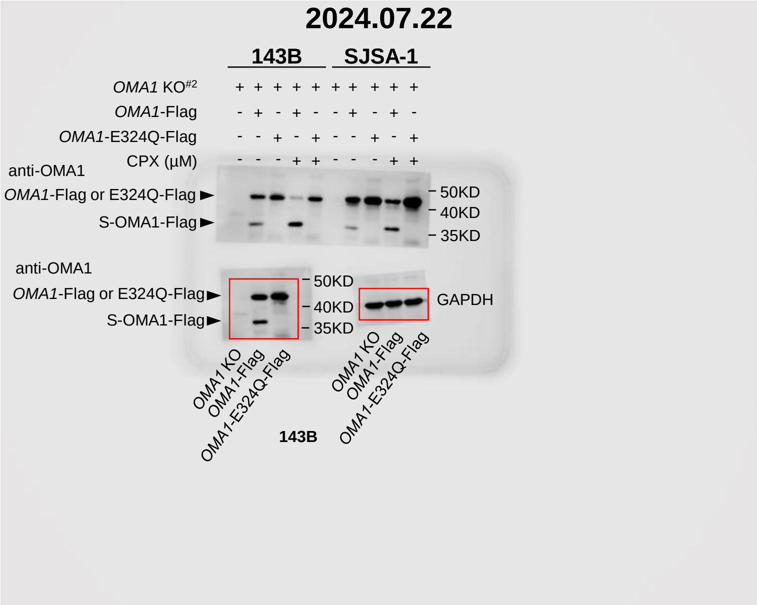

Fig. S2a

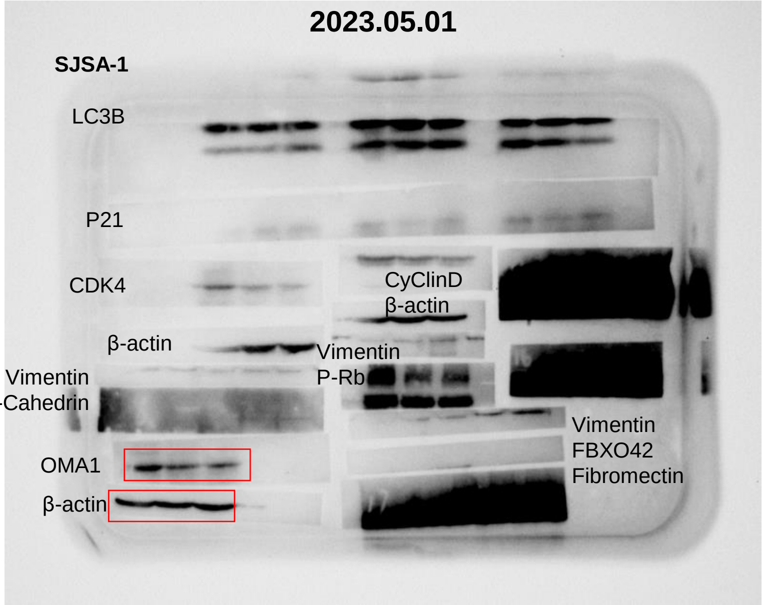

Fig. S2a

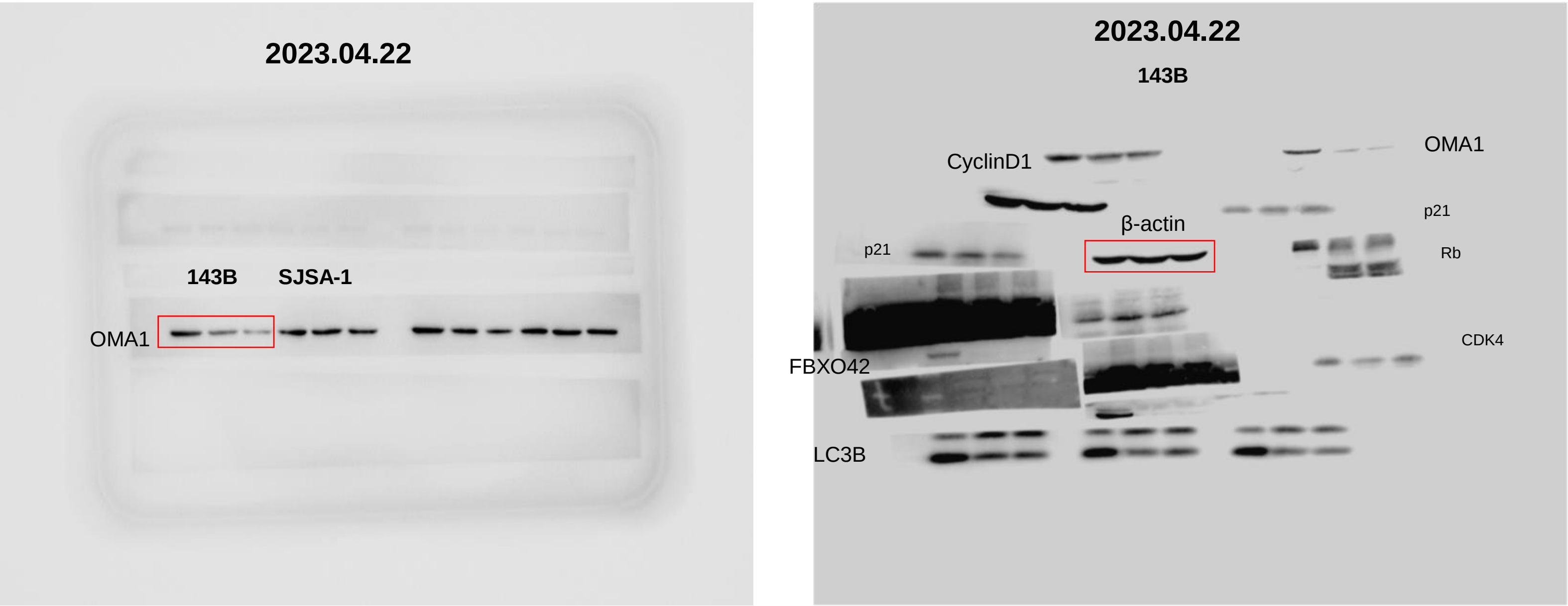

Fig. S3g

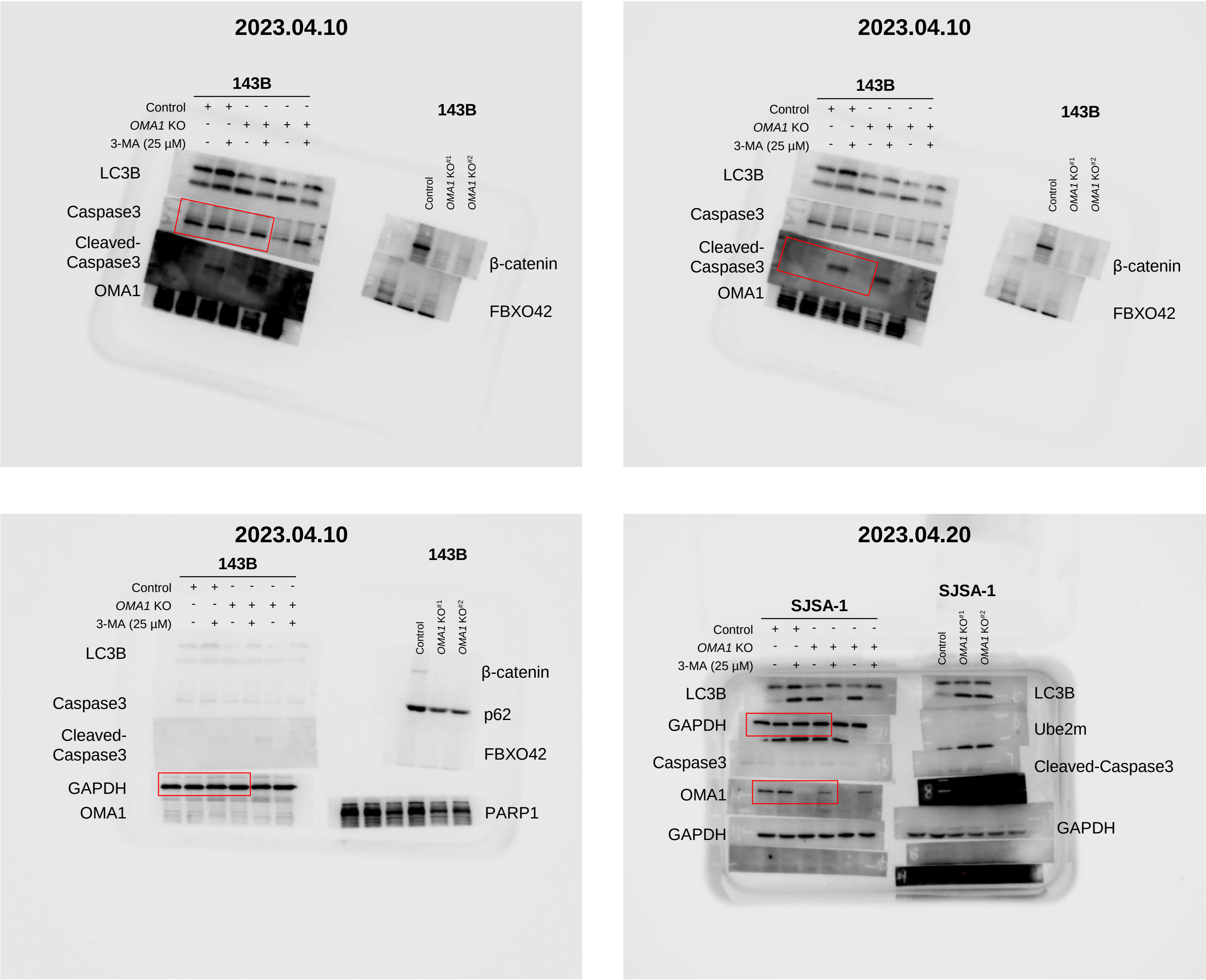

Fig. S3g

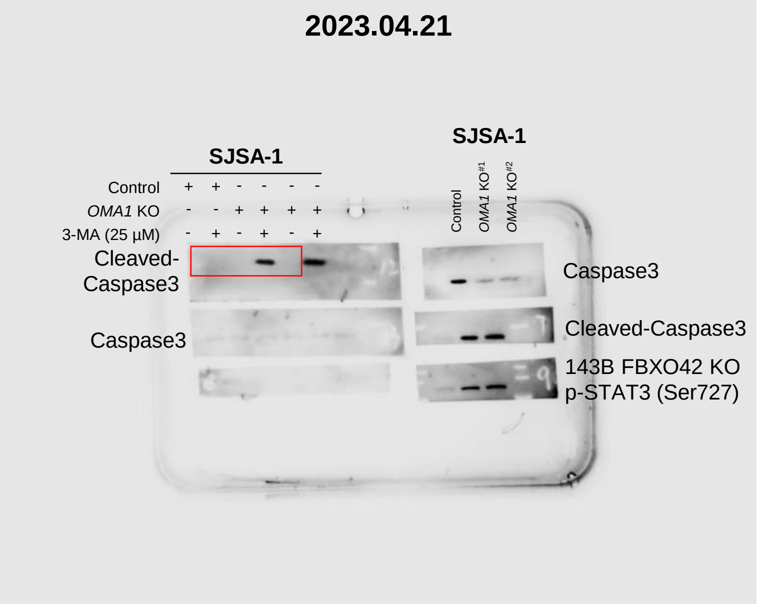

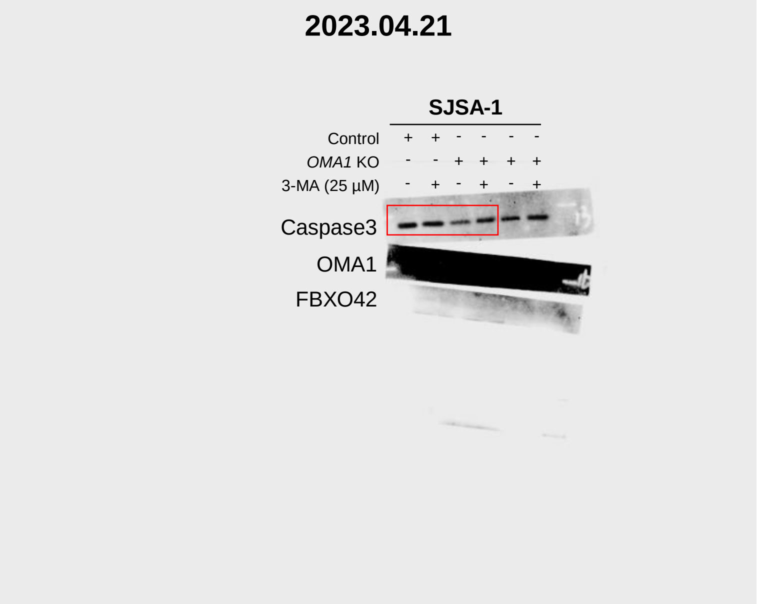

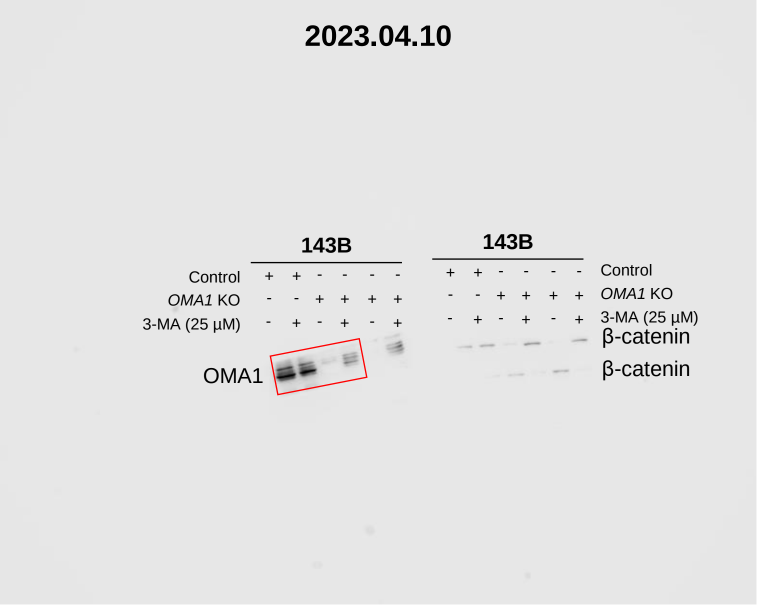

Fig. S3h

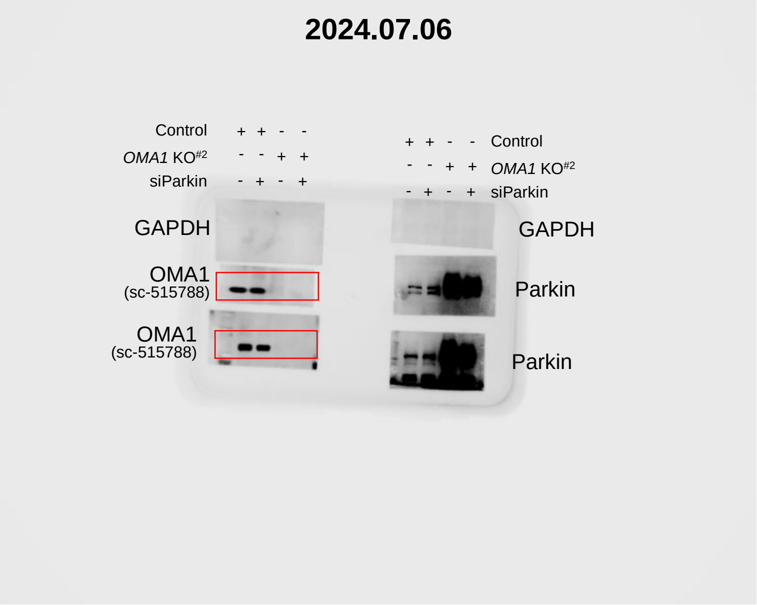

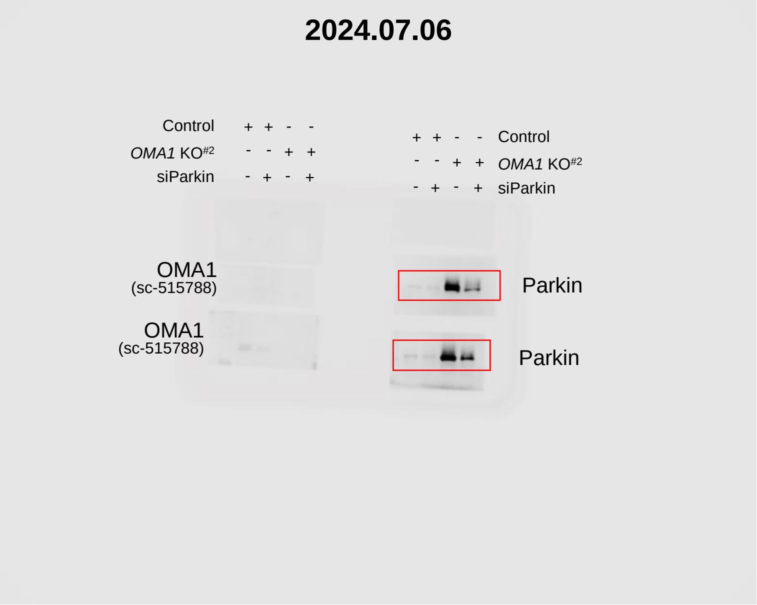

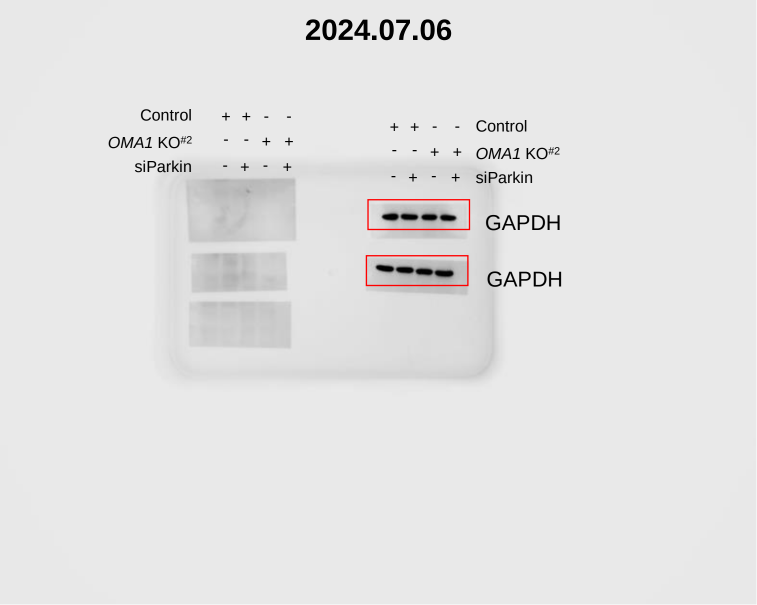

Fig. S4a

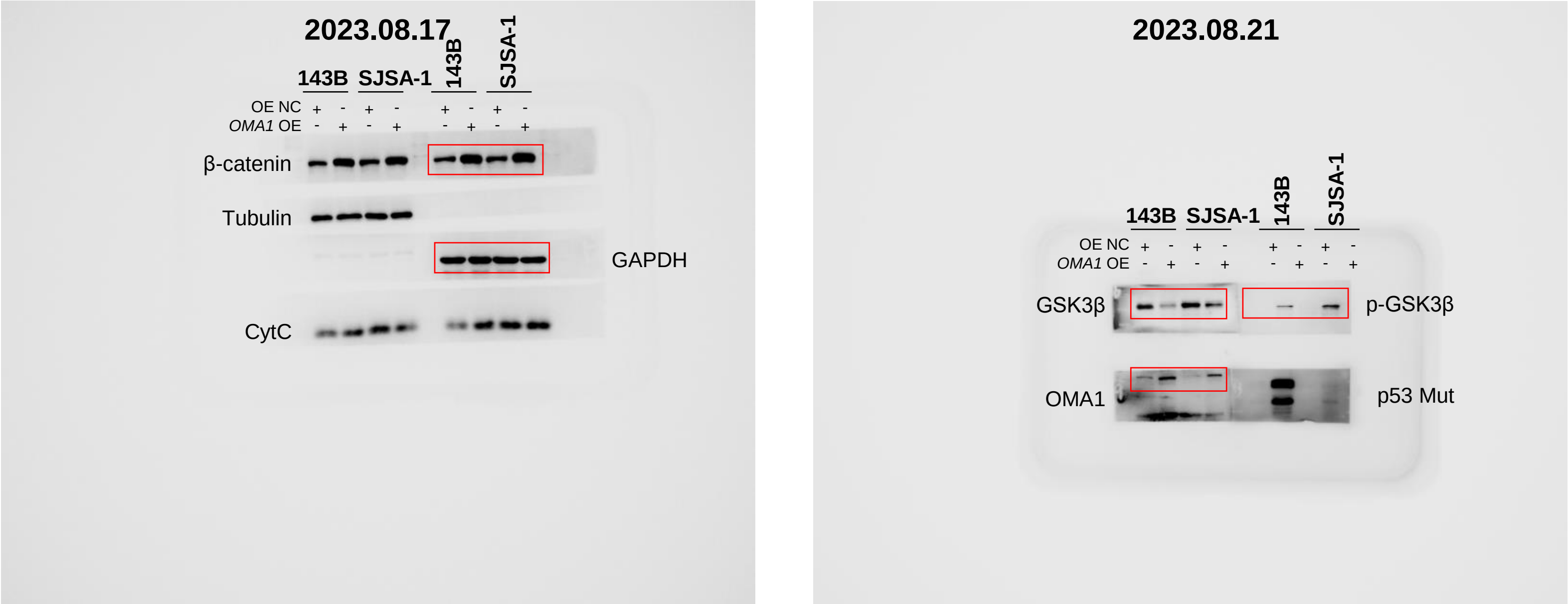

Fig. S4b

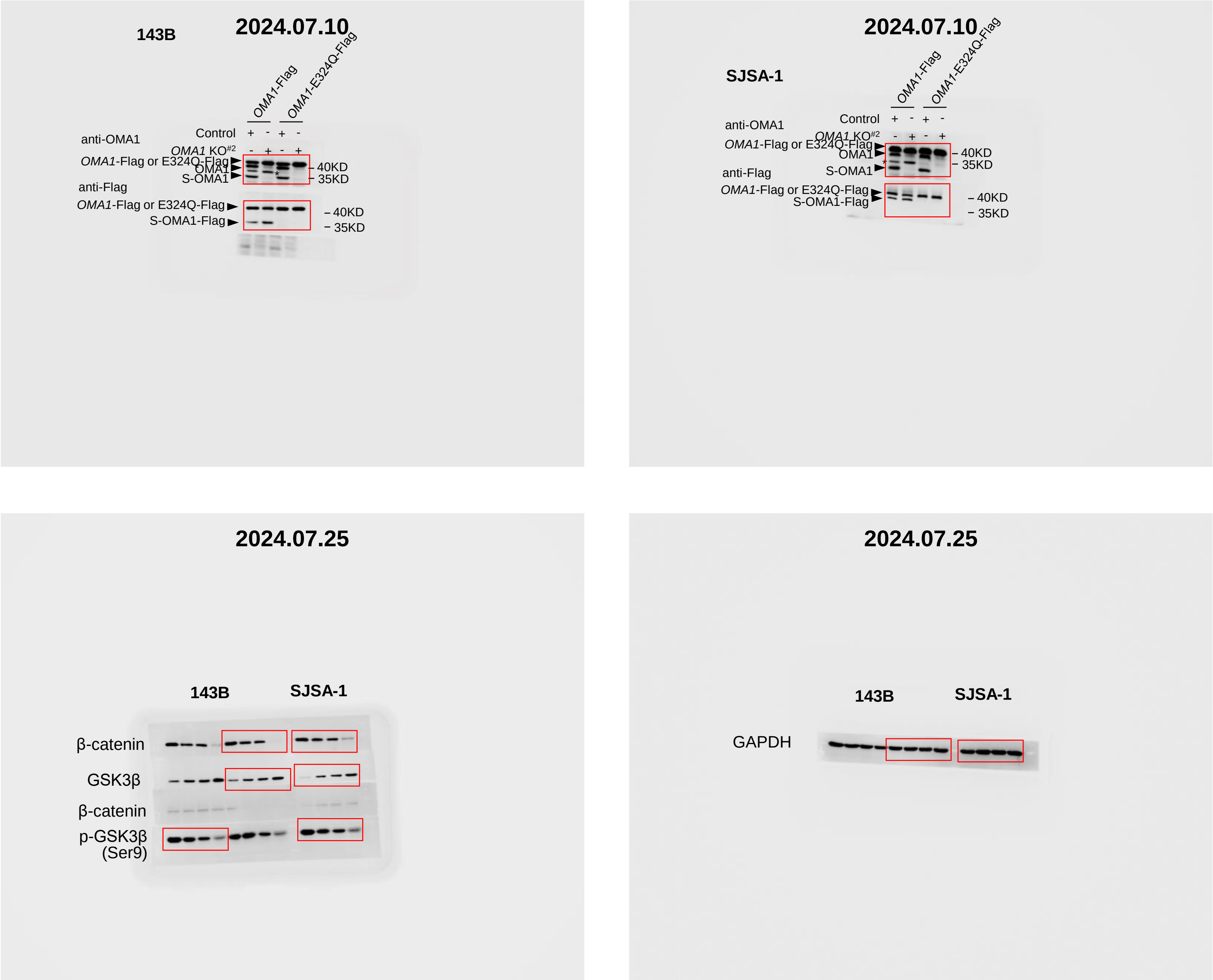

Fig. S4e

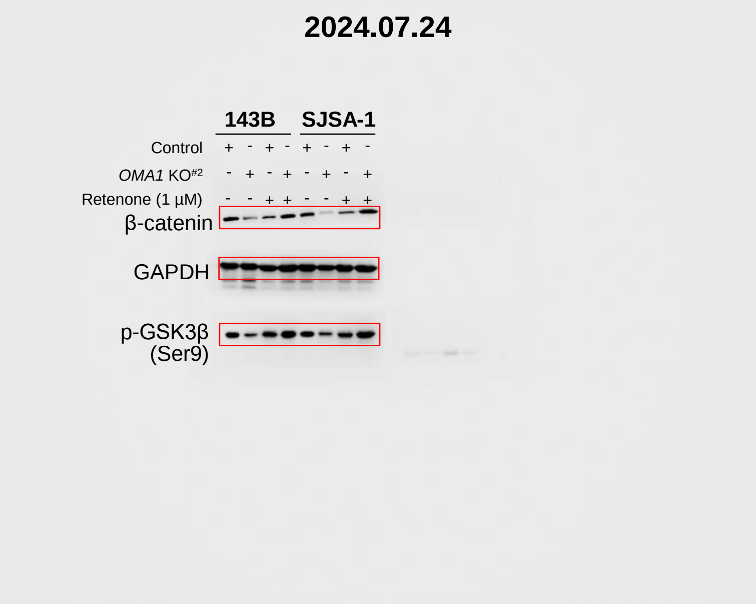

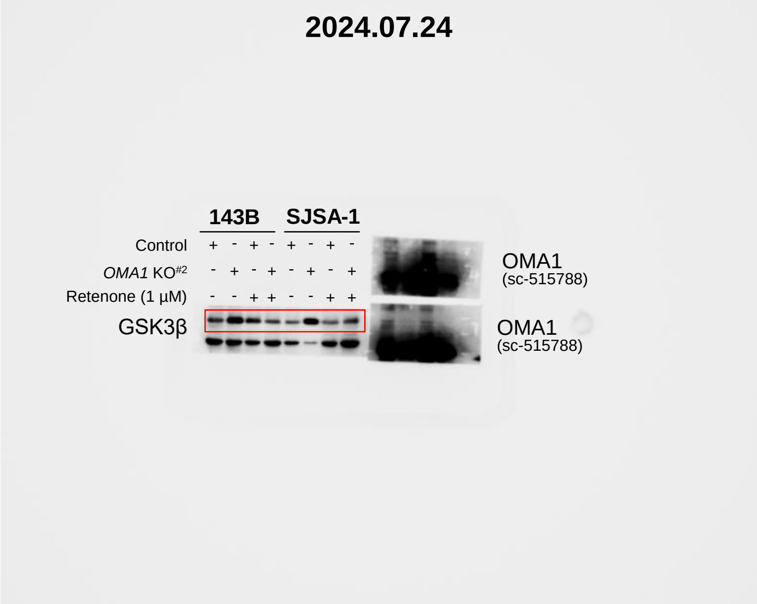

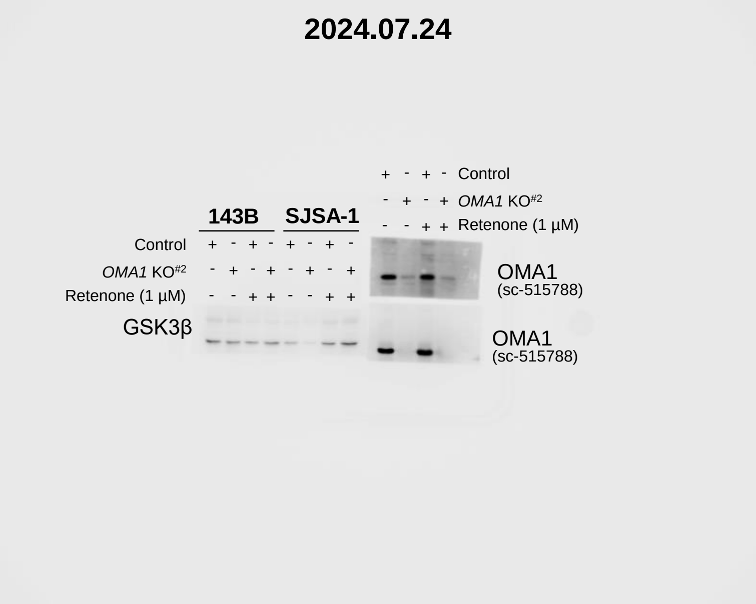

Fig. S5a

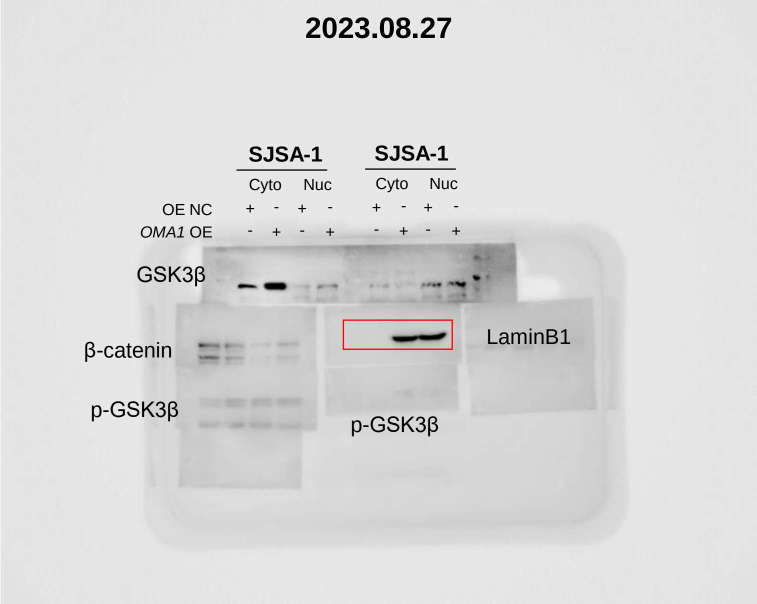

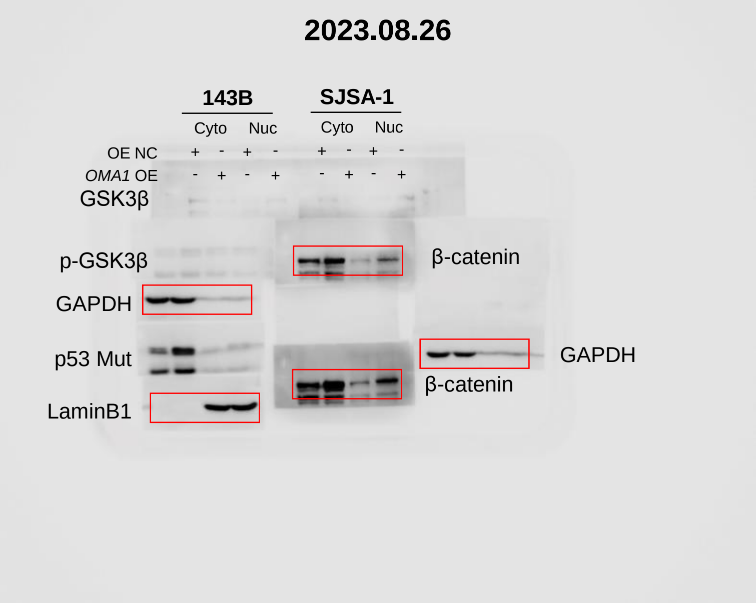

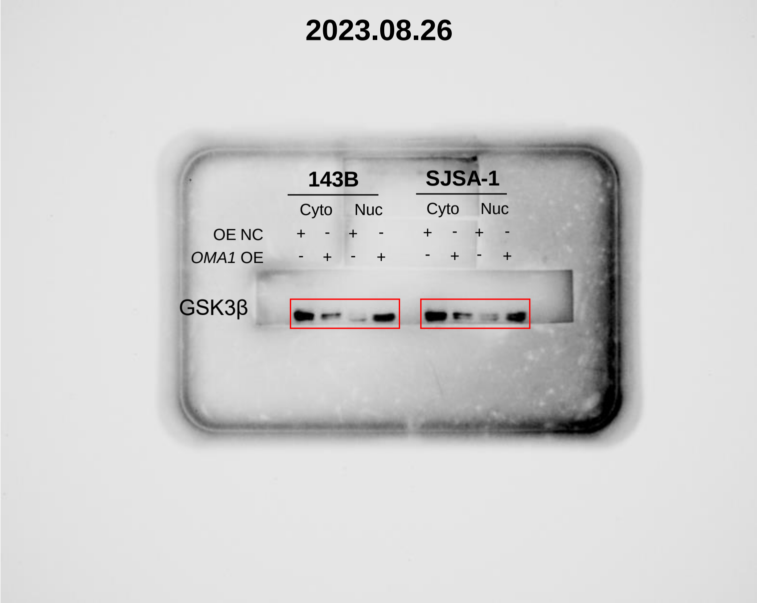

Fig. S5a

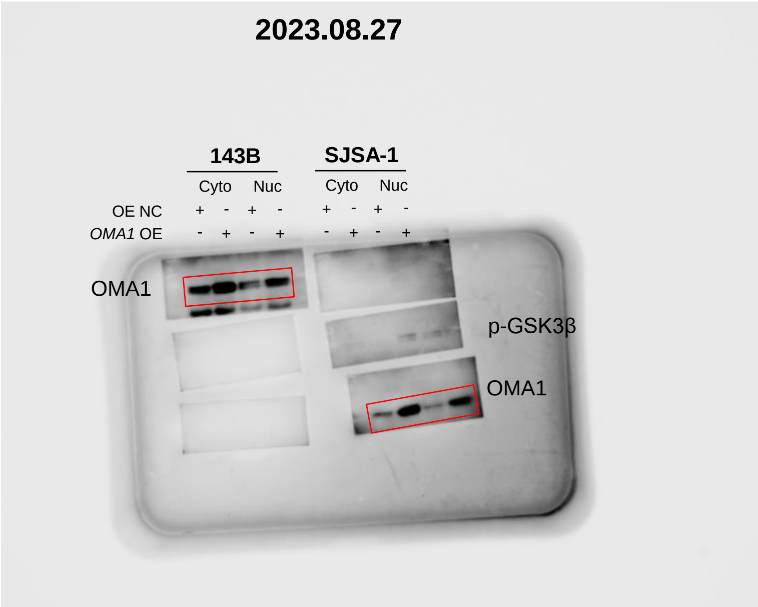

Fig. S6e

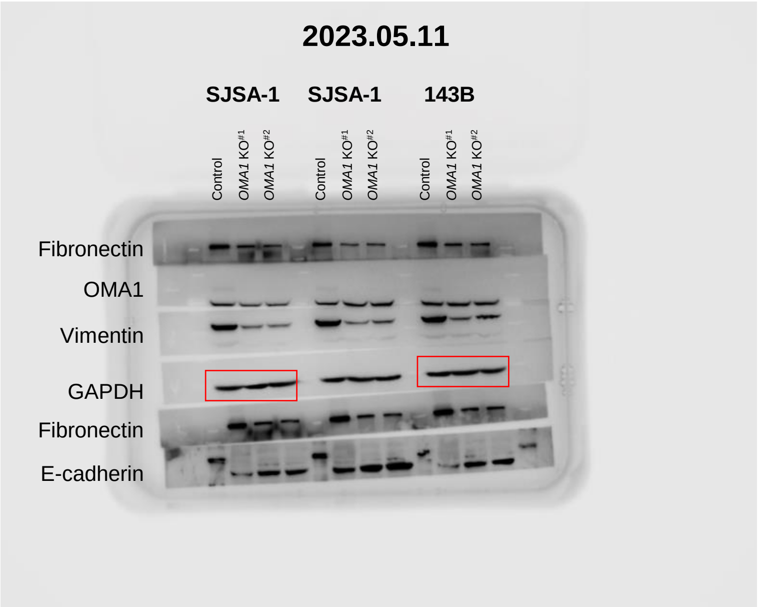

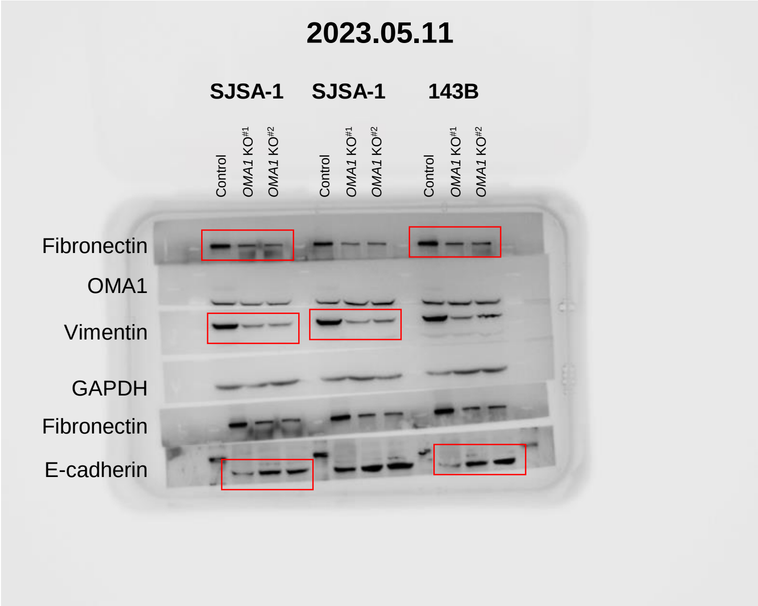

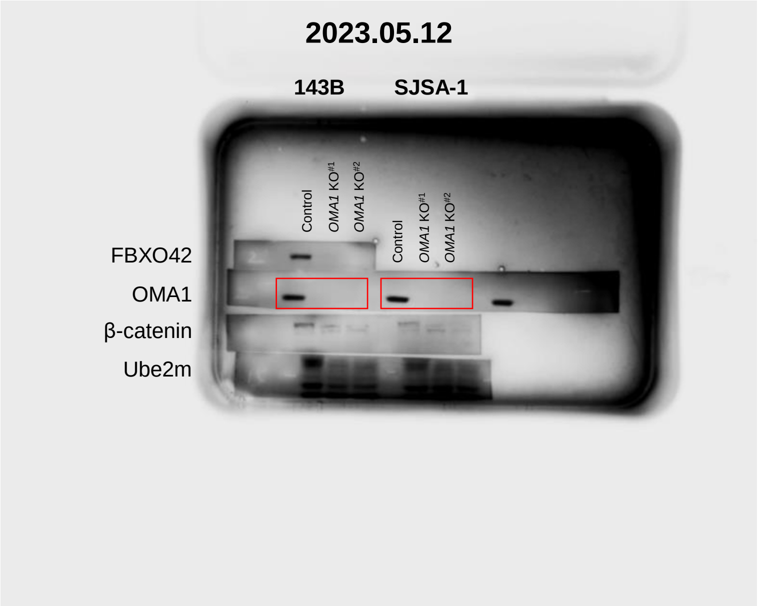

Fig. S7c

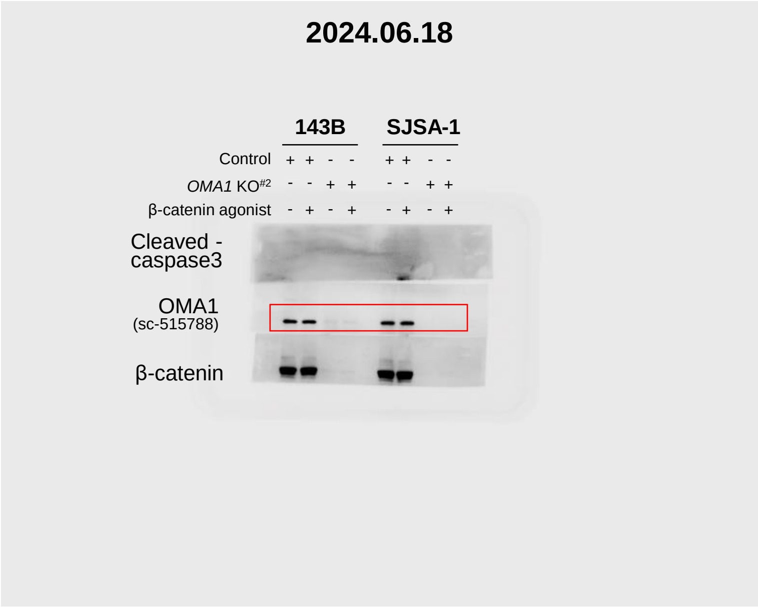

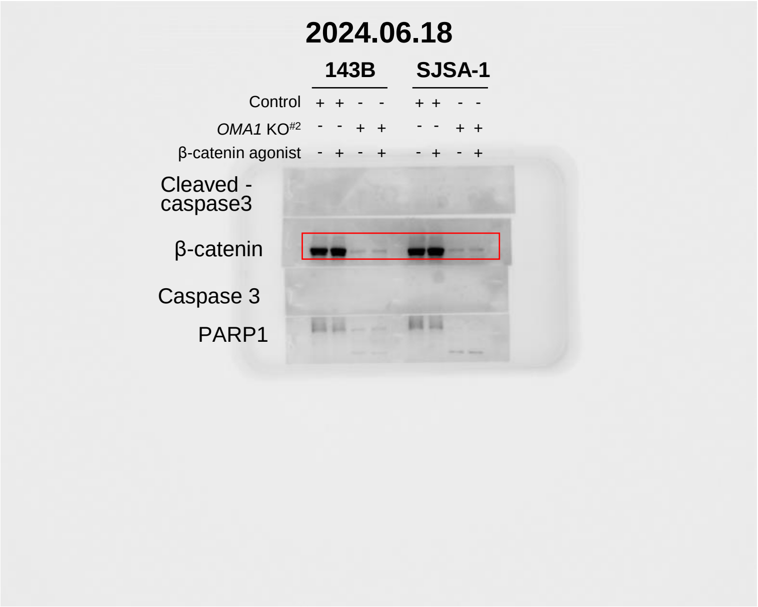

Fig. S7c

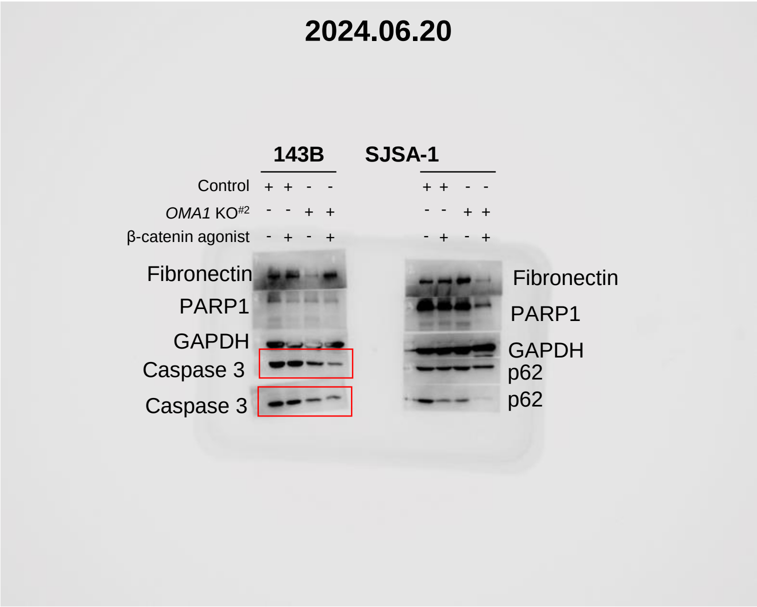

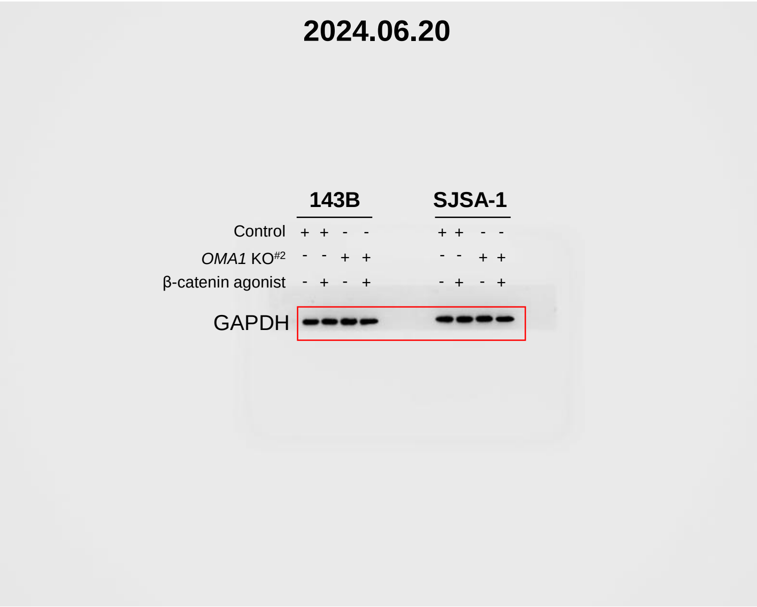

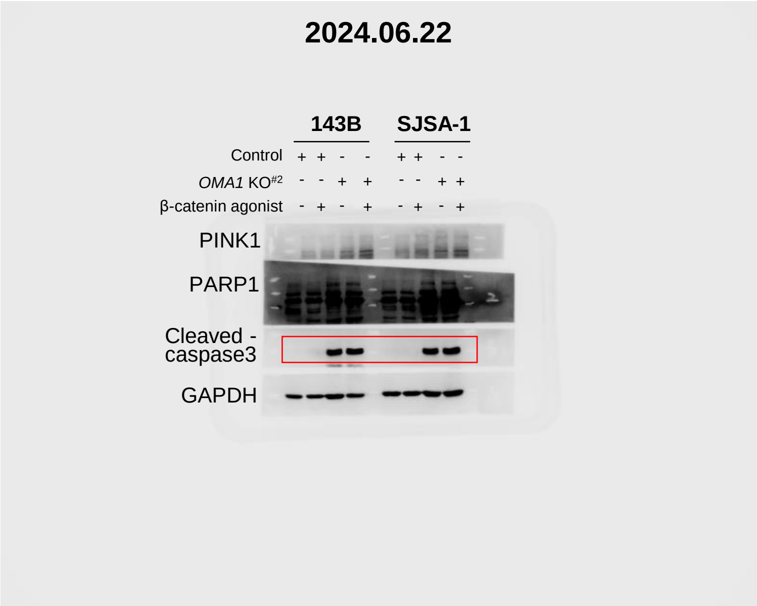

Fig. S7d

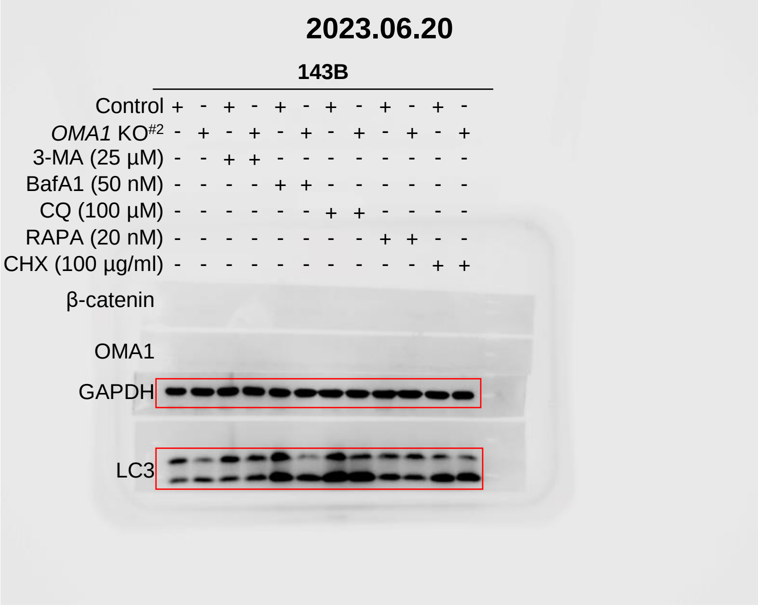

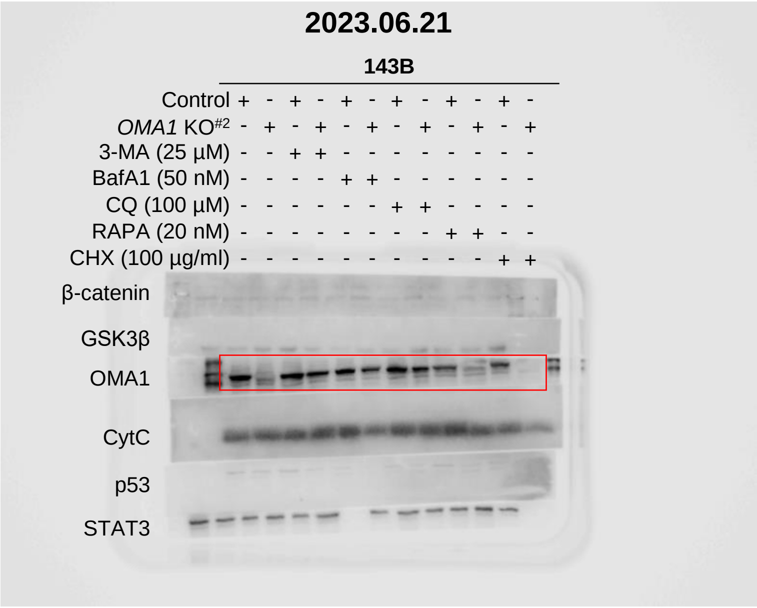

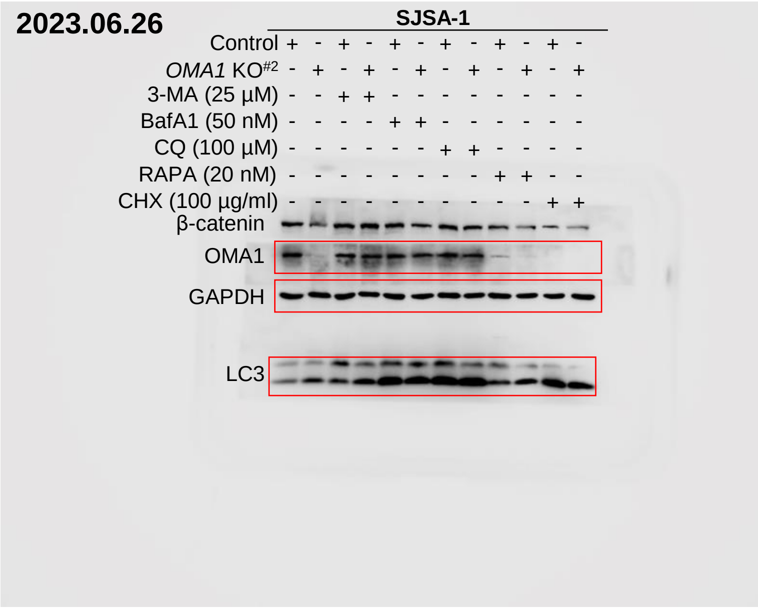

Fig. S9a

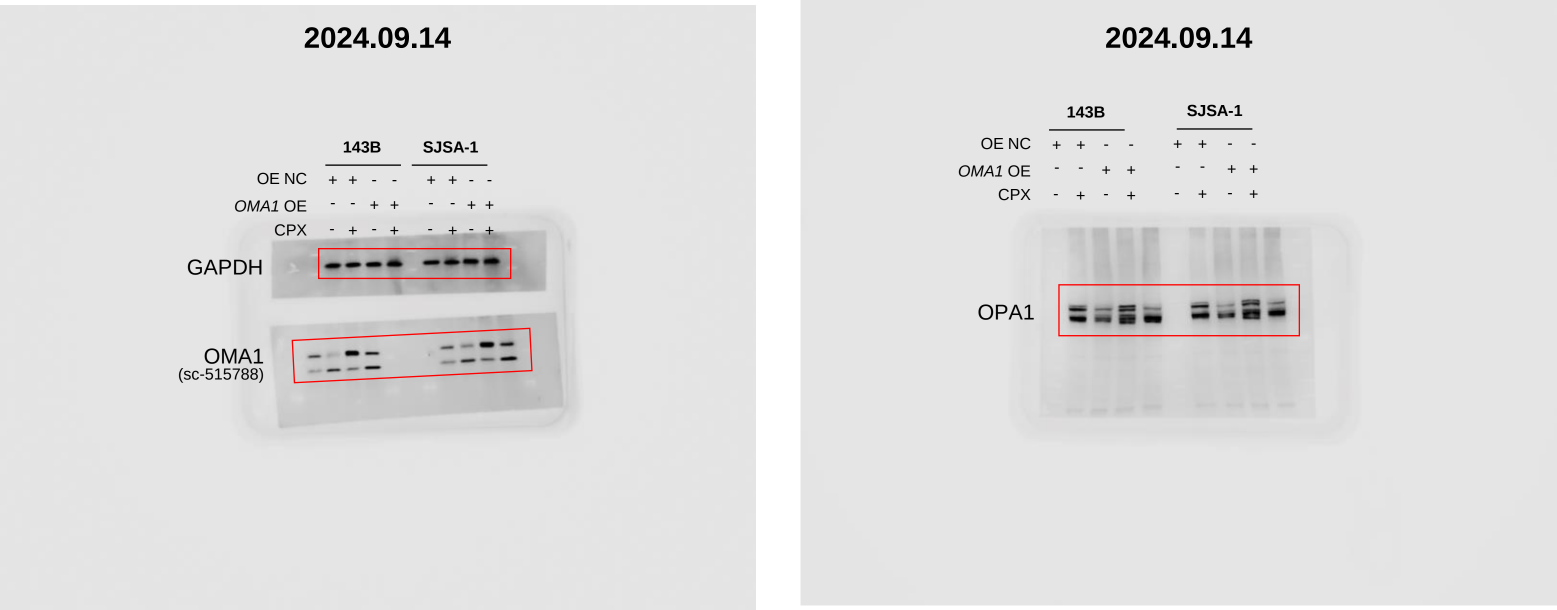

Fig. S9b

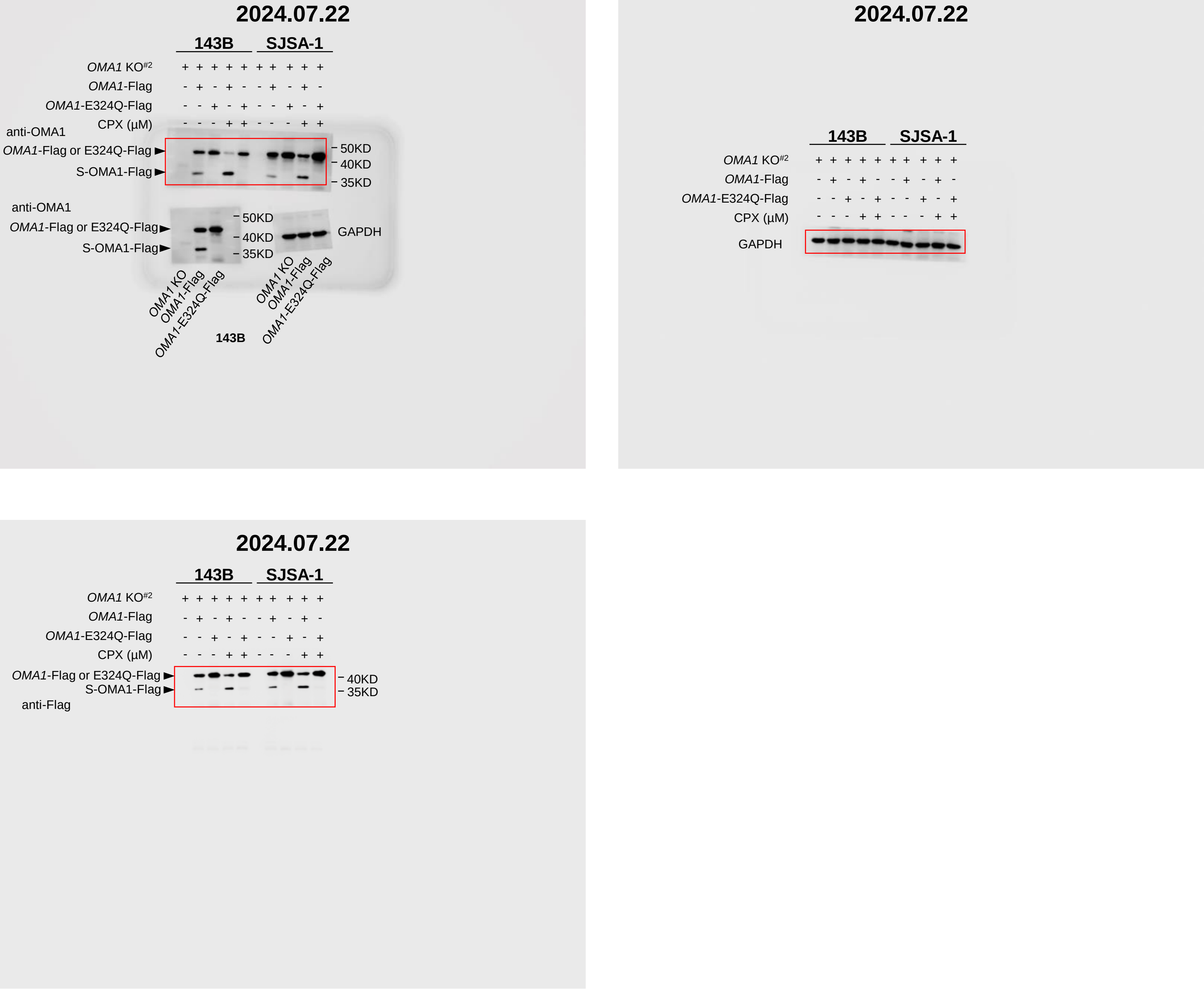

Supplement: Supplementary file 2 — Original Data [file 41419_2024_7127_MOESM2_ESM.pdf]
